# Integrated analyses for genetic markers of polycystic ovary syndrome with 9 case-control studies of gene expression profiles

**DOI:** 10.18632/oncotarget.13881

**Published:** 2016-12-10

**Authors:** Chenqi Lu, Xiaoqin Liu, Lin Wang, Ning Jiang, Jun Yu, Xiaobo Zhao, Hairong Hu, Saihua Zheng, Xuelian Li, Guiying Wang

**Affiliations:** ^1^ Department of Biostatistics and Computational Biology, State Key Laboratory of Genetic Engineering, Department of Gynecology, Obstetrics and Gynecology Hospital, School of Life Sciences, Fudan University, Shanghai, China; ^2^ Clinical and Translational Research Center of Shanghai First Maternity and Infant Health Hospital, Shanghai Key Laboratory of Signaling and Disease Research, School of Life Science and Technology, Tongji University, Shanghai, China; ^3^ Department of Endocrinology, East Hospital, Tongji University School of Medicine, Tongji University, Shanghai, China

**Keywords:** polycystic ovary syndrome, common markers, integrated analysis, susceptibility, gene expression profile

## Abstract

Due to genetic heterogeneity and variable diagnostic criteria, genetic studies of polycystic ovary syndrome are particularly challenging. Furthermore, lack of sufficiently large cohorts limits the identification of susceptibility genes contributing to polycystic ovary syndrome. Here, we carried out a systematic search of studies deposited in the Gene Expression Omnibus database through August 31, 2016. The present analyses included studies with: 1) patients with polycystic ovary syndrome and normal controls, 2) gene expression profiling of messenger RNA, and 3) sufficient data for our analysis. Ultimately, a total of 9 studies with 13 datasets met the inclusion criteria and were performed for the subsequent integrated analyses. Through comprehensive analyses, there were 13 genetic factors overlapped in all datasets and identified as significant specific genes for polycystic ovary syndrome. After quality control assessment, there were six datasets remained. Further gene ontology enrichment and pathway analyses suggested that differentially expressed genes mainly enriched in oocyte pathways. These findings provide potential molecular markers for diagnosis and prognosis of polycystic ovary syndrome, and need in-depth studies on the exact function and mechanism in polycystic ovary syndrome.

## INTRODUCTION

Polycystic ovary syndrome (PCOS), as a highly complex endocrine disorder, is usually comprised of phenotypical and heterogeneous reproductive effects [1] as well as metabolic symptoms [2–5]. Thus, there is great genetic heterogeneity and different pathophysiological mechanisms of various PCOS phenotypes. The genetic heterogeneity, combined with the pronounced variability in the diagnostic criteria, makes the genetic study and susceptibility gene identification of PCOS particularly difficult. Although PCOS has been studied for decades, the genetic contributions to this disorder are not fully understood. Furthermore, lack of sufficiently large cohorts also reduces the power to identify specific genes of PCOS [6].

It has been reported that candidate genes associated with PCOS contribute to different biological processes and phenotypes [7]. For example, as the association between obesity and PCOS [8], gene variants affecting fat mass, such as FTO [9], have been found to be involved in PCOS. The effect of common variants in TCF7L2 and KCNJ11 is likely to be mediated by the impairment of insulin secretion from β-cells, as a well-established pathogenic pathway in PCOS [10]. The hyperandrogenemia of PCOS is most commonly characterized by increased testosterone levels, resulting from enhanced ovarian biosynthesis [11]. Several genes (CYP11A, CYP19) involved in steroid biosynthesis pathways are also potential candidate genes for PCOS. However, the candidate genetic markers significantly contributing to the diagnosis and prognosis of PCOS needs to be further explored.

Recently, gene expression profiling with microarray and RNA sequencing has been used to discover gene markers and signaling pathways associated with various complex diseases [12]. Gene expression datasets from PCOS patients and normal controls have been collected for the successful identification of gene expression signatures [13]. However, as the extreme heterogeneity and small sample size among studies, the reproducibility of various studies is very low [14]. If a larger sample size is not attainable, integrated analyses of multi-center collaborative studies become very useful [15].

In this study, to investigate the candidate diagnostic and prognostic genetic markers for PCOS, we performed comprehensive and statistical analyses on 9 case-control studies with 13 gene expression profiling datasets. Finally, we identified 13 genetic markers may be potential molecular factors for the diagnosis and prognosis of PCOS patients. Further functional studies of these candidate genes may improve the understanding and treatment of PCOS diseases.

## RESULTS

### Characteristics of included studies

**Figure 1:**
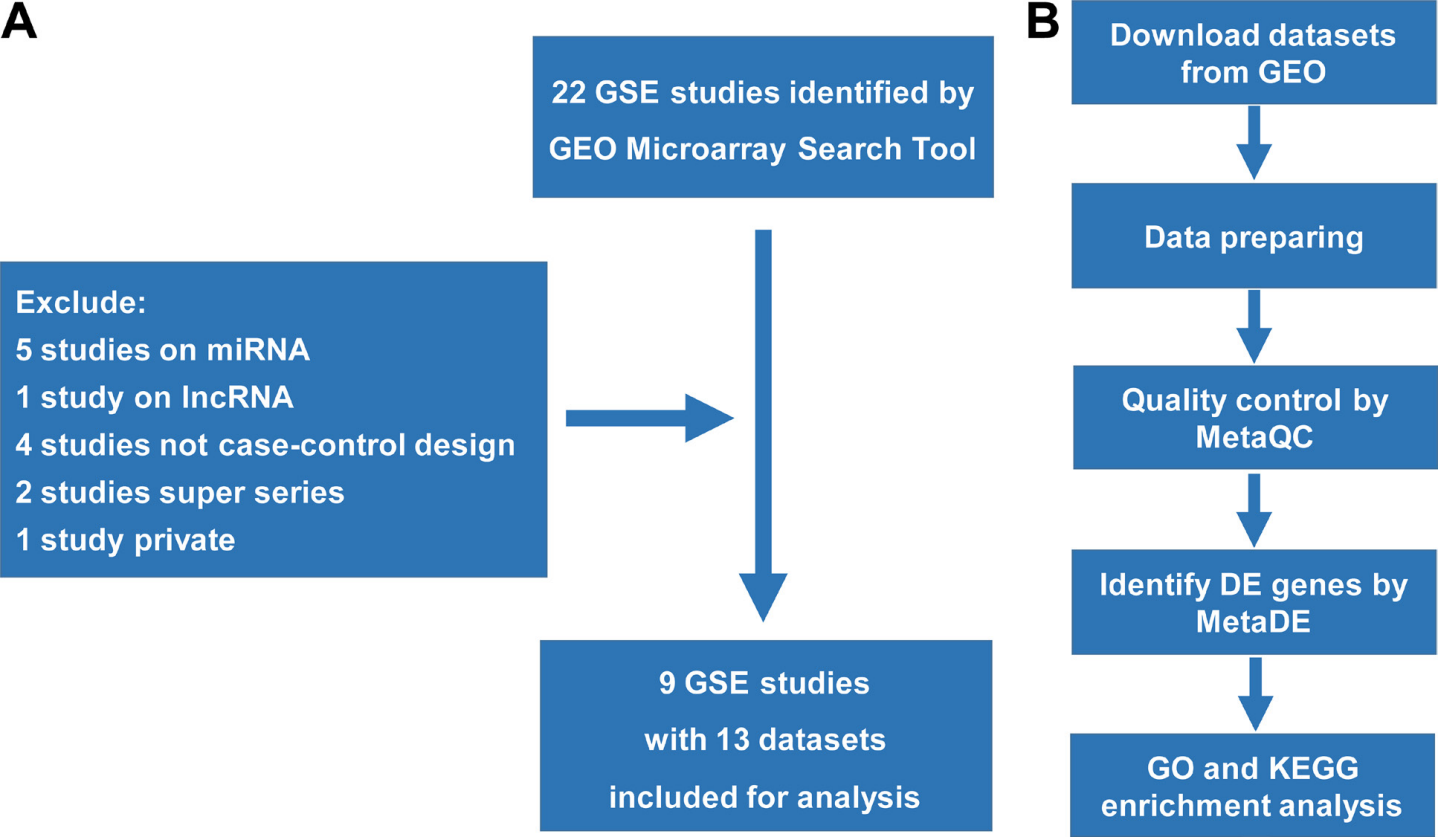
The process of data collection, selection, processing, and analysis (**A**) The process of data collection and selection; (**B**) The process of data processing and further analyses.

Nine well-designed GEO studies (accession numbers: GSE1615, GSE5850, GSE5090, GSE6798, GSE8157, GSE10946, GSE34526, GSE43264 and GSE48301) with PCOS patients and normal controls were included in this analysis [16–22]. Five different microarray platforms (GPL96, GPL 97, GPL570, GPL15362 and GPL6244) were used to generate 13 datasets from the nine case-control studies. Detailed descriptions of each dataset were shown in Table 1. After dataset preparing as shown in Figure 1, expression profiles for a total of 8470 genes in these datasets were extracted for further analysis.

**Table 1:** Characteristics of the 13 datasets included in the analysis

ID	GSE Acc	GPL	Details	#Control	#Case	Country	PMID	#Probe	#Symbol
1	GSE1615	GPL96/97	Theca cell	4	5	USA	15598877	44928	19923
2	GSE5090	GPL96	Omental adipose	8	9	Spain	17062763	22283	13236
3	GSE5850	GPL570	Oocytes MII	6	6	USA	17148555	54675	22878
4	GSE6798	GPL570	Vastus lateralis muscle	13	16	Denmark	17563058	54675	22878
5	GSE8157	GPL570	Vastus lateralis muscle	13	10	Denmark	18560589	54675	22878
6	GSE10946_lean	GPL570	Cumulus cells(lean)	6	5	Canada	19141487	54675	22878
7	GSE10946_obese	GPL570	Cumulus cells(obese)	5	7	Canada	19141487	54675	22878
8	GSE34526	GPL570	Granulosa cells	3	7	India	22904171	54675	22878
9	GSE43264	GPL15362	Subcutaneous adipose	7	8	Ireland	unpub	17126	16910
10	GSE48301_eEN	GPL6244	Endothelial cells	4	3	USA	23824412	32321	19740
11	GSE48301_eEP	GPL6244	Epithelial cells	3	4	USA	23824412	32321	19740
12	GSE48301_eMSC	GPL6244	Mesenchymal cells	4	3	USA	23824412	32321	19740
13	GSE48301_eSF	GPL6244	Stromal fibroblasts cells	4	4	USA	23824412	32321	19740

### Correlation of gene expression in various PCOS profiles

**Figure 2:**
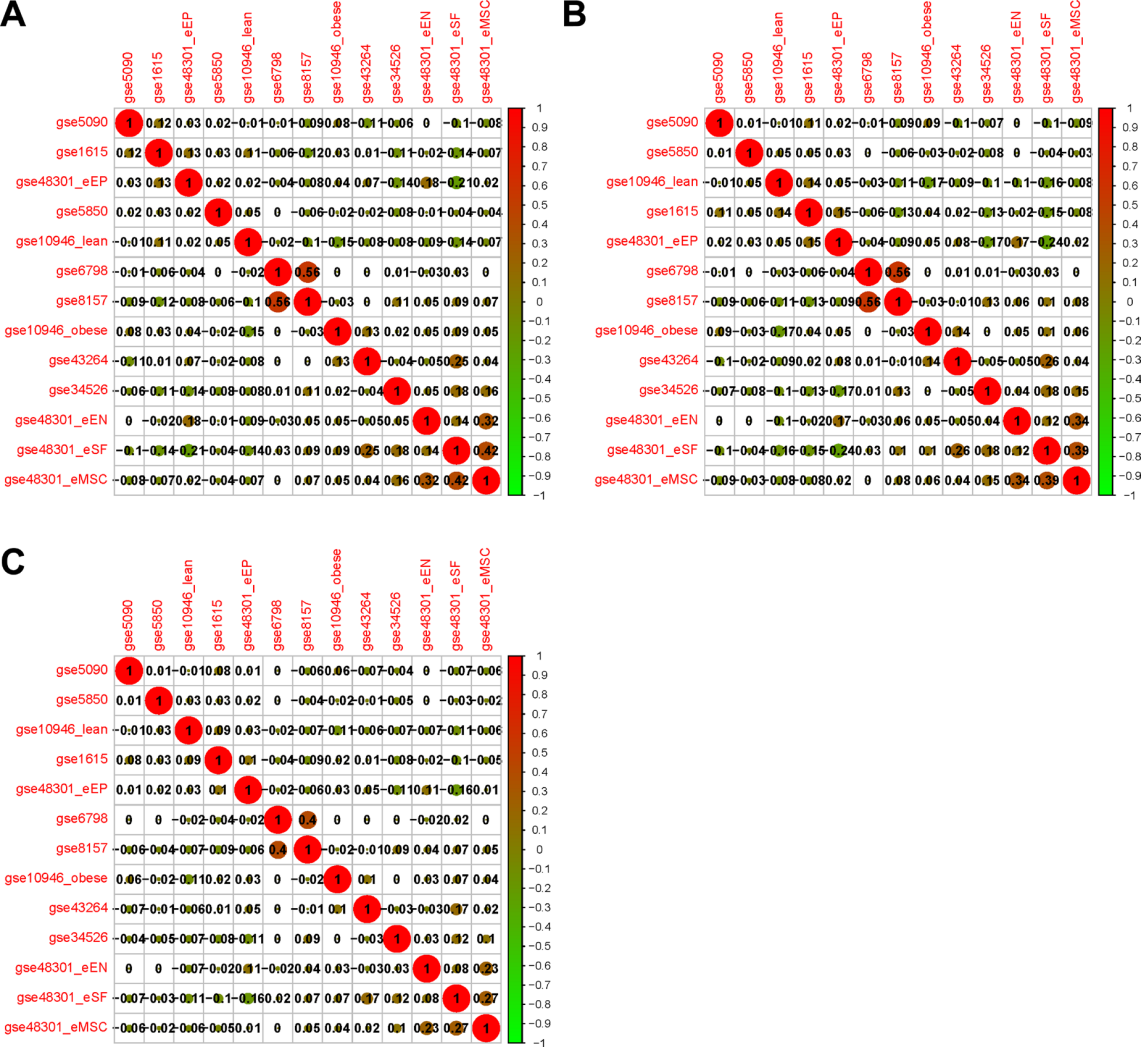
Correlation analyses of the relationships among 13 datasets based on gene expression variation profiles (**A**) Clustering with Pearson; (**B**) Clustering with Spearman; (**C**) Clustering with Kendall correlation coefficience. The positive and negative correlations between pairs of datasets are shown as red and green respectively, and the size of node index the strength of correlation.

The expressing pattern for each dataset was obtained by the methods of calculating the expression variation score (EVS) [23]. By clustering with Pearson, Spearman and Kendall correlations, the relationships for the 13 datasets were shown in Figure 2A, 2B and 2C, respectively. The coefficience values suggested that two datasets (GSE6798 and GSE8157) from PCOS muscles [20, 21] were extremely consistent with each other and obviously apart from the other 11 datasets. The high consistency may be resulting from the specificity of muscle tissues and the same laboratory. Thus, we integrated these two datasets as Muscle2 and performed further analysis. Among the remained 11 PCOS datasets (Figure 2A–2C), there only three datasets from GSE48301 showed reasonable similarities. As heterogeneity of sampling tissues and low quality of datasets, the quality control should be performed before meta-analysis.

### Quality control (QC) assessment in 11 datasets

**Figure 3:**
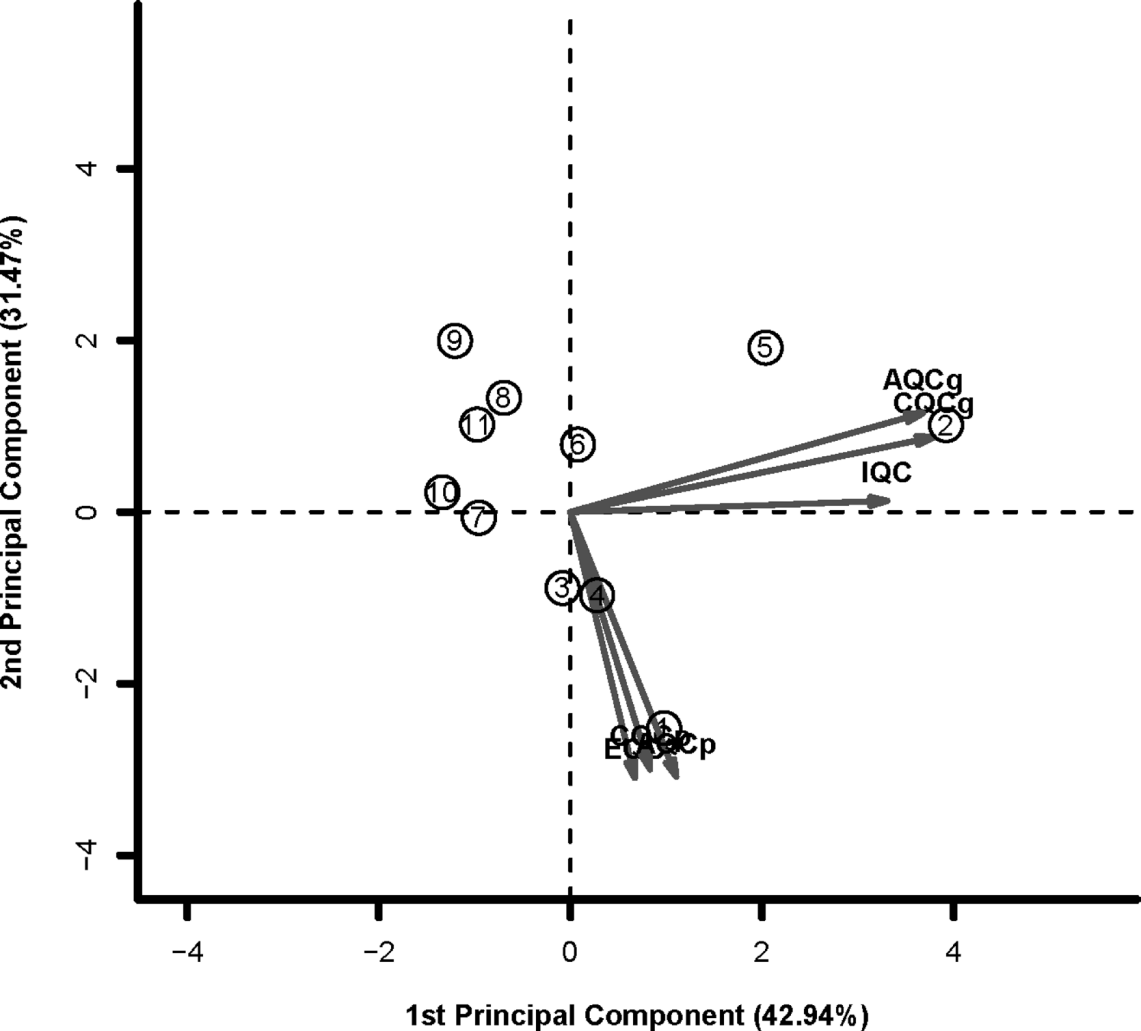
The PCA biplots of QC measures by MetaQC in 11 datasets The top five datasets (GSE43264, GSE48301_eSF, GSE1615, GSE10946_obese and GSE48301_eMSC) performed relatively well in most criteria. The bottom five datasets (GSE34526, GSE48301_eEN, GSE5090, GSE5850 and GSE10946_lean) were defined as exclusion cases. GSE48301_eEP was defined as borderline case.

To identify datasets with high quality and consistency, we carried out the quality assessment for the 11 PCOS datasets by the R package MetaQC [24]. Six QC assessments including homogeneity of coexpression structure, accuracy and consistency of biomarkers detection with or without pathway information were calculated. As shown in Table 2 and Figure 3 with PCA biplots, the top five datasets (GSE43264, GSE48301_eSF, GSE1615, GSE10946_obese and GSE48301_eMSC) performed relatively well in most criteria, while GSE48301_eEP as borderline case. After excluded the bottom five datasets (GSE34526, GSE48301_eEN, GSE5090, GSE5850 and GSE10946_lean) with low quality assessment, there were 6 datasets, named as PCOS6, remained for the following analysis.

**Table 2:** Results of quality control measures and SMRs for 11 datasets

ID	Study	IQC	EQC	CQCg	CQCp	AQCg	AQCp	SMR	Quality
1	GSE43264	4.02	4	2.51	3.32	2.63	4.54	2.67	high
2	GSE48301_eSF	9.49	2.82	8.89	2.44	12.97	1.17*	2.83	high
3	GSE1615	2.43	3	1.92*	2.64	0.92*	2.78	4.67	high
4	GSE10946_obese	7.45	4	0.27*	2.89	0.1*	0.7*	5.17	high
5	GSE48301_eMSC	6.1	2.62	7.12	0.59*	8.35	0.49*	5.33	high
6	GSE48301_eEP	6.25	3.1	1.88*	0.23*	0.46*	1.16*	5.5	borderline
7	GSE34526	1.74*	4	0.35*	1.36*	0.14*	0.05*	7.17	low
8	GSE48301_eEN	4.17	2.34*	0.46*	1.06*	0.63*	0.08*	7.17	low
9	GSE5090	0.98*	1.68*	0.51*	0.77*	1.86*	0.05*	8.33	low
10	GSE5850	0*	2.32*	0.05*	3.07	0.07*	0.41*	8.5	low
11	GSE10946_lean	3.15	2.8	0*	0.03*	0.05*	0.99*	8.67	low

* *P* value not significant after Bonferroni correction (i.e. *P* > 0.05/# of studies).

### Identification of differentially expressed genes (DEG) by MetaDE

**Figure 4:**
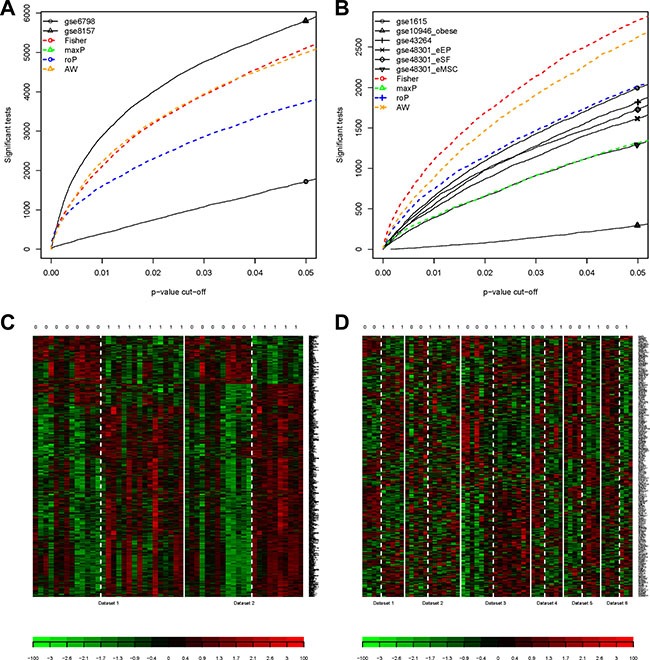
Results of DEGs by metaDE (**A**) The detection competency curves for Muscle2; (**B**) The detection competency curves for PCOS6; (**C**) The heatmap plot of DEG expression profiles for Muscle2; (**D**) The heatmap plot of DEG expression profiles for PCOS6. The column with 0 on top stands for normal samples, while 1 stands for PCOS patients.

Further analyses for both groups of Muscle2 and PCOS6 were performed by the MetaDE package. The datasets in two groups were re-merged and filtered, respectively. From the detection competency curves (Figure 4A, 4B), most methods of meta-analysis were useful to detected common DEGs among different datasets, especially for the Fisher method. Under the criteria of q value less than 0.05, there were 869 and 287 DEGs were identified in Muscle2 and PCOS6, respectively (Supplementary Table S1, S2). The expression patterns of these DEGs were shown in the heatmaps (Figure 4C, 4D), suggesting that the common DEGs were almost consistency in each dataset.

### Functional enrichment and signaling pathway analyses

**Figure 5:**
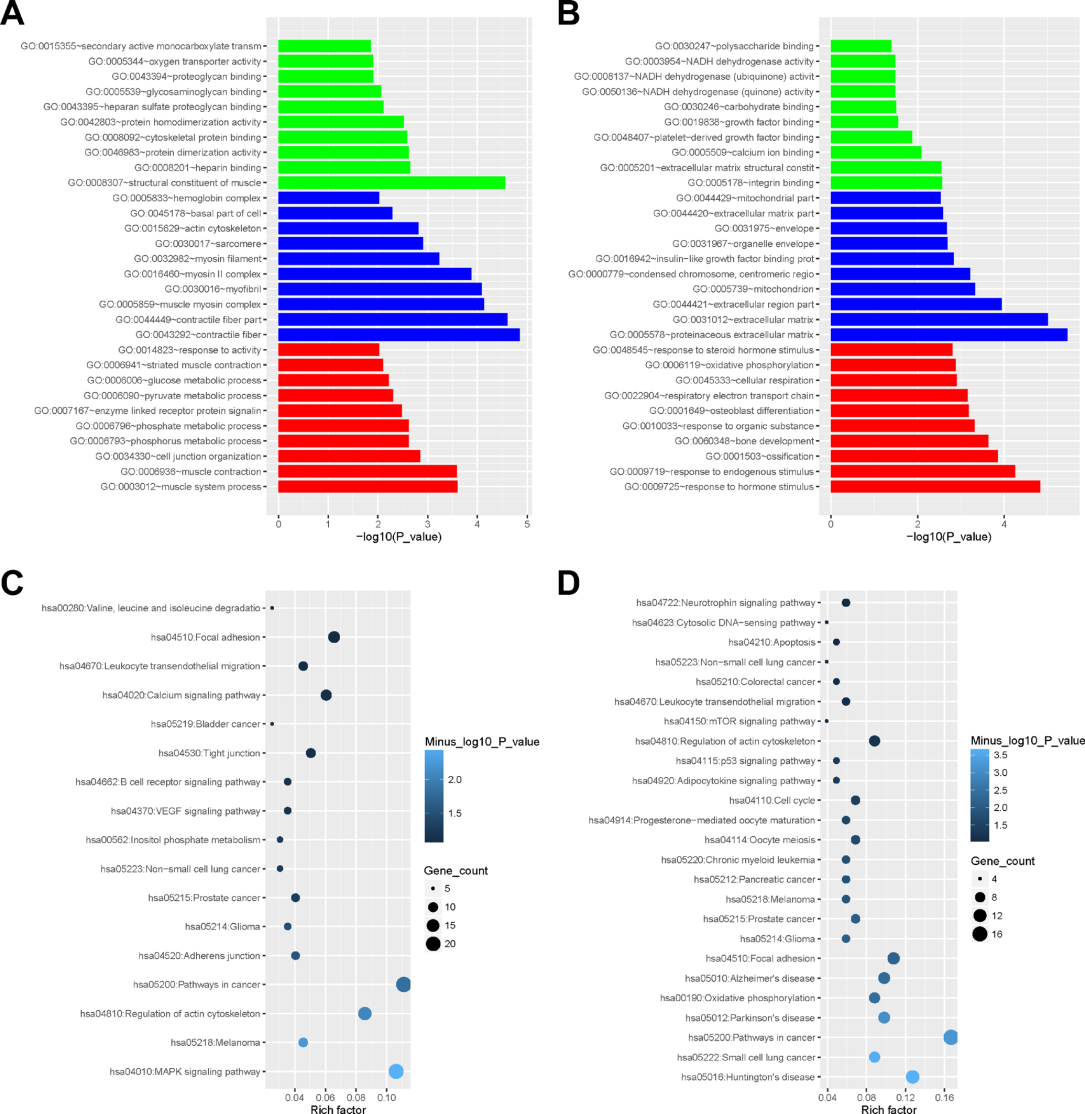
The enrichment analysis of the DEGs by DAVID (**A**) The barplot of gene ontology enrichment for Muscle2 (green for molecular function, blue for cellular component, red for biological process); (**B**) The barplot of gene ontology enrichment for PCOS6; (**C**) Rich factor plot of pathway enrichment for Muscle2; (**D**) Rich factor plot of pathway enrichment for PCOS6.

Functional enrichment analyses were performed with the DAVID web server. We found that the 869 DEGs in Muscle2 datasets were particularly enriched in the muscle system, metabolic process and activity (Figure 5A, Supplementary Table S3), agreed with previous reports [20, 21]. Meanwhile, the 287 DEGs in PCOS6 datasets mainly focused on the response to stimulus from endogenous, hormone and steroid hormone (Figure 5B, Supplementary Table S4).

Further signaling pathway analyses showed that the DEGs in Muscle2 almost enriched in several types of cancers, junction pathway, and signaling pathways including B cell receptor, VEGF, Calcium and MAPK (Figure 5C, Supplementary Table S5). Then, the PCOS6 DEGs directly focused on the oocyte meiosis and oocyte maturation, and also enriched in cancer pathway, apoptosis, adhesion, adipocytokine, neurotrophin, mTOR and p53 signaling pathways (Figure 5D, Supplementary Table S6).

### Further analyses of common and potential DEGs in PCOS

**Figure 6:**
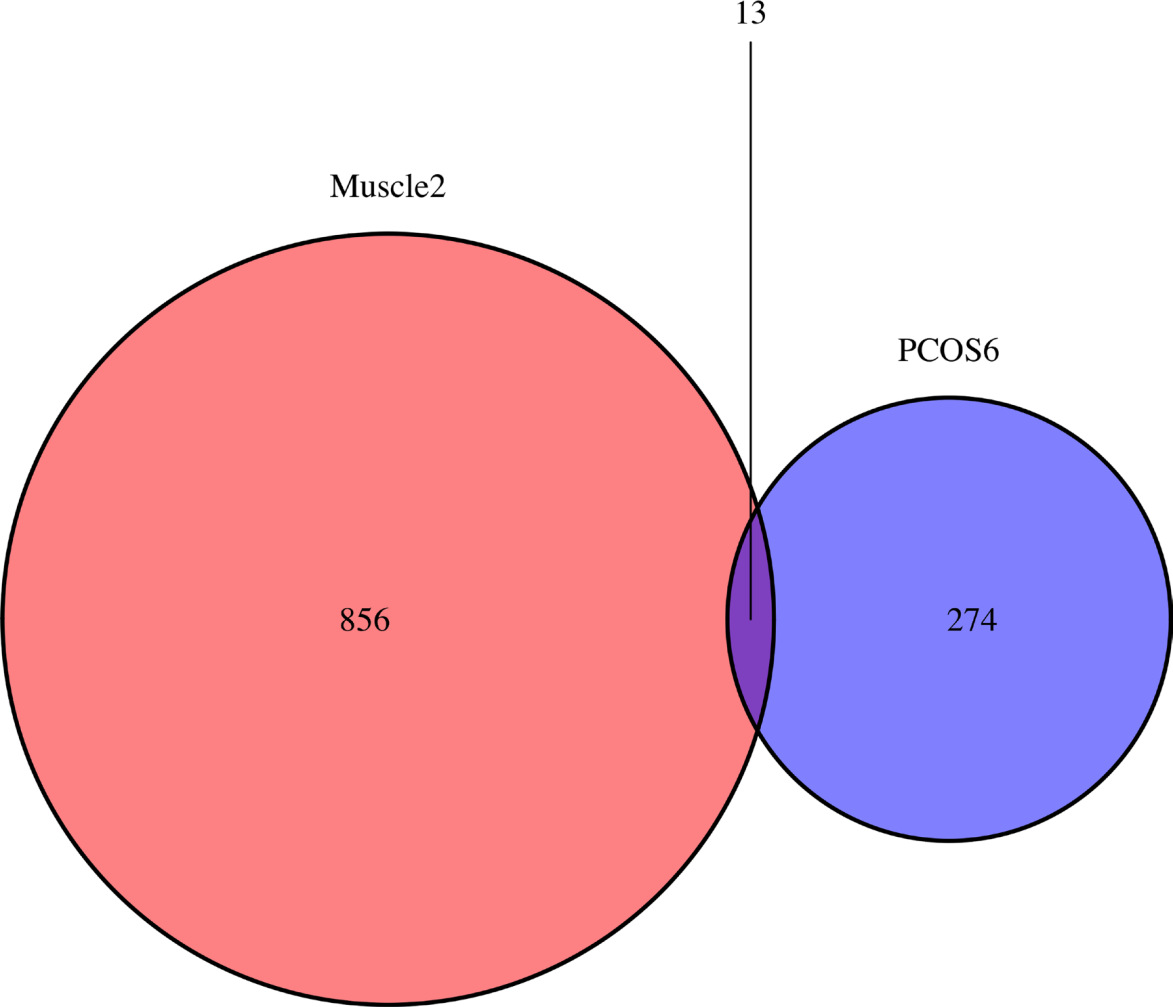
The venn plot for common biomarkers among DEGs of Muscle2 and PCOS6 The 856 DEGs are specific in Muscle2, while 274 DEGs are specific in PCOS6. There are 13 DEGs identified in both groups are SIAE, S100A8, ICAM1, EIF4E2, RAB32, FN1, MORC4, RGS10, SLC1A1, FGF7, SLC35D2, PDGFRA and APCDD1.

The DEGs overlapped in Muscle2 and PCOS6 groups were shown in a Venn diagram (Figure 6). Interestingly, there were 13 DEGs (SIAE, S100A8, ICAM1, EIF4E2, RAB32, FN1, MORC4, RGS10, SLC1A1, FGF7, SLC35D2, PDGFRA and APCDD1) identified in both groups. Then, these DEGs were classified as common markers of PCOS (Table 3).

**Table 3:** The 13 common DEGs identified in both Muscle2 and PCOS6

Gene	Muscle2			PCOS6		
	Meta.stat	Meta.p_value	Meta.q_value	Meta.stat	Meta.p_value	Meta.q_value
SIAE	24.8724	7.09E-05	0.0094	36.5715	0.0006	0.0383
S100A8	17.3211	0.0022	0.0485	36.2393	0.0006	0.0404
ICAM1	20.0402	0.0007	0.0290	49.7231	8.01E-06	0.0150
EIF4E2	32.4194	2.34E-06	0.0012	39.4171	0.0002	0.0324
RAB32	18.2467	0.0015	0.0410	40.2864	0.0002	0.0298
FN1	22.7958	0.0002	0.0172	43.3634	5.93E-05	0.0208
MORC4	21.3159	0.0004	0.0236	34.4672	0.0011	0.0469
RGS10	19.1576	0.0010	0.0343	47.2925	1.87E-05	0.0156
SLC1A1	18.3162	0.0014	0.0403	38.6278	0.0003	0.0333
FGF7	19.3963	0.0009	0.0328	34.6910	0.00103	0.0460
SLC35D2	19.9783	0.0007	0.0292	38.0259	0.0004	0.0347
PDGFRA	18.5395	0.0013	0.0382	38.8920	0.0003	0.0333
APCDD1	27.6092	1.82E-05	0.0040	35.3975	0.0008	0.0430

Furthermore, the multivariate Cox proportional hazard analyses of the 13 DEGs among patients with ovarian serous cystadenocarcinoma (TCGA: http://cancergenome.nih.gov) were performed. We found that four genes (SIAE, ICAM1, FN1, and FGF7) were significantly correlated with disease-free survival, while the other three genes (SIAE, FGF7, and PDGFRA) were significantly correlated with overall survival (Table 4). These findings further confirmed that the critical role of these candidate genes in PCOS.

**Table 4:** Cox proportional hazard analysis of the 13 common DEGs for DFS and OS among patients with ovarian serous cystadenocarcinoma

Variable	PHA Test*	HR	*P*	95%CI_L	95%CI_H
**DFS**					
SIAE	0.7022	1.1439	0.0354†	1.0092	1.2965
ICAM1	0.9702	1.2007	0.0361†	1.0119	1.4247
EIF4E2	0.7475	1.2159	0.0707	0.9837	1.5030
RAB32	0.1007	0.7993	0.0586	0.6336	1.0082
FN1	0.4617	0.7808	0.0158†	0.6387	0.9547
FGF7	0.3707	1.4987	0.0001†	1.2204	1.8404
**OS**					
AGE	0.8117	1.0325	0.0008†	1.0134	1.0520
FGF7	0.1797	1.4019	0.0129†	1.0741	1.8298
PDGFRA	0.2893	0.5232	0.0052†	0.3321	0.8243

Abbreviations: DFS, disease-free survival; OS, overall survival; PHA test, proportional hazards assumption test; HR, hazard ratio.

* _!_PHA test *P* < 0.05 violates the hazards assumption.

† Significant.

## DISCUSSION

PCOS is a highly complex endocrine disorder and affected by phenotypically heterogeneous. As its phenotypic heterogeneity and studies with small sample sizes, the power is low to identify specific genes for PCOS. To increase the sample size and make powerful analysis, we performed the integrated and meta-analyses to improve the quality of gene association studies. Then, we identified 13 genetic markers may be potential molecular factors for the diagnosis and prognosis of PCOS patients.

Recently, microarray analysis of gene expression profiles has been widely used to identify genes and biological pathways associated with various complex diseases, including PCOS. However, previous studies have sampled different tissues from PCOS patients. The pathological factors and mechanisms in various tissues of PCOS patients may be similar. In this study, to identify the common PCOS-associated genes in multiple types of tissues, we carried out the integrated analysis and identified 13 common DEGs for PCOS. Taking ICAM-1 for an example, two SNPs of ICAM-1, G241R and K469E, have been found to be risk factors for PCOS [25]. Moreover, ICAM1 K469E is associated with obesity and PCOS, according to serum triglyceride levels [26]. As evidenced by gene ontology and pathway analyses, these DEGs in Muscle2 datasets directly involved in muscle mysion complex, myofibril, muscle system and muscle contraction. The DEGs in PCOS6 datasets mainly focused on oocyte pathways, including oocyte meiosis (PPP2R1B, RPS6KA6, MAD2L1, SGOL1, CAMK2D, IGF1, and ANAPC11) and progesterone-mediated oocyte maturation (RPS6KA6, MAD2L1, IGF1, ANAPC11, PIK3R3, and CCNA2). In-depth functional studies of these candidate genes and signaling pathways may improve the understanding of PCOS.

Along with next generation sequencing (NGS), increasing numbers of studies explored differences in ncRNA and epigenetic modifications (such as DNA methylation) between PCOS patients and normal controls. It has been reported that serum miR-21 is markedly increased in PCOS patients. Through targeting LATS1, miR-21 may promote PCOS progression and act as a novel non-invasive biomarker for the diagnosis of PCOS [27]. lncRNA SRA has been found to be associated with PCOS, and may be an important mediator of adiposity-related processes in individual susceptible to PCOS [28]. The global methylation of peripheral leukocyte DNA has no difference between PCOS patients and controls [29]. The methods for genome-wide DNA methylation analysis at a single base pair resolution are evolving quickly [30]. Therefore, it will be more feasible to carry out genome-wide studies with sufficient samples in different tissue types.

In conclusion, this meta-analysis successfully integrated gene expression datasets for PCOS. This process stabilized the effects of the studies' extreme heterogeneity and small sample sizes, and uncovered genes and biological pathways associated with PCOS. Finally, we identified 13 common genes among various PCOS tissues, providing novel and potential molecular markers for the diagnosis and prognosis of PCOS diseases. Further functional studies on these genes may improve understanding of the pathological processes of PCOS.

## MATERIALS AND METHODS

### Collection and inclusion criteria of studies

Using the Gene Expression Omnibus (GEO) Microarray Search Tool (http://gbnci.abcc.ncifcrf.gov/geo/) [31], we searched the GEO database for publicly available studies from its inception to August 31, 2016, using the following keywords: “Homo sapiens” (organism), “PCOS” or “Polycystic ovary syndrome” (study keywords), and “RNA” (sample type). After a systematic review, 22 GSE studies were collected. The inclusion criteria for studies were as follows: 1) patients diagnosed with PCOS diseases and normal controls; 2) gene expression profiling of mRNA; and 3) sufficient information to perform the analysis. Then, 13 datasets from 9 studies were collected for subsequent analysis (Table 1). Figure 1A provides details of the process of data collection and study selection. The workflow of data processing and analysis is illustrated in Figure 1B.

### Dataset preparing

Thirteen gene expression datasets were downloaded from the GEO database, which were completed in five different platforms. Because different probes were used to detect gene expression in different platforms, the number of detectable genes varied across platforms (Table 1). To merge these datasets from different studies, we used three functions in the R package MetaDE: MetaDE.match, MetaDE.merge, and MetaDE.filtering [15]. First, the probe with largest interquartile range (IQR) among all probes annotated to the same gene was selected to represent the expression level of the gene. Second, we merged the gene expression profiles to maintain the commonly profiled genes across the 13 datasets. Finally, either un-expressed (10% genes with small mean intensity) or un-informative genes (10% small standard deviation) were filtered, and the remaining 8470 genes were retained for further analysis.

### Statistical analyses

The ind.analysis function in the MetaDE package was used to compare gene expression between PCOS patients and normal controls in each dataset. We used moderated t statistics to evaluate statistical significance.

The transcriptome of each dataset was evaluated with the expression variation score (EVS), introduced by Marina Sirota [23]. In each dataset, the EVS for the gene *j* is defined as EVS_j_=-sign(t_j_)log(p_j_), where t_j_ is the moderated t statistic for the gene *j* and p_j_ is the *p-value* of the test. The scale and sign of the EVS represent the degree of significance and the ‘direction’ of the association, respectively. Then, we used the EVS vector to represent the pattern of the gene expression profile for each dataset. The consistency of the transcriptome tendency was estimated by the correlation coefficients of the EVS vectors between datasets [23].

### Quality control

Before meta-analysis, the MetaQC package [24] was used to determine the inclusion/exclusion criteria for meta-analysis. The six quantitative quality control (QC) measures were calculated by MetaQC, and the principal component analysis (PCA) biplots and standardized mean ranks (SMR) were helpful to identify and exclude study with low quality before further meta-analysis.

### Meta-analysis

The meta-analysis in each group was completed by MetaDE package. The *q*-values less than 0.05 for Fisher method, which were subjected to multiple-testing correction with the Benjamini-Hochberg method [32], were the critical to DEGs. The expression patterns in datasets of all DEGs were shown on heatmap plot.

### Gene ontology and pathway enrichment analyses

The gene ontology and pathway enrichment analyses of interested gene sets were completed by DAVID web servers (https://david.ncifcrf.gov) [33].

### TCGA and survival analysis

A study on ovarian serous cystadenocarcinoma (ov_tcga) with 586 patients was collected form TCGA database (TCGA: http://cancergenome.nih.gov). Based on information of disease-free survival (DFS) and overall survival (OS) for ov patients, we used a multivariate Cox proportional hazards model to determine hazard ratios (HRs) for biomarkers. All analyses were performed with R statistical software (survival and risksetROC packages).

## SUPPLEMENTARY MATERIALS TABLES




