# Supplementary material for: Integrated analyses for genetic markers of polycystic ovary syndrome with 9 case-control studies of gene expression profiles
Source: Oncotarget. 2016 Dec 10;8(2):3170–80. doi: 10.18632/oncotarget.13881 (PMC5356873; doi:10.18632/oncotarget.13881)
Supplement: Supplementary file 1 [file oncotarget-08-3170-s001.pdf]

Integrated analyses for genetic markers of polycystic ovary syndrome with 9 case-control studies of gene expression profiles

Supplementary Materials

Supplementary Table S1: The 869 DEGs identified in two datasets from Muscle2. See Supplementary_Table_S1

Supplementary Table S2: The 287 DEGs identified in 6 datasets from PCOS6. See Supplementary_Table_S2

Supplementary Table S3: The result of DAVID gene ontology (GO) enrichment for 869 DEGs from Muscle2. See Supplementary_Table_S3

Supplementary Table S4: The result of DAVID GO enrichment for 287 DEGs from PCOS6. See Supplementary_Table_S4

Supplementary Table S5: The result of DAVID pathway enrichment for 869 DEGs from Muscle2. See Supplementary_Table_S5

Supplementary Table S6: The result of DAVID pathway enrichment for 287 DEGs from PCOS6. See Supplementary_Table_S6
