# Supplementary material for: Integrated analyses for genetic markers of polycystic ovary syndrome with 9 case-control studies of gene expression profiles
Source: Oncotarget. 2016 Dec 10;8(2):3170–80. doi: 10.18632/oncotarget.13881 (PMC5356873; doi:10.18632/oncotarget.13881)
Supplement: Supplementary file 2 [file oncotarget-08-3170-s002.doc]

**Supplemental Data**

**Table S1. The 869 DEGs identified in two datasets from Muscle2.**

| **Muscle2** | **Fisher** | | | **gse6798** | | **gse8157** | |
| --- | --- | --- | --- | --- | --- | --- | --- |
| gene | meta.stastic | meta.p_value | meta.q_value | ind.statistic | ind.p_value | ind.statistic | ind.p_value |
| NANOS1 | 42.23 | 1.00E-20 | 3.71E-17 | -3.09 | 2.70E-06 | -2.28 | 2.51E-04 |
| TNPO2 | 47.07 | 1.00E-20 | 3.71E-17 | -2.59 | 3.71E-05 | -3.17 | 1.62E-06 |
| VSIG1 | 48.79 | 1.00E-20 | 3.71E-17 | -3.06 | 2.88E-06 | -2.86 | 8.81E-06 |
| SNAPC5 | 103.33 | 1.00E-20 | 3.71E-17 | -3.78 | 1.00E-20 | -1.71 | 3.66E-03 |
| C2ORF61 | 41.63 | 1.00E-20 | 3.71E-17 | -2.46 | 7.23E-05 | -2.80 | 1.26E-05 |
| LDHB | 39.15 | 1.80E-07 | 4.17E-04 | 2.87 | 7.74E-06 | 2.19 | 4.07E-04 |
| MYH4 | 39.40 | 1.80E-07 | 4.17E-04 | -2.80 | 1.01E-05 | -2.26 | 2.76E-04 |
| MIR6787 | 38.84 | 1.80E-07 | 4.17E-04 | -2.19 | 3.36E-04 | -2.83 | 1.10E-05 |
| RP11-480A16.1 | 36.02 | 3.60E-07 | 4.76E-04 | -2.63 | 3.08E-05 | -2.15 | 4.91E-04 |
| FRZB | 37.13 | 3.60E-07 | 4.76E-04 | -2.54 | 4.59E-05 | -2.33 | 1.88E-04 |
| PAX8 | 37.02 | 3.60E-07 | 4.76E-04 | -2.35 | 1.35E-04 | -2.52 | 6.76E-05 |
| RHOB | 36.57 | 3.60E-07 | 4.76E-04 | -2.21 | 2.94E-04 | -2.61 | 3.90E-05 |
| SLC38A1 | 36.35 | 3.60E-07 | 4.76E-04 | 2.60 | 3.58E-05 | 2.21 | 3.57E-04 |
| C9ORF135 | 36.04 | 3.60E-07 | 4.76E-04 | 2.14 | 4.35E-04 | 2.64 | 3.44E-05 |
| MAD1L1 | 35.45 | 7.20E-07 | 8.89E-04 | -2.17 | 3.69E-04 | -2.55 | 5.45E-05 |
| ALPK2 | 33.90 | 1.44E-06 | 9.52E-04 | 2.68 | 2.30E-05 | 1.86 | 1.89E-03 |
| TECRL | 33.69 | 1.44E-06 | 9.52E-04 | 3.00 | 3.96E-06 | 1.41 | 1.22E-02 |
| MYL10 | 32.93 | 1.44E-06 | 9.52E-04 | -2.34 | 1.40E-04 | -2.15 | 5.07E-04 |
| LAMB2P1 | 34.58 | 1.26E-06 | 9.52E-04 | -2.05 | 7.11E-04 | -2.59 | 4.35E-05 |
| MOBP | 34.29 | 1.26E-06 | 9.52E-04 | -2.30 | 1.75E-04 | -2.32 | 2.05E-04 |
| IRX3 | 34.49 | 1.26E-06 | 9.52E-04 | -2.23 | 2.66E-04 | -2.41 | 1.22E-04 |
| CCDC57 | 34.24 | 1.26E-06 | 9.52E-04 | -1.81 | 2.19E-03 | -2.75 | 1.67E-05 |
| CYP2A7P1 | 32.94 | 1.44E-06 | 9.52E-04 | -1.50 | 8.31E-03 | -2.87 | 8.45E-06 |
| AMPD3 | 33.06 | 1.44E-06 | 9.52E-04 | 2.14 | 4.27E-04 | -2.37 | 1.55E-04 |
| HS6ST2 | 33.46 | 1.44E-06 | 9.52E-04 | -1.90 | 1.44E-03 | -2.62 | 3.76E-05 |
| MSTN | 34.16 | 1.26E-06 | 9.52E-04 | -2.72 | 1.78E-05 | -1.83 | 2.15E-03 |
| NBPF3 | 33.54 | 1.44E-06 | 9.52E-04 | -1.92 | 1.30E-03 | -2.60 | 3.99E-05 |
| RASSF3 | 35.10 | 8.99E-07 | 9.52E-04 | -2.93 | 5.76E-06 | -1.67 | 4.15E-03 |
| H19 | 31.93 | 2.34E-06 | 1.24E-03 | 2.11 | 5.02E-04 | 2.29 | 2.32E-04 |
| GPD1 | 32.64 | 2.16E-06 | 1.24E-03 | -2.21 | 2.87E-04 | -2.25 | 2.84E-04 |
| DDX49 | 32.60 | 2.16E-06 | 1.24E-03 | -1.92 | 1.30E-03 | -2.52 | 6.40E-05 |
| EIF4E2 | 32.42 | 2.34E-06 | 1.24E-03 | -2.61 | 3.33E-05 | -1.77 | 2.74E-03 |
| UCK1 | 32.17 | 2.34E-06 | 1.24E-03 | -2.23 | 2.67E-04 | -2.20 | 3.88E-04 |
| LMO7DN | 32.62 | 2.16E-06 | 1.24E-03 | -2.28 | 2.04E-04 | -2.19 | 4.03E-04 |
| GPR68 | 32.08 | 2.34E-06 | 1.24E-03 | -1.49 | 8.58E-03 | -2.80 | 1.26E-05 |
| ARHGEF34P | 31.74 | 2.70E-06 | 1.39E-03 | -1.78 | 2.47E-03 | -2.55 | 5.18E-05 |
| CDKN1C | 31.11 | 3.24E-06 | 1.47E-03 | 1.96 | 1.05E-03 | 2.35 | 1.68E-04 |
| GLG1 | 30.68 | 3.42E-06 | 1.47E-03 | -2.21 | 2.86E-04 | -2.06 | 7.59E-04 |
| ATP11A-AS1 | 30.75 | 3.42E-06 | 1.47E-03 | -1.92 | 1.31E-03 | -2.36 | 1.60E-04 |
| IPO4 | 31.17 | 3.24E-06 | 1.47E-03 | -1.53 | 7.22E-03 | -2.69 | 2.36E-05 |
| ACY3 | 31.09 | 3.24E-06 | 1.47E-03 | -1.92 | 1.29E-03 | -2.39 | 1.37E-04 |
| VN1R5 | 30.64 | 3.42E-06 | 1.47E-03 | -2.21 | 2.91E-04 | -2.06 | 7.61E-04 |
| PPP1R2P9 | 31.16 | 3.24E-06 | 1.47E-03 | -1.25 | 2.26E-02 | -2.92 | 7.56E-06 |
| RBM3 | 30.61 | 3.60E-06 | 1.52E-03 | -2.44 | 8.11E-05 | -1.77 | 2.78E-03 |
| OSBPL7 | 30.38 | 5.04E-06 | 2.07E-03 | -2.30 | 1.75E-04 | -1.92 | 1.44E-03 |
| YTHDC2 | 30.24 | 5.94E-06 | 2.39E-03 | -2.26 | 2.17E-04 | -1.95 | 1.26E-03 |
| RP11-90P13.1 | 29.72 | 7.02E-06 | 2.60E-03 | -2.33 | 1.55E-04 | -1.82 | 2.28E-03 |
| RP1-39G22.7 | 29.72 | 7.02E-06 | 2.60E-03 | -1.27 | 2.08E-02 | -2.75 | 1.69E-05 |
| CLDN15 | 29.93 | 6.84E-06 | 2.60E-03 | -1.85 | 1.81E-03 | -2.35 | 1.75E-04 |
| RIMKLA | 29.67 | 7.02E-06 | 2.60E-03 | -2.28 | 2.05E-04 | -1.88 | 1.76E-03 |
| KRBOX1 | 29.54 | 7.20E-06 | 2.61E-03 | -1.69 | 3.69E-03 | -2.44 | 1.05E-04 |
| MSS51 | 29.26 | 8.63E-06 | 2.82E-03 | -2.31 | 1.64E-04 | -1.78 | 2.70E-03 |
| KCTD7 | 29.14 | 8.81E-06 | 2.82E-03 | -2.19 | 3.34E-04 | -1.93 | 1.41E-03 |
| CAMSAP2 | 29.11 | 8.81E-06 | 2.82E-03 | 2.27 | 2.11E-04 | 1.82 | 2.26E-03 |
| NFYC-AS1 | 29.18 | 8.63E-06 | 2.82E-03 | -2.02 | 7.95E-04 | -2.12 | 5.79E-04 |
| ATP2B2 | 29.26 | 8.63E-06 | 2.82E-03 | -1.37 | 1.43E-02 | -2.65 | 3.11E-05 |
| SREBF1 | 29.20 | 8.63E-06 | 2.82E-03 | -1.25 | 2.26E-02 | -2.71 | 2.01E-05 |
| HIST1H2BC | 29.43 | 7.92E-06 | 2.82E-03 | -2.29 | 1.92E-04 | -1.84 | 2.11E-03 |
| RSPO3 | 28.94 | 9.89E-06 | 2.87E-03 | 2.51 | 5.52E-05 | 1.48 | 9.41E-03 |
| SLC29A1 | 28.63 | 1.01E-05 | 2.87E-03 | -2.06 | 6.54E-04 | -2.02 | 9.28E-04 |
| TPSG1 | 28.63 | 1.01E-05 | 2.87E-03 | -1.90 | 1.45E-03 | -2.18 | 4.18E-04 |
| CMTM6 | 28.90 | 9.89E-06 | 2.87E-03 | -1.73 | 3.13E-03 | -2.35 | 1.69E-04 |
| KIAA0825 | 28.95 | 9.89E-06 | 2.87E-03 | 2.13 | 4.68E-04 | 1.98 | 1.11E-03 |
| KLC3 | 29.01 | 9.35E-06 | 2.87E-03 | -1.68 | 3.86E-03 | -2.40 | 1.30E-04 |
| SH2D1B | 28.70 | 1.01E-05 | 2.87E-03 | -2.38 | 1.12E-04 | -1.62 | 5.25E-03 |
| MPDU1 | 28.59 | 1.08E-05 | 3.03E-03 | -1.40 | 1.22E-02 | -2.56 | 5.05E-05 |
| XPO4 | 28.38 | 1.24E-05 | 3.33E-03 | 2.10 | 5.50E-04 | 1.96 | 1.25E-03 |
| SHISA2 | 28.32 | 1.26E-05 | 3.33E-03 | -2.34 | 1.42E-04 | -1.63 | 4.99E-03 |
| ALDH6A1 | 28.40 | 1.24E-05 | 3.33E-03 | 2.15 | 4.03E-04 | 1.89 | 1.68E-03 |
| CAMKK2 | 28.30 | 1.28E-05 | 3.33E-03 | -2.34 | 1.44E-04 | -1.63 | 4.96E-03 |
| KCNK13 | 28.39 | 1.24E-05 | 3.33E-03 | -1.29 | 1.97E-02 | -2.64 | 3.47E-05 |
| C12ORF75 | 28.26 | 1.33E-05 | 3.43E-03 | 1.91 | 1.35E-03 | 2.13 | 5.41E-04 |
| EMC3 | 28.01 | 1.51E-05 | 3.68E-03 | 2.07 | 6.37E-04 | 1.95 | 1.30E-03 |
| SLC22A18AS | 27.89 | 1.55E-05 | 3.68E-03 | -1.72 | 3.15E-03 | -2.26 | 2.78E-04 |
| EEF1D | 27.91 | 1.55E-05 | 3.68E-03 | -2.06 | 6.74E-04 | -1.95 | 1.29E-03 |
| ZYX | 28.02 | 1.51E-05 | 3.68E-03 | -1.76 | 2.70E-03 | -2.24 | 3.05E-04 |
| HAO2 | 28.06 | 1.49E-05 | 3.68E-03 | -2.13 | 4.61E-04 | -1.88 | 1.75E-03 |
| RP11-310J24.3 | 27.99 | 1.53E-05 | 3.68E-03 | -2.06 | 6.65E-04 | -1.95 | 1.26E-03 |
| RNF40 | 27.79 | 1.62E-05 | 3.80E-03 | -1.67 | 3.97E-03 | -2.29 | 2.32E-04 |
| LOC654841 | 27.75 | 1.67E-05 | 3.87E-03 | -1.86 | 1.72E-03 | -2.13 | 5.50E-04 |
| TTLL9 | 27.72 | 1.69E-05 | 3.87E-03 | -1.20 | 2.76E-02 | -2.64 | 3.47E-05 |
| SRSF5 | 27.63 | 1.82E-05 | 4.01E-03 | -1.01 | 5.91E-02 | -2.75 | 1.69E-05 |
| APCDD1 | 27.61 | 1.82E-05 | 4.01E-03 | -0.90 | 8.92E-02 | -2.82 | 1.13E-05 |
| PDZRN3 | 27.67 | 1.78E-05 | 4.01E-03 | -1.45 | 1.01E-02 | -2.45 | 9.73E-05 |
| MLF1 | 27.46 | 1.91E-05 | 4.05E-03 | -2.27 | 2.11E-04 | -1.62 | 5.14E-03 |
| LPL | 27.47 | 1.91E-05 | 4.05E-03 | 2.02 | 7.97E-04 | 1.94 | 1.36E-03 |
| HIPK1 | 27.43 | 1.92E-05 | 4.05E-03 | -1.91 | 1.34E-03 | -2.05 | 8.30E-04 |
| NCAM1 | 27.52 | 1.87E-05 | 4.05E-03 | -1.68 | 3.80E-03 | -2.26 | 2.78E-04 |
| SLC37A4 | 27.36 | 1.98E-05 | 4.12E-03 | -1.93 | 1.21E-03 | -2.02 | 9.43E-04 |
| FAM120C | 27.25 | 2.12E-05 | 4.37E-03 | -1.67 | 4.02E-03 | -2.24 | 3.01E-04 |
| EPS8L2 | 27.13 | 2.32E-05 | 4.73E-03 | -0.65 | 2.10E-01 | -2.96 | 6.12E-06 |
| ATP11B | 26.98 | 2.52E-05 | 5.07E-03 | -0.78 | 1.37E-01 | -2.84 | 1.01E-05 |
| ASB2 | 26.93 | 2.61E-05 | 5.14E-03 | -1.71 | 3.39E-03 | -2.18 | 4.18E-04 |
| FRMD6 | 26.95 | 2.59E-05 | 5.14E-03 | -2.31 | 1.65E-04 | -1.50 | 8.49E-03 |
| TAPBPL | 26.83 | 2.70E-05 | 5.19E-03 | -1.97 | 1.03E-03 | -1.92 | 1.44E-03 |
| ARHGAP35 | 26.82 | 2.72E-05 | 5.19E-03 | -1.50 | 8.17E-03 | -2.34 | 1.84E-04 |
| DLGAP1-AS1 | 26.83 | 2.70E-05 | 5.19E-03 | -1.67 | 4.05E-03 | -2.21 | 3.68E-04 |
| MYO15B | 26.70 | 2.90E-05 | 5.38E-03 | -1.72 | 3.25E-03 | -2.15 | 4.90E-04 |
| OPN4 | 26.73 | 2.86E-05 | 5.38E-03 | -1.46 | 9.76E-03 | -2.36 | 1.61E-04 |
| EFHB | 26.69 | 2.93E-05 | 5.38E-03 | -1.64 | 4.59E-03 | -2.22 | 3.49E-04 |
| AL833181 | 26.68 | 2.93E-05 | 5.38E-03 | -1.35 | 1.49E-02 | -2.44 | 1.08E-04 |
| ATXN7L3B | 26.59 | 3.15E-05 | 5.66E-03 | -1.68 | 3.76E-03 | -2.17 | 4.48E-04 |
| RP11-495K9.3 | 26.59 | 3.15E-05 | 5.66E-03 | -1.83 | 1.96E-03 | -2.04 | 8.58E-04 |
| RP11-466P24.7 | 26.46 | 3.35E-05 | 5.96E-03 | -1.46 | 9.55E-03 | -2.33 | 1.88E-04 |
| ACTN3 | 26.34 | 3.47E-05 | 6.07E-03 | -1.55 | 6.52E-03 | -2.25 | 2.93E-04 |
| KRT6B | 26.31 | 3.47E-05 | 6.07E-03 | -1.41 | 1.17E-02 | -2.36 | 1.65E-04 |
| SLAMF7 | 26.21 | 3.69E-05 | 6.39E-03 | 1.60 | 5.31E-03 | 2.20 | 3.83E-04 |
| NMRK2 | 26.01 | 4.01E-05 | 6.46E-03 | -1.96 | 1.07E-03 | -1.84 | 2.11E-03 |
| LOC151121 | 26.09 | 3.90E-05 | 6.46E-03 | -2.35 | 1.33E-04 | -1.34 | 1.62E-02 |
| CYB561 | 26.15 | 3.78E-05 | 6.46E-03 | -1.63 | 4.66E-03 | -2.17 | 4.49E-04 |
| USP21 | 25.99 | 4.01E-05 | 6.46E-03 | -1.62 | 5.01E-03 | -2.16 | 4.53E-04 |
| CBWD1 | 26.02 | 4.01E-05 | 6.46E-03 | -1.89 | 1.50E-03 | -1.92 | 1.49E-03 |
| PPP4R1 | 26.05 | 3.94E-05 | 6.46E-03 | -1.37 | 1.42E-02 | -2.37 | 1.55E-04 |
| HBD | 26.04 | 3.94E-05 | 6.46E-03 | -2.19 | 3.29E-04 | -1.56 | 6.72E-03 |
| BC015159 | 26.11 | 3.89E-05 | 6.46E-03 | -1.84 | 1.93E-03 | -1.98 | 1.11E-03 |
| MOV10 | 25.96 | 4.14E-05 | 6.53E-03 | -1.84 | 1.90E-03 | -1.96 | 1.22E-03 |
| WNT4 | 25.95 | 4.14E-05 | 6.53E-03 | -1.73 | 3.06E-03 | -2.06 | 7.57E-04 |
| FAIM2 | 25.92 | 4.16E-05 | 6.53E-03 | -1.44 | 1.07E-02 | -2.30 | 2.21E-04 |
| HOXA10-AS | 25.74 | 4.55E-05 | 7.09E-03 | -1.26 | 2.19E-02 | -2.42 | 1.18E-04 |
| SLC25A42 | 25.69 | 4.62E-05 | 7.14E-03 | -1.71 | 3.36E-03 | -2.06 | 7.84E-04 |
| PGD | 25.66 | 4.75E-05 | 7.27E-03 | -1.68 | 3.87E-03 | -2.08 | 6.92E-04 |
| PLAC8L1 | 25.54 | 5.13E-05 | 7.75E-03 | -1.39 | 1.27E-02 | -2.30 | 2.24E-04 |
| DTNA | 25.54 | 5.14E-05 | 7.75E-03 | 1.61 | 5.09E-03 | 2.12 | 5.59E-04 |
| FPR1 | 25.41 | 5.34E-05 | 7.98E-03 | -1.42 | 1.16E-02 | -2.27 | 2.62E-04 |
| MAPKAPK3 | 25.31 | 5.59E-05 | 8.18E-03 | -1.77 | 2.62E-03 | -1.96 | 1.22E-03 |
| FGF22 | 25.29 | 5.61E-05 | 8.18E-03 | -1.03 | 5.49E-02 | -2.54 | 5.86E-05 |
| SYCE3 | 25.28 | 5.65E-05 | 8.18E-03 | -1.63 | 4.67E-03 | -2.08 | 6.93E-04 |
| B3GALT6 | 25.29 | 5.61E-05 | 8.18E-03 | -0.98 | 6.66E-02 | -2.56 | 4.84E-05 |
| PSAP | 25.27 | 5.74E-05 | 8.24E-03 | -1.57 | 6.07E-03 | -2.13 | 5.35E-04 |
| IQCJ-SCHIP1 | 25.15 | 6.15E-05 | 8.77E-03 | 2.11 | 4.99E-04 | 1.55 | 6.91E-03 |
| C11ORF94 | 25.13 | 6.22E-05 | 8.80E-03 | -1.31 | 1.80E-02 | -2.33 | 1.95E-04 |
| SYNGAP1 | 25.03 | 6.49E-05 | 9.12E-03 | -0.87 | 9.77E-02 | -2.61 | 3.76E-05 |
| SURF1 | 25.01 | 6.55E-05 | 9.12E-03 | -1.64 | 4.48E-03 | -2.05 | 8.27E-04 |
| FLJ20712 | 24.98 | 6.71E-05 | 9.21E-03 | -1.75 | 2.77E-03 | -1.94 | 1.36E-03 |
| KBTBD6 | 24.98 | 6.69E-05 | 9.21E-03 | 1.48 | 8.71E-03 | 2.17 | 4.32E-04 |
| SNX18 | 24.95 | 6.82E-05 | 9.29E-03 | -1.49 | 8.46E-03 | -2.16 | 4.52E-04 |
| GPRIN2 | 24.92 | 6.89E-05 | 9.32E-03 | -1.06 | 4.90E-02 | -2.49 | 7.92E-05 |
| PDZK1IP1 | 24.90 | 7.02E-05 | 9.42E-03 | -0.92 | 8.19E-02 | -2.57 | 4.79E-05 |
| SIAE | 24.87 | 7.09E-05 | 9.45E-03 | -1.48 | 8.95E-03 | -2.17 | 4.44E-04 |
| ENO3 | 24.77 | 7.48E-05 | 9.90E-03 | -1.60 | 5.37E-03 | -2.06 | 7.77E-04 |
| PVALB | 24.73 | 7.66E-05 | 1.00E-02 | -1.76 | 2.67E-03 | -1.90 | 1.60E-03 |
| AASS | 24.74 | 7.66E-05 | 1.00E-02 | 1.74 | 2.95E-03 | 1.92 | 1.44E-03 |
| DDIT4L | 24.66 | 7.95E-05 | 1.02E-02 | -2.05 | 6.88E-04 | -1.57 | 6.43E-03 |
| TMEM245 | 24.65 | 7.97E-05 | 1.02E-02 | 1.27 | 2.14E-02 | -2.32 | 2.07E-04 |
| RP4-539M6.14 | 24.64 | 8.01E-05 | 1.02E-02 | -1.56 | 6.26E-03 | -2.08 | 7.12E-04 |
| IPP | 24.65 | 7.99E-05 | 1.02E-02 | -1.13 | 3.69E-02 | -2.41 | 1.21E-04 |
| ETV4 | 24.61 | 8.11E-05 | 1.02E-02 | -1.36 | 1.48E-02 | -2.24 | 3.06E-04 |
| PPP1R1B | 24.59 | 8.22E-05 | 1.03E-02 | -1.14 | 3.56E-02 | -2.40 | 1.29E-04 |
| DEFA4 | 24.51 | 8.67E-05 | 1.08E-02 | -1.94 | 1.18E-03 | -1.68 | 4.05E-03 |
| IDI2 | 24.48 | 8.78E-05 | 1.08E-02 | 2.08 | 6.01E-04 | 1.51 | 8.05E-03 |
| ADAM22 | 24.42 | 9.07E-05 | 1.11E-02 | -1.90 | 1.43E-03 | -1.72 | 3.47E-03 |
| IRGC | 24.43 | 9.01E-05 | 1.11E-02 | -1.25 | 2.33E-02 | -2.31 | 2.12E-04 |
| ZNF296 | 24.40 | 9.17E-05 | 1.11E-02 | -0.95 | 7.29E-02 | -2.51 | 6.89E-05 |
| HSPA4 | 24.34 | 9.52E-05 | 1.13E-02 | -1.06 | 4.81E-02 | -2.44 | 1.08E-04 |
| ATP9B | 24.33 | 9.55E-05 | 1.13E-02 | -1.64 | 4.61E-03 | -1.98 | 1.13E-03 |
| PKP3 | 24.35 | 9.43E-05 | 1.13E-02 | -1.15 | 3.42E-02 | -2.37 | 1.50E-04 |
| VCL | 24.32 | 9.57E-05 | 1.13E-02 | -1.26 | 2.23E-02 | -2.29 | 2.35E-04 |
| KRT33A | 24.29 | 9.77E-05 | 1.15E-02 | -1.02 | 5.63E-02 | -2.46 | 9.46E-05 |
| IL17D | 24.21 | 1.01E-04 | 1.17E-02 | 1.72 | 3.25E-03 | 1.89 | 1.70E-03 |
| RAPH1 | 24.19 | 1.01E-04 | 1.18E-02 | -2.12 | 4.82E-04 | -1.43 | 1.16E-02 |
| PGC | 24.11 | 1.06E-04 | 1.22E-02 | -1.28 | 2.02E-02 | -2.25 | 2.87E-04 |
| TGFBRAP1 | 24.05 | 1.08E-04 | 1.23E-02 | -1.67 | 3.95E-03 | -1.91 | 1.52E-03 |
| SEPT7P2 | 24.06 | 1.08E-04 | 1.23E-02 | 1.39 | 1.30E-02 | 2.16 | 4.61E-04 |
| LOC285766 | 24.01 | 1.10E-04 | 1.25E-02 | -1.42 | 1.15E-02 | -2.13 | 5.34E-04 |
| OPRL1 | 23.96 | 1.13E-04 | 1.27E-02 | -1.39 | 1.31E-02 | -2.16 | 4.80E-04 |
| BIN1 | 23.90 | 1.17E-04 | 1.30E-02 | -1.83 | 2.01E-03 | -1.74 | 3.21E-03 |
| RP11-116D17.1 | 23.87 | 1.20E-04 | 1.33E-02 | -1.46 | 9.54E-03 | -2.08 | 6.86E-04 |
| UCKL1 | 23.80 | 1.23E-04 | 1.34E-02 | -1.45 | 9.96E-03 | -2.09 | 6.83E-04 |
| CIB2 | 23.79 | 1.24E-04 | 1.34E-02 | -1.75 | 2.86E-03 | -1.81 | 2.39E-03 |
| CA12 | 23.80 | 1.23E-04 | 1.34E-02 | -1.67 | 4.02E-03 | -1.89 | 1.69E-03 |
| WRN | 23.83 | 1.21E-04 | 1.34E-02 | -1.69 | 3.69E-03 | -1.87 | 1.82E-03 |
| LOC285419 | 23.81 | 1.23E-04 | 1.34E-02 | -1.94 | 1.20E-03 | -1.60 | 5.66E-03 |
| NDRG2 | 23.74 | 1.26E-04 | 1.34E-02 | -1.37 | 1.39E-02 | -2.15 | 5.03E-04 |
| TMSB4Y | 23.77 | 1.25E-04 | 1.34E-02 | -0.96 | 7.05E-02 | -2.45 | 9.77E-05 |
| PAPD5 | 23.74 | 1.26E-04 | 1.34E-02 | 1.15 | 3.43E-02 | 2.32 | 2.04E-04 |
| ACTL9 | 23.71 | 1.28E-04 | 1.35E-02 | -0.74 | 1.57E-01 | -2.58 | 4.53E-05 |
| ASMTL-AS1 | 23.66 | 1.32E-04 | 1.37E-02 | -1.39 | 1.29E-02 | -2.12 | 5.66E-04 |
| MIR7110 | 23.66 | 1.32E-04 | 1.37E-02 | -2.67 | 2.39E-05 | -0.51 | 3.04E-01 |
| LOC101060747 | 23.60 | 1.35E-04 | 1.38E-02 | -1.92 | 1.29E-03 | -1.59 | 5.80E-03 |
| HSPB6 | 23.60 | 1.35E-04 | 1.38E-02 | 1.93 | 1.26E-03 | 1.59 | 5.96E-03 |
| SLC13A4 | 23.60 | 1.35E-04 | 1.38E-02 | -1.12 | 3.84E-02 | -2.32 | 1.96E-04 |
| ANKRD46 | 23.50 | 1.41E-04 | 1.42E-02 | -0.92 | 8.11E-02 | -2.45 | 9.73E-05 |
| PPP1R15A | 23.50 | 1.41E-04 | 1.42E-02 | -1.50 | 8.25E-03 | -2.02 | 9.55E-04 |
| ADAM11 | 23.49 | 1.42E-04 | 1.42E-02 | -2.40 | 9.68E-05 | -0.92 | 8.21E-02 |
| LOC100507054 | 23.52 | 1.40E-04 | 1.42E-02 | -1.63 | 4.68E-03 | -1.89 | 1.67E-03 |
| MYH1 | 23.42 | 1.46E-04 | 1.46E-02 | -2.00 | 8.65E-04 | -1.47 | 9.49E-03 |
| KALP | 23.40 | 1.48E-04 | 1.47E-02 | -1.46 | 9.69E-03 | -2.04 | 8.54E-04 |
| RNF13 | 23.37 | 1.50E-04 | 1.47E-02 | -2.10 | 5.31E-04 | -1.35 | 1.58E-02 |
| RP4-657D16.3 | 23.36 | 1.51E-04 | 1.47E-02 | -1.75 | 2.84E-03 | -1.75 | 2.98E-03 |
| ITPR3 | 23.39 | 1.49E-04 | 1.47E-02 | 1.62 | 4.93E-03 | -1.89 | 1.69E-03 |
| ANKMY1 | 23.35 | 1.52E-04 | 1.47E-02 | -0.58 | 2.58E-01 | -2.64 | 3.29E-05 |
| C3ORF43 | 23.29 | 1.55E-04 | 1.48E-02 | 1.66 | 4.08E-03 | 1.83 | 2.14E-03 |
| SMIM24 | 23.27 | 1.56E-04 | 1.48E-02 | -1.11 | 4.09E-02 | -2.31 | 2.17E-04 |
| ANO1 | 23.26 | 1.56E-04 | 1.48E-02 | -1.16 | 3.31E-02 | -2.26 | 2.68E-04 |
| SLC52A2 | 23.30 | 1.55E-04 | 1.48E-02 | -2.00 | 8.85E-04 | -1.46 | 9.85E-03 |
| MAPK14 | 23.25 | 1.57E-04 | 1.48E-02 | -0.80 | 1.25E-01 | -2.51 | 7.18E-05 |
| PRDM16 | 23.32 | 1.54E-04 | 1.48E-02 | -1.68 | 3.80E-03 | -1.82 | 2.28E-03 |
| GP2 | 23.19 | 1.61E-04 | 1.50E-02 | -1.34 | 1.61E-02 | -2.12 | 5.73E-04 |
| LINC01127 | 23.20 | 1.60E-04 | 1.50E-02 | -1.13 | 3.64E-02 | -2.27 | 2.51E-04 |
| LPP | 23.10 | 1.70E-04 | 1.55E-02 | 1.32 | 1.70E-02 | 2.12 | 5.67E-04 |
| TSFM | 23.09 | 1.70E-04 | 1.55E-02 | 1.61 | 5.07E-03 | 1.86 | 1.91E-03 |
| PIK3R4 | 23.11 | 1.69E-04 | 1.55E-02 | 0.24 | 6.35E-01 | -2.77 | 1.51E-05 |
| DEFA1 | 23.11 | 1.69E-04 | 1.55E-02 | -1.19 | 2.91E-02 | -2.23 | 3.29E-04 |
| LOC101928707 | 23.11 | 1.69E-04 | 1.55E-02 | -0.86 | 1.03E-01 | -2.46 | 9.26E-05 |
| SRMP1 | 22.98 | 1.80E-04 | 1.62E-02 | -1.02 | 5.76E-02 | -2.34 | 1.77E-04 |
| RFX2 | 22.97 | 1.81E-04 | 1.63E-02 | 0.84 | 1.08E-01 | -2.46 | 9.50E-05 |
| EXOC3L1 | 22.79 | 1.97E-04 | 1.72E-02 | -1.51 | 7.84E-03 | -1.93 | 1.43E-03 |
| FN1 | 22.80 | 1.97E-04 | 1.72E-02 | -1.34 | 1.57E-02 | -2.08 | 7.14E-04 |
| SAMD11 | 22.83 | 1.94E-04 | 1.72E-02 | -1.01 | 5.86E-02 | -2.33 | 1.89E-04 |
| C20ORF195 | 22.79 | 1.97E-04 | 1.72E-02 | -0.68 | 1.86E-01 | -2.53 | 6.04E-05 |
| MSH3 | 22.83 | 1.94E-04 | 1.72E-02 | 1.61 | 5.15E-03 | 1.83 | 2.14E-03 |
| RUNDC3B | 22.79 | 1.97E-04 | 1.72E-02 | -1.79 | 2.42E-03 | -1.65 | 4.65E-03 |
| RNF126 | 22.75 | 2.00E-04 | 1.74E-02 | -1.25 | 2.28E-02 | -2.15 | 5.03E-04 |
| FBXO17 | 22.74 | 2.02E-04 | 1.75E-02 | -0.95 | 7.30E-02 | -2.36 | 1.58E-04 |
| SNRPN | 22.71 | 2.03E-04 | 1.75E-02 | -1.82 | 2.11E-03 | -1.60 | 5.54E-03 |
| SHROOM2 | 22.70 | 2.05E-04 | 1.76E-02 | -1.49 | 8.47E-03 | -1.93 | 1.39E-03 |
| SSU72 | 22.69 | 2.06E-04 | 1.76E-02 | -1.05 | 4.98E-02 | -2.29 | 2.37E-04 |
| LOC339685 | 22.64 | 2.10E-04 | 1.78E-02 | -1.05 | 5.10E-02 | -2.29 | 2.37E-04 |
| RP5-944M2.2 | 22.63 | 2.11E-04 | 1.79E-02 | -1.56 | 6.29E-03 | -1.86 | 1.93E-03 |
| LOC100505841 | 22.61 | 2.13E-04 | 1.79E-02 | -1.56 | 6.43E-03 | -1.86 | 1.91E-03 |
| LINC01010 | 22.60 | 2.14E-04 | 1.79E-02 | -1.63 | 4.63E-03 | -1.78 | 2.67E-03 |
| LOC100507316 | 22.59 | 2.15E-04 | 1.79E-02 | 1.23 | 2.43E-02 | 2.14 | 5.10E-04 |
| PSG4 | 22.55 | 2.18E-04 | 1.81E-02 | -1.34 | 1.60E-02 | -2.05 | 7.94E-04 |
| NFYC | 22.53 | 2.21E-04 | 1.83E-02 | -1.86 | 1.77E-03 | -1.54 | 7.22E-03 |
| CPED1 | 22.50 | 2.25E-04 | 1.85E-02 | 1.62 | 5.04E-03 | 1.79 | 2.58E-03 |
| AK097836 | 22.49 | 2.25E-04 | 1.85E-02 | -1.64 | 4.43E-03 | -1.76 | 2.94E-03 |
| NUCB1 | 22.48 | 2.27E-04 | 1.86E-02 | -1.34 | 1.58E-02 | -2.05 | 8.29E-04 |
| TMEM18 | 22.46 | 2.29E-04 | 1.86E-02 | -0.94 | 7.75E-02 | -2.35 | 1.71E-04 |
| TRIM42 | 22.45 | 2.30E-04 | 1.86E-02 | -1.70 | 3.44E-03 | -1.69 | 3.88E-03 |
| GATSL3 | 22.42 | 2.34E-04 | 1.88E-02 | -0.87 | 9.97E-02 | -2.39 | 1.35E-04 |
| PRKCQ-AS1 | 22.38 | 2.38E-04 | 1.88E-02 | 1.81 | 2.22E-03 | 1.58 | 6.22E-03 |
| AMFR | 22.40 | 2.37E-04 | 1.88E-02 | -1.54 | 6.80E-03 | -1.85 | 2.02E-03 |
| FBXO39 | 22.39 | 2.37E-04 | 1.88E-02 | -1.55 | 6.73E-03 | -1.84 | 2.04E-03 |
| MRGPRX2 | 22.39 | 2.37E-04 | 1.88E-02 | -1.61 | 5.11E-03 | -1.78 | 2.69E-03 |
| GAPDHP73 | 22.36 | 2.41E-04 | 1.90E-02 | -1.82 | 2.11E-03 | -1.56 | 6.62E-03 |
| AKTIP | 22.33 | 2.44E-04 | 1.92E-02 | -2.04 | 7.48E-04 | -1.30 | 1.89E-02 |
| NRG1-IT1 | 22.32 | 2.46E-04 | 1.93E-02 | -1.21 | 2.69E-02 | -2.13 | 5.31E-04 |
| EDC4 | 22.31 | 2.48E-04 | 1.93E-02 | -1.49 | 8.63E-03 | -1.89 | 1.66E-03 |
| TFPI | 22.28 | 2.51E-04 | 1.94E-02 | -0.20 | 6.84E-01 | -2.70 | 2.12E-05 |
| SRC | 22.28 | 2.51E-04 | 1.94E-02 | -0.81 | 1.23E-01 | -2.42 | 1.18E-04 |
| LOC148709 | 22.24 | 2.55E-04 | 1.96E-02 | -1.00 | 6.15E-02 | -2.28 | 2.41E-04 |
| SMAD9 | 22.22 | 2.57E-04 | 1.97E-02 | 1.78 | 2.47E-03 | 1.58 | 6.04E-03 |
| PPIE | 22.21 | 2.58E-04 | 1.97E-02 | -1.10 | 4.23E-02 | -2.21 | 3.55E-04 |
| MFAP4 | 22.20 | 2.60E-04 | 1.97E-02 | -1.34 | 1.60E-02 | -2.02 | 9.43E-04 |
| CCDC101 | 22.20 | 2.61E-04 | 1.97E-02 | -1.75 | 2.85E-03 | -1.61 | 5.30E-03 |
| MNX1-AS1 | 22.19 | 2.62E-04 | 1.97E-02 | -1.16 | 3.35E-02 | -2.16 | 4.53E-04 |
| GOSR2 | 22.19 | 2.64E-04 | 1.98E-02 | -0.81 | 1.21E-01 | -2.41 | 1.26E-04 |
| DAGLB | 22.14 | 2.69E-04 | 2.01E-02 | -1.37 | 1.40E-02 | -1.98 | 1.11E-03 |
| FKBPL | 22.12 | 2.73E-04 | 2.03E-02 | -0.87 | 9.77E-02 | -2.36 | 1.61E-04 |
| CDKAL1 | 22.12 | 2.73E-04 | 2.03E-02 | -2.22 | 2.77E-04 | -1.02 | 5.68E-02 |
| LINGO3 | 22.04 | 2.85E-04 | 2.10E-02 | -1.30 | 1.85E-02 | -2.03 | 8.83E-04 |
| PAM | 22.03 | 2.87E-04 | 2.10E-02 | 1.79 | 2.33E-03 | 1.55 | 7.07E-03 |
| INPP5A | 22.02 | 2.87E-04 | 2.10E-02 | -1.41 | 1.20E-02 | -1.93 | 1.38E-03 |
| TPD52L1 | 22.00 | 2.89E-04 | 2.11E-02 | 1.81 | 2.16E-03 | 1.52 | 7.73E-03 |
| SLC16A8 | 22.00 | 2.90E-04 | 2.11E-02 | -0.95 | 7.41E-02 | -2.30 | 2.26E-04 |
| CD53 | 21.98 | 2.93E-04 | 2.12E-02 | -2.15 | 4.13E-04 | -1.11 | 4.10E-02 |
| LMO2 | 21.88 | 3.04E-04 | 2.15E-02 | -1.88 | 1.56E-03 | -1.43 | 1.14E-02 |
| AZIN2 | 21.93 | 3.00E-04 | 2.15E-02 | -0.37 | 4.61E-01 | -2.61 | 3.76E-05 |
| LAMB3 | 21.92 | 3.00E-04 | 2.15E-02 | -1.24 | 2.39E-02 | -2.07 | 7.28E-04 |
| ATP1A1-AS1 | 21.94 | 2.98E-04 | 2.15E-02 | 1.18 | 3.00E-02 | -2.12 | 5.73E-04 |
| GMIP | 21.88 | 3.05E-04 | 2.15E-02 | 0.81 | 1.22E-01 | -2.38 | 1.45E-04 |
| LOC101927943 | 21.89 | 3.04E-04 | 2.15E-02 | 1.51 | 7.71E-03 | 1.82 | 2.29E-03 |
| MROH2B | 21.89 | 3.04E-04 | 2.15E-02 | 1.11 | 3.95E-02 | -2.17 | 4.47E-04 |
| LGR5 | 21.85 | 3.08E-04 | 2.15E-02 | 1.55 | 6.57E-03 | 1.77 | 2.74E-03 |
| LRRK2 | 21.84 | 3.09E-04 | 2.15E-02 | -1.57 | 6.06E-03 | -1.75 | 2.98E-03 |
| CPEB2 | 21.86 | 3.08E-04 | 2.15E-02 | -0.92 | 8.16E-02 | -2.30 | 2.20E-04 |
| PSIP1 | 21.78 | 3.16E-04 | 2.15E-02 | 1.34 | 1.55E-02 | 1.96 | 1.20E-03 |
| RABL6 | 21.80 | 3.15E-04 | 2.15E-02 | -1.16 | 3.25E-02 | -2.12 | 5.69E-04 |
| ARL5C | 21.79 | 3.15E-04 | 2.15E-02 | -1.46 | 9.47E-03 | -1.85 | 1.96E-03 |
| GPR98 | 21.79 | 3.15E-04 | 2.15E-02 | -1.30 | 1.85E-02 | -2.01 | 1.00E-03 |
| RP11-587D21.4 | 21.78 | 3.16E-04 | 2.15E-02 | -1.36 | 1.46E-02 | -1.95 | 1.27E-03 |
| LOC100507506 | 21.81 | 3.12E-04 | 2.15E-02 | -0.86 | 1.01E-01 | -2.34 | 1.82E-04 |
| PRDM12 | 21.77 | 3.18E-04 | 2.16E-02 | -0.89 | 9.13E-02 | -2.32 | 2.05E-04 |
| BCL2L2 | 21.75 | 3.21E-04 | 2.17E-02 | -1.54 | 7.03E-03 | -1.78 | 2.69E-03 |
| ZNF710 | 21.70 | 3.27E-04 | 2.20E-02 | -1.31 | 1.77E-02 | -1.98 | 1.09E-03 |
| LOC101927151 | 21.69 | 3.30E-04 | 2.21E-02 | -1.64 | 4.56E-03 | -1.67 | 4.28E-03 |
| TWIST2 | 21.69 | 3.29E-04 | 2.21E-02 | -0.94 | 7.79E-02 | -2.28 | 2.50E-04 |
| HBG1 | 21.67 | 3.32E-04 | 2.21E-02 | -2.07 | 6.36E-04 | -1.18 | 3.09E-02 |
| BATF | 21.64 | 3.37E-04 | 2.24E-02 | -1.13 | 3.69E-02 | -2.13 | 5.41E-04 |
| RABEP2 | 21.62 | 3.40E-04 | 2.25E-02 | -1.88 | 1.58E-03 | -1.40 | 1.27E-02 |
| ACVR1 | 21.58 | 3.45E-04 | 2.28E-02 | -1.62 | 4.88E-03 | -1.67 | 4.22E-03 |
| HBM | 21.57 | 3.49E-04 | 2.29E-02 | -1.21 | 2.72E-02 | -2.06 | 7.62E-04 |
| BCKDHB | 21.55 | 3.51E-04 | 2.30E-02 | 1.48 | 8.75E-03 | 1.81 | 2.39E-03 |
| TBC1D20 | 21.53 | 3.54E-04 | 2.31E-02 | -1.08 | 4.52E-02 | -2.16 | 4.66E-04 |
| METTL13 | 21.52 | 3.55E-04 | 2.31E-02 | -1.18 | 3.07E-02 | -2.08 | 6.90E-04 |
| LOC100289061 | 21.50 | 3.61E-04 | 2.34E-02 | -1.09 | 4.27E-02 | -2.15 | 5.03E-04 |
| TCIRG1 | 21.38 | 3.78E-04 | 2.34E-02 | -1.84 | 1.89E-03 | -1.42 | 1.20E-02 |
| RABGGTA | 21.37 | 3.80E-04 | 2.34E-02 | -1.77 | 2.53E-03 | -1.49 | 9.04E-03 |
| CHUK | 21.46 | 3.66E-04 | 2.34E-02 | -1.67 | 4.07E-03 | -1.61 | 5.37E-03 |
| DDX54 | 21.38 | 3.79E-04 | 2.34E-02 | 0.56 | 2.74E-01 | -2.48 | 8.31E-05 |
| EPHA1 | 21.37 | 3.80E-04 | 2.34E-02 | -0.84 | 1.09E-01 | -2.31 | 2.11E-04 |
| MTMR3 | 21.42 | 3.72E-04 | 2.34E-02 | 1.18 | 3.00E-02 | -2.07 | 7.42E-04 |
| WFIKKN1 | 21.47 | 3.65E-04 | 2.34E-02 | -1.15 | 3.37E-02 | -2.10 | 6.45E-04 |
| FGFR4 | 21.42 | 3.74E-04 | 2.34E-02 | -0.94 | 7.71E-02 | -2.25 | 2.90E-04 |
| TMEFF2 | 21.45 | 3.68E-04 | 2.34E-02 | 1.22 | 2.62E-02 | 2.04 | 8.38E-04 |
| SPATC1L | 21.41 | 3.74E-04 | 2.34E-02 | -0.66 | 2.01E-01 | -2.43 | 1.12E-04 |
| LINC01120 | 21.44 | 3.70E-04 | 2.34E-02 | -1.05 | 5.03E-02 | -2.17 | 4.39E-04 |
| JRK | 21.42 | 3.73E-04 | 2.34E-02 | -1.27 | 2.12E-02 | -1.99 | 1.05E-03 |
| RP11-669M16.1 | 21.41 | 3.75E-04 | 2.34E-02 | -0.90 | 8.79E-02 | -2.27 | 2.55E-04 |
| GALNT2 | 21.43 | 3.72E-04 | 2.34E-02 | -1.17 | 3.21E-02 | -2.08 | 6.93E-04 |
| GPC3 | 21.35 | 3.81E-04 | 2.35E-02 | -1.46 | 9.73E-03 | -1.81 | 2.37E-03 |
| MORC4 | 21.32 | 3.87E-04 | 2.36E-02 | -1.23 | 2.48E-02 | -2.02 | 9.50E-04 |
| FCGBP | 21.34 | 3.85E-04 | 2.36E-02 | 1.39 | 1.27E-02 | 1.87 | 1.83E-03 |
| WDR62 | 21.32 | 3.87E-04 | 2.36E-02 | -1.17 | 3.13E-02 | -2.07 | 7.51E-04 |
| SLC24A3 | 21.25 | 3.97E-04 | 2.40E-02 | -1.50 | 8.10E-03 | -1.75 | 2.99E-03 |
| SYNC | 21.27 | 3.96E-04 | 2.40E-02 | -1.97 | 1.01E-03 | -1.24 | 2.40E-02 |
| C10ORF113 | 21.25 | 3.97E-04 | 2.40E-02 | -1.13 | 3.72E-02 | -2.09 | 6.53E-04 |
| BCRP3 | 21.22 | 4.05E-04 | 2.40E-02 | -1.48 | 8.84E-03 | -1.77 | 2.79E-03 |
| OXT | 21.21 | 4.05E-04 | 2.40E-02 | -0.81 | 1.21E-01 | -2.32 | 2.04E-04 |
| VPS37C | 21.23 | 4.02E-04 | 2.40E-02 | -1.04 | 5.16E-02 | -2.16 | 4.76E-04 |
| LOC100287728 | 21.23 | 4.02E-04 | 2.40E-02 | -1.14 | 3.51E-02 | -2.08 | 6.99E-04 |
| ARMC12 | 21.22 | 4.05E-04 | 2.40E-02 | -1.19 | 2.91E-02 | -2.04 | 8.50E-04 |
| C19ORF25 | 21.20 | 4.08E-04 | 2.41E-02 | -1.27 | 2.09E-02 | -1.97 | 1.19E-03 |
| RP3-327A19.5 | 21.19 | 4.09E-04 | 2.42E-02 | 1.47 | 9.31E-03 | 1.78 | 2.68E-03 |
| NDUFA3 | 21.17 | 4.14E-04 | 2.43E-02 | 1.48 | 9.01E-03 | 1.77 | 2.81E-03 |
| IFT52 | 21.17 | 4.14E-04 | 2.43E-02 | -0.63 | 2.24E-01 | -2.43 | 1.13E-04 |
| PPM1B | 21.16 | 4.17E-04 | 2.44E-02 | 1.85 | 1.79E-03 | 1.38 | 1.42E-02 |
| RP5-1031D4.2 | 21.10 | 4.26E-04 | 2.45E-02 | -1.22 | 2.57E-02 | -2.00 | 1.02E-03 |
| HEATR2 | 21.12 | 4.23E-04 | 2.45E-02 | -1.00 | 6.19E-02 | -2.18 | 4.18E-04 |
| ARSE | 21.12 | 4.23E-04 | 2.45E-02 | -0.95 | 7.26E-02 | -2.21 | 3.57E-04 |
| ARHGDIG | 21.12 | 4.23E-04 | 2.45E-02 | -0.98 | 6.60E-02 | -2.19 | 3.92E-04 |
| C7ORF73 | 21.10 | 4.26E-04 | 2.45E-02 | 0.98 | 6.63E-02 | -2.19 | 3.94E-04 |
| MYH8 | 21.07 | 4.31E-04 | 2.47E-02 | -1.17 | 3.12E-02 | -2.04 | 8.51E-04 |
| KRTAP10-12 | 21.06 | 4.33E-04 | 2.48E-02 | -1.40 | 1.26E-02 | -1.83 | 2.13E-03 |
| ADCK3 | 21.05 | 4.35E-04 | 2.48E-02 | -1.24 | 2.35E-02 | -1.98 | 1.14E-03 |
| ENSA | 21.04 | 4.37E-04 | 2.48E-02 | -1.31 | 1.77E-02 | -1.91 | 1.52E-03 |
| LINC01272 | 20.87 | 4.68E-04 | 2.49E-02 | -1.29 | 1.95E-02 | -1.91 | 1.51E-03 |
| RRP12 | 20.88 | 4.67E-04 | 2.49E-02 | -1.29 | 1.96E-02 | -1.92 | 1.50E-03 |
| SELL | 20.90 | 4.65E-04 | 2.49E-02 | -1.54 | 6.90E-03 | -1.67 | 4.19E-03 |
| ANP32E | 20.93 | 4.59E-04 | 2.49E-02 | 1.07 | 4.73E-02 | 2.11 | 6.04E-04 |
| LOC101928623 | 20.97 | 4.51E-04 | 2.49E-02 | -0.87 | 9.71E-02 | -2.25 | 2.88E-04 |
| DMBT1 | 20.96 | 4.53E-04 | 2.49E-02 | -1.41 | 1.21E-02 | -1.81 | 2.33E-03 |
| TMTC1 | 20.89 | 4.66E-04 | 2.49E-02 | 1.63 | 4.64E-03 | 1.57 | 6.27E-03 |
| FAM170A | 20.92 | 4.62E-04 | 2.49E-02 | -1.39 | 1.27E-02 | -1.82 | 2.26E-03 |
| PEX16 | 20.93 | 4.60E-04 | 2.49E-02 | -1.00 | 6.10E-02 | -2.16 | 4.68E-04 |
| FAM78B | 20.93 | 4.60E-04 | 2.49E-02 | -1.09 | 4.28E-02 | -2.09 | 6.67E-04 |
| CHAF1B | 20.96 | 4.53E-04 | 2.49E-02 | -1.32 | 1.74E-02 | -1.90 | 1.61E-03 |
| LOC283482 | 20.90 | 4.65E-04 | 2.49E-02 | -1.38 | 1.35E-02 | -1.83 | 2.15E-03 |
| HCRT | 20.90 | 4.64E-04 | 2.49E-02 | -1.56 | 6.47E-03 | -1.66 | 4.46E-03 |
| HHIP-AS1 | 20.99 | 4.47E-04 | 2.49E-02 | -0.13 | 7.86E-01 | -2.63 | 3.53E-05 |
| TSC2 | 20.94 | 4.58E-04 | 2.49E-02 | -1.14 | 3.56E-02 | -2.05 | 7.99E-04 |
| ZNF362 | 20.93 | 4.59E-04 | 2.49E-02 | -0.82 | 1.17E-01 | -2.28 | 2.43E-04 |
| SNHG12 | 20.97 | 4.50E-04 | 2.49E-02 | -0.78 | 1.36E-01 | -2.32 | 2.05E-04 |
| CDSN | 20.98 | 4.49E-04 | 2.49E-02 | -1.10 | 4.13E-02 | -2.09 | 6.74E-04 |
| PIF1 | 20.96 | 4.53E-04 | 2.49E-02 | -1.69 | 3.69E-03 | -1.53 | 7.60E-03 |
| RET | 20.92 | 4.62E-04 | 2.49E-02 | -0.71 | 1.73E-01 | -2.36 | 1.66E-04 |
| MLXIP | 21.01 | 4.43E-04 | 2.49E-02 | -1.22 | 2.63E-02 | -2.00 | 1.04E-03 |
| LOC100506235 | 21.02 | 4.39E-04 | 2.49E-02 | 1.41 | 1.19E-02 | -1.82 | 2.29E-03 |
| LOC340107 | 20.86 | 4.70E-04 | 2.49E-02 | -0.59 | 2.50E-01 | -2.42 | 1.18E-04 |
| GUCA2B | 20.85 | 4.72E-04 | 2.50E-02 | -1.06 | 4.81E-02 | -2.10 | 6.17E-04 |
| ADPRHL1 | 20.84 | 4.74E-04 | 2.50E-02 | -1.84 | 1.91E-03 | -1.35 | 1.56E-02 |
| IGKV1OR2-108 | 20.82 | 4.79E-04 | 2.52E-02 | -1.20 | 2.80E-02 | -1.99 | 1.08E-03 |
| XIRP1 | 20.80 | 4.81E-04 | 2.52E-02 | 1.47 | 9.13E-03 | 1.73 | 3.33E-03 |
| LOC101930630 | 20.79 | 4.84E-04 | 2.53E-02 | -1.29 | 1.93E-02 | -1.90 | 1.59E-03 |
| RMST | 20.79 | 4.83E-04 | 2.53E-02 | 1.33 | 1.68E-02 | -1.87 | 1.82E-03 |
| ERC1 | 20.78 | 4.86E-04 | 2.53E-02 | 1.31 | 1.78E-02 | 1.88 | 1.73E-03 |
| GNPTAB | 20.75 | 4.90E-04 | 2.54E-02 | -0.79 | 1.30E-01 | -2.29 | 2.40E-04 |
| ZNF214 | 20.74 | 4.93E-04 | 2.55E-02 | 1.18 | 3.06E-02 | 2.00 | 1.03E-03 |
| SCARNA2 | 20.74 | 4.93E-04 | 2.55E-02 | -1.03 | 5.38E-02 | -2.11 | 5.84E-04 |
| OSCAR | 20.71 | 4.99E-04 | 2.57E-02 | -1.38 | 1.33E-02 | -1.81 | 2.39E-03 |
| RP11-677O4.2 | 20.69 | 5.03E-04 | 2.57E-02 | -1.24 | 2.40E-02 | -1.94 | 1.33E-03 |
| PASK | 20.70 | 5.03E-04 | 2.57E-02 | 1.15 | 3.38E-02 | 2.02 | 9.49E-04 |
| GSTO1 | 20.68 | 5.07E-04 | 2.58E-02 | 2.03 | 7.58E-04 | 1.10 | 4.27E-02 |
| LOC101930301 | 20.67 | 5.09E-04 | 2.58E-02 | -0.75 | 1.50E-01 | -2.31 | 2.16E-04 |
| GPD2 | 20.65 | 5.13E-04 | 2.58E-02 | -1.63 | 4.70E-03 | -1.55 | 6.99E-03 |
| TMTC4 | 20.65 | 5.13E-04 | 2.58E-02 | -1.25 | 2.31E-02 | -1.93 | 1.42E-03 |
| LOC101928973 | 20.67 | 5.09E-04 | 2.58E-02 | -0.78 | 1.34E-01 | -2.28 | 2.43E-04 |
| TBX1 | 20.65 | 5.13E-04 | 2.58E-02 | -0.66 | 2.00E-01 | -2.36 | 1.64E-04 |
| TGFB2 | 20.67 | 5.08E-04 | 2.58E-02 | 1.22 | 2.55E-02 | -1.95 | 1.27E-03 |
| PPP3R1 | 20.63 | 5.18E-04 | 2.58E-02 | -1.85 | 1.81E-03 | -1.31 | 1.83E-02 |
| SLC12A9 | 20.62 | 5.18E-04 | 2.58E-02 | -0.99 | 6.42E-02 | -2.14 | 5.18E-04 |
| FCGR2C | 20.63 | 5.18E-04 | 2.58E-02 | -0.97 | 6.92E-02 | -2.16 | 4.79E-04 |
| NPLOC4 | 20.61 | 5.22E-04 | 2.59E-02 | -1.55 | 6.74E-03 | -1.63 | 4.96E-03 |
| FOXP2 | 20.60 | 5.24E-04 | 2.60E-02 | 1.04 | 5.28E-02 | -2.10 | 6.35E-04 |
| LMNTD2 | 20.56 | 5.34E-04 | 2.64E-02 | -0.54 | 2.95E-01 | -2.42 | 1.16E-04 |
| CTSA | 20.50 | 5.49E-04 | 2.64E-02 | 1.41 | 1.19E-02 | 1.76 | 2.97E-03 |
| IL27RA | 20.51 | 5.48E-04 | 2.64E-02 | -0.97 | 6.89E-02 | -2.14 | 5.10E-04 |
| P2RX6 | 20.52 | 5.46E-04 | 2.64E-02 | -0.99 | 6.36E-02 | -2.13 | 5.50E-04 |
| MAFF | 20.52 | 5.47E-04 | 2.64E-02 | -1.57 | 5.97E-03 | 1.59 | 5.88E-03 |
| NMT2 | 20.52 | 5.45E-04 | 2.64E-02 | 1.32 | 1.75E-02 | 1.85 | 2.00E-03 |
| BSCL2 | 20.53 | 5.44E-04 | 2.64E-02 | -1.01 | 5.81E-02 | -2.11 | 6.01E-04 |
| SCD5 | 20.55 | 5.39E-04 | 2.64E-02 | -0.92 | 8.14E-02 | -2.18 | 4.25E-04 |
| BTF3P12 | 20.54 | 5.41E-04 | 2.64E-02 | -0.86 | 1.02E-01 | -2.22 | 3.41E-04 |
| SPATA25 | 20.52 | 5.47E-04 | 2.64E-02 | -0.62 | 2.31E-01 | -2.37 | 1.52E-04 |
| KCP | 20.53 | 5.44E-04 | 2.64E-02 | -0.76 | 1.43E-01 | -2.28 | 2.43E-04 |
| GUK1 | 20.46 | 5.61E-04 | 2.70E-02 | -1.53 | 7.19E-03 | -1.63 | 5.02E-03 |
| SRXN1 | 20.44 | 5.64E-04 | 2.70E-02 | -1.49 | 8.61E-03 | -1.67 | 4.22E-03 |
| PBRM1 | 20.45 | 5.64E-04 | 2.70E-02 | -1.59 | 5.67E-03 | -1.57 | 6.41E-03 |
| LOC101929592 | 20.42 | 5.68E-04 | 2.71E-02 | 2.02 | 7.99E-04 | 1.08 | 4.59E-02 |
| CLIP3 | 20.42 | 5.70E-04 | 2.71E-02 | 0.95 | 7.34E-02 | -2.15 | 5.03E-04 |
| ERBB2IP | 20.39 | 5.76E-04 | 2.72E-02 | -0.87 | 9.89E-02 | -2.20 | 3.77E-04 |
| ADAM6 | 20.39 | 5.77E-04 | 2.72E-02 | -0.95 | 7.35E-02 | -2.14 | 5.09E-04 |
| LINC00671 | 20.39 | 5.77E-04 | 2.72E-02 | -1.08 | 4.48E-02 | -2.04 | 8.35E-04 |
| ZNF248 | 20.38 | 5.82E-04 | 2.72E-02 | -1.46 | 9.68E-03 | -1.69 | 3.88E-03 |
| GRB14 | 20.37 | 5.83E-04 | 2.72E-02 | -1.81 | 2.15E-03 | -1.32 | 1.76E-02 |
| LINC00982 | 20.37 | 5.82E-04 | 2.72E-02 | 0.80 | 1.26E-01 | -2.24 | 3.00E-04 |
| KIAA1210 | 20.38 | 5.80E-04 | 2.72E-02 | 1.43 | 1.10E-02 | 1.72 | 3.40E-03 |
| PTPLA | 20.35 | 5.88E-04 | 2.74E-02 | -0.59 | 2.53E-01 | -2.37 | 1.50E-04 |
| PTPN18 | 20.33 | 5.93E-04 | 2.75E-02 | 1.60 | 5.28E-03 | -1.54 | 7.27E-03 |
| ZCWPW1 | 20.32 | 5.96E-04 | 2.75E-02 | -1.23 | 2.46E-02 | -1.90 | 1.57E-03 |
| LOC101930296 | 20.31 | 5.99E-04 | 2.75E-02 | -1.52 | 7.47E-03 | -1.62 | 5.21E-03 |
| RP11-95D17.1 | 20.32 | 5.96E-04 | 2.75E-02 | -1.14 | 3.55E-02 | -1.99 | 1.09E-03 |
| TACC3 | 20.31 | 5.98E-04 | 2.75E-02 | -1.16 | 3.25E-02 | -1.97 | 1.20E-03 |
| DNAJC12 | 20.29 | 6.04E-04 | 2.77E-02 | 0.99 | 6.28E-02 | -2.10 | 6.25E-04 |
| KRT78 | 20.29 | 6.05E-04 | 2.77E-02 | -1.17 | 3.13E-02 | -1.95 | 1.26E-03 |
| EIF4G1 | 20.24 | 6.16E-04 | 2.80E-02 | -1.27 | 2.12E-02 | -1.86 | 1.90E-03 |
| KMT2D | 20.25 | 6.15E-04 | 2.80E-02 | -1.34 | 1.57E-02 | -1.79 | 2.55E-03 |
| MAP4 | 20.24 | 6.17E-04 | 2.80E-02 | -1.26 | 2.16E-02 | -1.86 | 1.86E-03 |
| ANKRD23 | 20.20 | 6.27E-04 | 2.81E-02 | -1.67 | 4.06E-03 | -1.46 | 1.01E-02 |
| OXCT1 | 20.19 | 6.30E-04 | 2.81E-02 | 1.33 | 1.62E-02 | 1.79 | 2.54E-03 |
| SSPO | 20.19 | 6.29E-04 | 2.81E-02 | -1.38 | 1.36E-02 | -1.75 | 3.02E-03 |
| NTN4 | 20.20 | 6.28E-04 | 2.81E-02 | 1.29 | 1.95E-02 | 1.84 | 2.11E-03 |
| ZNF282 | 20.21 | 6.25E-04 | 2.81E-02 | -1.36 | 1.43E-02 | -1.76 | 2.85E-03 |
| SOX1 | 20.22 | 6.23E-04 | 2.81E-02 | -1.49 | 8.41E-03 | -1.64 | 4.85E-03 |
| MXD1 | 20.19 | 6.30E-04 | 2.81E-02 | -0.72 | 1.67E-01 | -2.28 | 2.47E-04 |
| SLC16A7 | 20.17 | 6.35E-04 | 2.83E-02 | 1.61 | 5.10E-03 | 1.51 | 8.17E-03 |
| LOC101927766 | 20.15 | 6.41E-04 | 2.84E-02 | -1.14 | 3.56E-02 | -1.97 | 1.19E-03 |
| RPL35A | 20.15 | 6.40E-04 | 2.84E-02 | 1.44 | 1.04E-02 | 1.68 | 4.06E-03 |
| LOC101927460 | 20.14 | 6.45E-04 | 2.85E-02 | 1.19 | 2.91E-02 | 1.92 | 1.45E-03 |
| RP11-354I10.1 | 20.13 | 6.47E-04 | 2.86E-02 | -0.58 | 2.57E-01 | -2.36 | 1.66E-04 |
| GAS1 | 20.11 | 6.51E-04 | 2.86E-02 | 0.39 | 4.36E-01 | -2.45 | 9.84E-05 |
| SKAP2 | 20.11 | 6.53E-04 | 2.87E-02 | 1.80 | 2.25E-03 | 1.30 | 1.91E-02 |
| RP11-352G9.1 | 20.10 | 6.55E-04 | 2.87E-02 | -0.64 | 2.16E-01 | -2.32 | 2.00E-04 |
| SIX1 | 20.08 | 6.61E-04 | 2.88E-02 | 1.39 | 1.30E-02 | -1.73 | 3.35E-03 |
| ALDH18A1 | 20.08 | 6.61E-04 | 2.88E-02 | 1.12 | 3.82E-02 | -1.98 | 1.14E-03 |
| KIF26B | 20.07 | 6.64E-04 | 2.89E-02 | 1.61 | 5.05E-03 | -1.49 | 8.69E-03 |
| PACS2 | 20.06 | 6.65E-04 | 2.89E-02 | -1.44 | 1.06E-02 | -1.68 | 4.13E-03 |
| PEF1 | 20.06 | 6.67E-04 | 2.89E-02 | -1.63 | 4.78E-03 | -1.48 | 9.23E-03 |
| ICAM1 | 20.04 | 6.72E-04 | 2.90E-02 | 1.53 | 7.20E-03 | 1.58 | 6.18E-03 |
| LINC01395 | 20.04 | 6.72E-04 | 2.90E-02 | -1.03 | 5.36E-02 | -2.04 | 8.31E-04 |
| OSM | 20.03 | 6.77E-04 | 2.91E-02 | 1.31 | 1.83E-02 | -1.80 | 2.45E-03 |
| AURKC | 20.02 | 6.79E-04 | 2.91E-02 | -1.18 | 3.01E-02 | -1.92 | 1.49E-03 |
| PDZD3 | 19.99 | 6.89E-04 | 2.92E-02 | -1.24 | 2.43E-02 | -1.86 | 1.88E-03 |
| MAFK | 19.98 | 6.92E-04 | 2.92E-02 | -1.00 | 6.17E-02 | -2.07 | 7.42E-04 |
| KLHL1 | 19.98 | 6.91E-04 | 2.92E-02 | -0.70 | 1.78E-01 | -2.27 | 2.57E-04 |
| PCDHB6 | 19.99 | 6.90E-04 | 2.92E-02 | 0.82 | 1.19E-01 | -2.20 | 3.85E-04 |
| SLC35D2 | 19.98 | 6.93E-04 | 2.92E-02 | -0.67 | 1.93E-01 | -2.29 | 2.38E-04 |
| POM121L8P | 20.00 | 6.85E-04 | 2.92E-02 | -0.87 | 1.00E-01 | -2.16 | 4.51E-04 |
| ZNF490 | 20.00 | 6.86E-04 | 2.92E-02 | -1.19 | 2.89E-02 | -1.90 | 1.57E-03 |
| ART1 | 19.99 | 6.91E-04 | 2.92E-02 | -0.73 | 1.58E-01 | -2.25 | 2.90E-04 |
| PLEC | 19.94 | 7.04E-04 | 2.94E-02 | -1.27 | 2.15E-02 | -1.83 | 2.18E-03 |
| PGLYRP1 | 19.95 | 7.02E-04 | 2.94E-02 | -0.88 | 9.39E-02 | -2.15 | 4.96E-04 |
| MYH13 | 19.94 | 7.04E-04 | 2.94E-02 | -0.20 | 6.89E-01 | -2.52 | 6.78E-05 |
| PIEZO1 | 19.96 | 7.00E-04 | 2.94E-02 | -0.83 | 1.13E-01 | -2.18 | 4.12E-04 |
| CCDC85C | 19.92 | 7.12E-04 | 2.97E-02 | -0.73 | 1.59E-01 | -2.25 | 2.97E-04 |
| SLC9A9 | 19.91 | 7.17E-04 | 2.98E-02 | 1.55 | 6.65E-03 | 1.54 | 7.16E-03 |
| SERP2 | 19.89 | 7.21E-04 | 2.99E-02 | -0.77 | 1.42E-01 | -2.22 | 3.38E-04 |
| LOC101929926 | 19.89 | 7.23E-04 | 2.99E-02 | -0.97 | 6.85E-02 | -2.08 | 7.01E-04 |
| AX748417 | 19.87 | 7.30E-04 | 3.01E-02 | -0.23 | 6.43E-01 | -2.49 | 7.54E-05 |
| TRIM32 | 19.81 | 7.47E-04 | 3.08E-02 | 1.38 | 1.37E-02 | 1.71 | 3.65E-03 |
| ACOX1 | 19.79 | 7.53E-04 | 3.09E-02 | 0.85 | 1.05E-01 | -2.15 | 4.80E-04 |
| NXPE1 | 19.79 | 7.53E-04 | 3.09E-02 | -0.87 | 9.81E-02 | -2.14 | 5.13E-04 |
| C9ORF173 | 19.78 | 7.57E-04 | 3.09E-02 | -1.23 | 2.53E-02 | -1.85 | 2.01E-03 |
| CEP89 | 19.77 | 7.59E-04 | 3.09E-02 | -0.47 | 3.51E-01 | -2.38 | 1.45E-04 |
| CEP162 | 19.77 | 7.59E-04 | 3.09E-02 | -0.66 | 2.01E-01 | -2.27 | 2.53E-04 |
| TTC7B | 19.74 | 7.72E-04 | 3.11E-02 | -1.56 | 6.29E-03 | -1.51 | 8.24E-03 |
| DFNA5 | 19.73 | 7.73E-04 | 3.11E-02 | 1.43 | 1.09E-02 | 1.64 | 4.78E-03 |
| NUDT18 | 19.74 | 7.69E-04 | 3.11E-02 | -1.14 | 3.54E-02 | -1.92 | 1.46E-03 |
| TRAV12-2 | 19.73 | 7.73E-04 | 3.11E-02 | -1.54 | 6.79E-03 | -1.53 | 7.65E-03 |
| FCER1A | 19.74 | 7.71E-04 | 3.11E-02 | 0.56 | 2.75E-01 | -2.33 | 1.88E-04 |
| FAM71A | 19.74 | 7.70E-04 | 3.11E-02 | -0.94 | 7.52E-02 | -2.08 | 6.87E-04 |
| NFKBIE | 19.72 | 7.75E-04 | 3.11E-02 | -0.92 | 8.31E-02 | -2.10 | 6.28E-04 |
| ZZEF1 | 19.71 | 7.79E-04 | 3.11E-02 | -1.59 | 5.66E-03 | -1.48 | 9.28E-03 |
| C9ORF62 | 19.71 | 7.79E-04 | 3.11E-02 | -0.98 | 6.71E-02 | -2.06 | 7.83E-04 |
| LOC101928844 | 19.68 | 7.87E-04 | 3.12E-02 | -1.38 | 1.34E-02 | -1.69 | 3.97E-03 |
| TFAP2C | 19.69 | 7.86E-04 | 3.12E-02 | -1.04 | 5.21E-02 | -2.00 | 1.02E-03 |
| LMNB1 | 19.69 | 7.86E-04 | 3.12E-02 | 1.72 | 3.24E-03 | 1.34 | 1.64E-02 |
| SMARCA4 | 19.69 | 7.84E-04 | 3.12E-02 | -0.98 | 6.65E-02 | -2.05 | 7.95E-04 |
| GPC4 | 19.67 | 7.90E-04 | 3.12E-02 | -1.36 | 1.48E-02 | -1.71 | 3.62E-03 |
| MYOZ2 | 19.64 | 8.02E-04 | 3.15E-02 | 1.58 | 5.96E-03 | 1.48 | 9.14E-03 |
| MAP7D1 | 19.64 | 8.02E-04 | 3.15E-02 | -1.87 | 1.63E-03 | -1.16 | 3.35E-02 |
| RENBP | 19.64 | 8.02E-04 | 3.15E-02 | -1.05 | 5.03E-02 | -1.99 | 1.08E-03 |
| TSC22D3 | 19.63 | 8.03E-04 | 3.15E-02 | -1.44 | 1.05E-02 | -1.62 | 5.21E-03 |
| WDR55 | 19.63 | 8.05E-04 | 3.15E-02 | -1.10 | 4.25E-02 | -1.95 | 1.29E-03 |
| CRB2 | 19.61 | 8.10E-04 | 3.15E-02 | -0.88 | 9.55E-02 | -2.12 | 5.76E-04 |
| SLC12A3 | 19.60 | 8.15E-04 | 3.15E-02 | 1.16 | 3.26E-02 | -1.89 | 1.70E-03 |
| TTLL13 | 19.61 | 8.13E-04 | 3.15E-02 | -0.87 | 9.86E-02 | -2.12 | 5.61E-04 |
| KIAA0020 | 19.61 | 8.13E-04 | 3.15E-02 | -0.94 | 7.78E-02 | -2.08 | 7.10E-04 |
| DMKN | 19.62 | 8.08E-04 | 3.15E-02 | 1.37 | 1.40E-02 | -1.69 | 3.92E-03 |
| NUB1 | 19.60 | 8.14E-04 | 3.15E-02 | -0.08 | 8.65E-01 | -2.52 | 6.40E-05 |
| RP11-38C18.3 | 19.59 | 8.19E-04 | 3.15E-02 | -1.55 | 6.54E-03 | -1.50 | 8.53E-03 |
| PHKG1 | 19.58 | 8.22E-04 | 3.16E-02 | -1.32 | 1.71E-02 | -1.73 | 3.28E-03 |
| GTPBP2 | 19.56 | 8.29E-04 | 3.18E-02 | -1.07 | 4.70E-02 | -1.96 | 1.21E-03 |
| RAB8B | 19.55 | 8.29E-04 | 3.18E-02 | -0.61 | 2.39E-01 | -2.29 | 2.38E-04 |
| RALGPS2 | 19.54 | 8.33E-04 | 3.18E-02 | 1.03 | 5.36E-02 | -1.99 | 1.06E-03 |
| VWA8-AS1 | 19.55 | 8.32E-04 | 3.18E-02 | 1.82 | 2.13E-03 | 1.22 | 2.68E-02 |
| URGCP | 19.54 | 8.35E-04 | 3.18E-02 | -1.05 | 5.03E-02 | -1.98 | 1.14E-03 |
| MASP1 | 19.52 | 8.40E-04 | 3.19E-02 | -1.37 | 1.37E-02 | -1.67 | 4.19E-03 |
| NF2 | 19.52 | 8.43E-04 | 3.19E-02 | -0.46 | 3.65E-01 | -2.36 | 1.58E-04 |
| LOC220077 | 19.51 | 8.44E-04 | 3.19E-02 | -0.18 | 7.16E-01 | -2.49 | 8.08E-05 |
| FHL5 | 19.51 | 8.48E-04 | 3.20E-02 | 1.33 | 1.66E-02 | 1.72 | 3.49E-03 |
| PDLIM3 | 19.50 | 8.52E-04 | 3.20E-02 | -1.76 | 2.67E-03 | -1.27 | 2.18E-02 |
| MYLK4 | 19.50 | 8.50E-04 | 3.20E-02 | -1.77 | 2.58E-03 | -1.26 | 2.26E-02 |
| CCL7 | 19.48 | 8.62E-04 | 3.23E-02 | 1.58 | 5.74E-03 | 1.45 | 1.03E-02 |
| CTD-2534I21.8 | 19.47 | 8.65E-04 | 3.23E-02 | -0.84 | 1.12E-01 | -2.14 | 5.30E-04 |
| RP13-30A9.2 | 19.47 | 8.65E-04 | 3.23E-02 | -0.88 | 9.59E-02 | -2.10 | 6.17E-04 |
| SIK2 | 19.47 | 8.67E-04 | 3.23E-02 | -1.15 | 3.44E-02 | -1.88 | 1.72E-03 |
| FOXM1 | 19.45 | 8.75E-04 | 3.26E-02 | -0.96 | 7.16E-02 | -2.04 | 8.35E-04 |
| PMEPA1 | 19.44 | 8.78E-04 | 3.26E-02 | -1.78 | 2.50E-03 | -1.24 | 2.40E-02 |
| E2F3 | 19.43 | 8.81E-04 | 3.26E-02 | -1.38 | 1.36E-02 | -1.66 | 4.42E-03 |
| ELMOD2 | 19.43 | 8.83E-04 | 3.26E-02 | 1.03 | 5.42E-02 | -1.98 | 1.11E-03 |
| AL832909 | 19.44 | 8.79E-04 | 3.26E-02 | -1.59 | 5.66E-03 | -1.45 | 1.06E-02 |
| ANKRD26P3 | 19.41 | 8.90E-04 | 3.28E-02 | -1.34 | 1.61E-02 | -1.70 | 3.78E-03 |
| FGF7 | 19.40 | 8.95E-04 | 3.28E-02 | 1.15 | 3.44E-02 | 1.87 | 1.79E-03 |
| ADCY10 | 19.40 | 8.94E-04 | 3.28E-02 | -0.88 | 9.62E-02 | -2.10 | 6.38E-04 |
| SH3PXD2A | 19.39 | 8.98E-04 | 3.29E-02 | 0.40 | 4.29E-01 | -2.38 | 1.44E-04 |
| MUC5B | 19.38 | 9.01E-04 | 3.29E-02 | -0.83 | 1.12E-01 | -2.13 | 5.50E-04 |
| CNPY3 | 19.38 | 8.99E-04 | 3.29E-02 | -0.79 | 1.32E-01 | -2.16 | 4.68E-04 |
| RYR3 | 19.37 | 9.04E-04 | 3.29E-02 | 1.58 | 5.77E-03 | 1.44 | 1.08E-02 |
| TOLLIP-AS1 | 19.37 | 9.05E-04 | 3.29E-02 | -1.22 | 2.57E-02 | -1.80 | 2.42E-03 |
| DISP1 | 19.36 | 9.10E-04 | 3.30E-02 | -1.38 | 1.37E-02 | -1.65 | 4.57E-03 |
| RBM41 | 19.36 | 9.13E-04 | 3.31E-02 | 1.52 | 7.44E-03 | 1.50 | 8.42E-03 |
| LPHN1 | 19.35 | 9.16E-04 | 3.31E-02 | -0.50 | 3.22E-01 | -2.32 | 1.96E-04 |
| NRIP3 | 19.34 | 9.18E-04 | 3.31E-02 | -1.15 | 3.35E-02 | -1.86 | 1.88E-03 |
| MTHFD1 | 19.32 | 9.27E-04 | 3.31E-02 | 1.48 | 8.76E-03 | 1.54 | 7.28E-03 |
| KIF3C | 19.33 | 9.25E-04 | 3.31E-02 | 1.36 | 1.44E-02 | 1.66 | 4.41E-03 |
| LOC101928525 | 19.31 | 9.29E-04 | 3.31E-02 | -0.94 | 7.58E-02 | -2.04 | 8.45E-04 |
| KCNJ11 | 19.33 | 9.25E-04 | 3.31E-02 | -1.10 | 4.10E-02 | -1.91 | 1.55E-03 |
| NLGN4X | 19.33 | 9.21E-04 | 3.31E-02 | -1.40 | 1.22E-02 | -1.62 | 5.18E-03 |
| LEPR | 19.32 | 9.27E-04 | 3.31E-02 | 1.02 | 5.66E-02 | 1.98 | 1.13E-03 |
| EIF5A | 19.27 | 9.42E-04 | 3.35E-02 | -1.45 | 1.02E-02 | -1.57 | 6.41E-03 |
| LOC100507535 | 19.27 | 9.45E-04 | 3.35E-02 | -1.19 | 2.96E-02 | -1.82 | 2.21E-03 |
| KCNQ5 | 19.24 | 9.56E-04 | 3.38E-02 | -1.69 | 3.71E-03 | -1.32 | 1.79E-02 |
| LINC00460 | 19.23 | 9.60E-04 | 3.38E-02 | 1.32 | 1.70E-02 | -1.69 | 3.94E-03 |
| RP11-309G3.3 | 19.23 | 9.58E-04 | 3.38E-02 | -1.60 | 5.44E-03 | -1.41 | 1.22E-02 |
| SIPA1L2 | 19.23 | 9.60E-04 | 3.38E-02 | -0.93 | 7.99E-02 | -2.04 | 8.35E-04 |
| ISCA2 | 19.21 | 9.68E-04 | 3.39E-02 | 1.43 | 1.10E-02 | 1.58 | 6.09E-03 |
| RAPGEFL1 | 19.22 | 9.67E-04 | 3.39E-02 | -1.23 | 2.52E-02 | -1.78 | 2.67E-03 |
| C1ORF53 | 19.21 | 9.69E-04 | 3.39E-02 | -0.99 | 6.26E-02 | -1.99 | 1.08E-03 |
| MAGEA9 | 19.20 | 9.71E-04 | 3.40E-02 | -1.49 | 8.55E-03 | -1.52 | 7.91E-03 |
| FAM110B | 19.18 | 9.79E-04 | 3.42E-02 | -0.79 | 1.29E-01 | -2.14 | 5.28E-04 |
| BCL11A | 19.17 | 9.84E-04 | 3.43E-02 | 0.42 | 4.10E-01 | -2.35 | 1.68E-04 |
| BRMS1 | 19.15 | 9.94E-04 | 3.43E-02 | -1.76 | 2.66E-03 | -1.22 | 2.61E-02 |
| BC033528 | 19.15 | 9.93E-04 | 3.43E-02 | -0.80 | 1.25E-01 | -2.12 | 5.56E-04 |
| RGS10 | 19.16 | 9.89E-04 | 3.43E-02 | -1.57 | 6.18E-03 | -1.43 | 1.12E-02 |
| TAOK2 | 19.15 | 9.93E-04 | 3.43E-02 | -0.57 | 2.63E-01 | -2.27 | 2.64E-04 |
| NFE2 | 19.16 | 9.87E-04 | 3.43E-02 | -0.81 | 1.21E-01 | -2.12 | 5.69E-04 |
| APRT | 19.14 | 9.97E-04 | 3.43E-02 | -0.94 | 7.54E-02 | -2.02 | 9.25E-04 |
| PDHA1 | 19.13 | 1.00E-03 | 3.44E-02 | 1.63 | 4.63E-03 | 1.36 | 1.52E-02 |
| COCH | 19.11 | 1.01E-03 | 3.47E-02 | 1.15 | 3.48E-02 | 1.84 | 2.04E-03 |
| LOC100505902 | 19.10 | 1.02E-03 | 3.49E-02 | 1.69 | 3.63E-03 | 1.29 | 1.97E-02 |
| EHBP1L1 | 19.08 | 1.02E-03 | 3.49E-02 | -1.37 | 1.37E-02 | -1.62 | 5.22E-03 |
| INTS1 | 19.08 | 1.02E-03 | 3.49E-02 | -0.82 | 1.18E-01 | -2.11 | 6.10E-04 |
| MYL5 | 19.07 | 1.03E-03 | 3.50E-02 | -1.49 | 8.63E-03 | -1.50 | 8.38E-03 |
| TSPAN17 | 19.07 | 1.03E-03 | 3.50E-02 | -1.39 | 1.28E-02 | -1.60 | 5.62E-03 |
| PLCG1 | 19.05 | 1.04E-03 | 3.52E-02 | -1.06 | 4.88E-02 | -1.92 | 1.50E-03 |
| RNASEH1 | 19.05 | 1.04E-03 | 3.52E-02 | -1.42 | 1.14E-02 | -1.57 | 6.36E-03 |
| MEPCE | 19.05 | 1.04E-03 | 3.52E-02 | -1.53 | 7.22E-03 | -1.46 | 1.01E-02 |
| OGFRL1 | 19.02 | 1.05E-03 | 3.54E-02 | -1.43 | 1.07E-02 | -1.55 | 6.91E-03 |
| LOC284837 | 19.03 | 1.05E-03 | 3.54E-02 | -1.14 | 3.51E-02 | -1.84 | 2.11E-03 |
| COL18A1-AS1 | 19.03 | 1.05E-03 | 3.54E-02 | -0.96 | 7.21E-02 | -2.00 | 1.02E-03 |
| PACSIN2 | 19.00 | 1.06E-03 | 3.56E-02 | -1.01 | 5.82E-02 | -1.95 | 1.29E-03 |
| MAPKBP1 | 18.98 | 1.07E-03 | 3.56E-02 | -1.27 | 2.08E-02 | -1.71 | 3.64E-03 |
| MTMR11 | 18.99 | 1.07E-03 | 3.56E-02 | 1.71 | 3.43E-03 | 1.27 | 2.20E-02 |
| SLC4A3 | 19.00 | 1.06E-03 | 3.56E-02 | -0.95 | 7.34E-02 | -2.00 | 1.02E-03 |
| CAPZA1 | 18.98 | 1.07E-03 | 3.56E-02 | -1.58 | 5.84E-03 | -1.40 | 1.29E-02 |
| HCG11 | 18.99 | 1.07E-03 | 3.56E-02 | -1.32 | 1.72E-02 | -1.66 | 4.36E-03 |
| FGFR1 | 18.97 | 1.08E-03 | 3.57E-02 | -0.78 | 1.35E-01 | -2.12 | 5.61E-04 |
| LINC01197 | 18.98 | 1.07E-03 | 3.57E-02 | -0.90 | 8.99E-02 | -2.04 | 8.43E-04 |
| AQP4 | 18.95 | 1.09E-03 | 3.60E-02 | 1.40 | 1.26E-02 | 1.58 | 6.10E-03 |
| ASB16 | 18.95 | 1.09E-03 | 3.60E-02 | -1.54 | 7.00E-03 | -1.44 | 1.10E-02 |
| DCXR | 18.94 | 1.10E-03 | 3.61E-02 | 1.84 | 1.89E-03 | 1.11 | 4.09E-02 |
| EPHA5-AS1 | 18.93 | 1.10E-03 | 3.61E-02 | -1.34 | 1.60E-02 | -1.64 | 4.86E-03 |
| DOCK7 | 18.93 | 1.10E-03 | 3.61E-02 | 1.31 | 1.82E-02 | 1.67 | 4.27E-03 |
| DUSP3 | 18.91 | 1.10E-03 | 3.62E-02 | -1.30 | 1.85E-02 | -1.67 | 4.22E-03 |
| PTPRN2 | 18.90 | 1.11E-03 | 3.64E-02 | 0.77 | 1.42E-01 | -2.12 | 5.55E-04 |
| CD52 | 18.87 | 1.12E-03 | 3.65E-02 | 1.71 | 3.37E-03 | 1.25 | 2.37E-02 |
| APOE | 18.87 | 1.13E-03 | 3.65E-02 | -0.49 | 3.39E-01 | -2.29 | 2.36E-04 |
| JUP | 18.87 | 1.13E-03 | 3.65E-02 | -0.94 | 7.53E-02 | -1.99 | 1.06E-03 |
| KCNK4 | 18.88 | 1.12E-03 | 3.65E-02 | -1.07 | 4.62E-02 | -1.88 | 1.72E-03 |
| SCARF2 | 18.88 | 1.12E-03 | 3.65E-02 | -0.40 | 4.25E-01 | -2.33 | 1.87E-04 |
| PTPRS | 18.87 | 1.12E-03 | 3.65E-02 | 1.08 | 4.53E-02 | -1.88 | 1.76E-03 |
| SPPL3 | 18.87 | 1.13E-03 | 3.65E-02 | -0.35 | 4.92E-01 | -2.36 | 1.63E-04 |
| VTI1A | 18.86 | 1.13E-03 | 3.65E-02 | 0.74 | 1.55E-01 | -2.14 | 5.18E-04 |
| PRKAG3 | 18.85 | 1.13E-03 | 3.65E-02 | -1.77 | 2.59E-03 | -1.18 | 3.10E-02 |
| SSU72P8 | 18.84 | 1.14E-03 | 3.65E-02 | 0.18 | 7.21E-01 | -2.43 | 1.12E-04 |
| ANGPTL1 | 18.85 | 1.14E-03 | 3.65E-02 | 1.18 | 3.04E-02 | 1.78 | 2.66E-03 |
| HFE | 18.81 | 1.15E-03 | 3.70E-02 | -0.47 | 3.54E-01 | -2.29 | 2.33E-04 |
| PSMB7 | 18.80 | 1.16E-03 | 3.71E-02 | 1.47 | 9.34E-03 | 1.49 | 8.87E-03 |
| MCUR1 | 18.78 | 1.17E-03 | 3.72E-02 | 1.02 | 5.76E-02 | 1.92 | 1.45E-03 |
| IGLJ3 | 18.79 | 1.17E-03 | 3.72E-02 | -0.54 | 2.91E-01 | -2.25 | 2.87E-04 |
| GXYLT1 | 18.78 | 1.17E-03 | 3.72E-02 | -1.13 | 3.63E-02 | -1.82 | 2.30E-03 |
| TMEM53 | 18.78 | 1.17E-03 | 3.72E-02 | 1.05 | 4.98E-02 | -1.89 | 1.68E-03 |
| RHBDF1 | 18.78 | 1.17E-03 | 3.72E-02 | -0.49 | 3.37E-01 | -2.28 | 2.48E-04 |
| GPALPP1 | 18.77 | 1.17E-03 | 3.72E-02 | 1.05 | 5.09E-02 | 1.89 | 1.65E-03 |
| STBD1 | 18.76 | 1.18E-03 | 3.73E-02 | -1.33 | 1.68E-02 | -1.63 | 5.04E-03 |
| ARHGEF11 | 18.75 | 1.18E-03 | 3.73E-02 | 0.49 | 3.37E-01 | -2.27 | 2.52E-04 |
| CD81-AS1 | 18.74 | 1.18E-03 | 3.73E-02 | -1.29 | 1.93E-02 | -1.66 | 4.40E-03 |
| GAL | 18.73 | 1.19E-03 | 3.74E-02 | -1.07 | 4.69E-02 | -1.87 | 1.82E-03 |
| LOC101926959 | 18.73 | 1.19E-03 | 3.74E-02 | -1.06 | 4.88E-02 | -1.88 | 1.75E-03 |
| SLC25A36 | 18.72 | 1.20E-03 | 3.74E-02 | 0.96 | 7.05E-02 | 1.96 | 1.22E-03 |
| LGALS9 | 18.72 | 1.20E-03 | 3.74E-02 | -1.38 | 1.35E-02 | -1.57 | 6.37E-03 |
| DCAF12L1 | 18.72 | 1.20E-03 | 3.74E-02 | -0.93 | 7.96E-02 | -1.99 | 1.08E-03 |
| LOC100131655 | 18.71 | 1.20E-03 | 3.74E-02 | -1.41 | 1.18E-02 | -1.54 | 7.35E-03 |
| PHPT1 | 18.69 | 1.21E-03 | 3.76E-02 | 1.33 | 1.68E-02 | 1.62 | 5.20E-03 |
| OBSCN | 18.69 | 1.21E-03 | 3.76E-02 | -1.07 | 4.67E-02 | -1.86 | 1.87E-03 |
| SLC25A22 | 18.69 | 1.21E-03 | 3.76E-02 | -1.05 | 5.05E-02 | -1.88 | 1.73E-03 |
| ANKRD33B | 18.67 | 1.22E-03 | 3.76E-02 | 1.72 | 3.18E-03 | 1.21 | 2.78E-02 |
| LOC101930235 | 18.68 | 1.22E-03 | 3.76E-02 | 1.47 | 9.19E-03 | 1.47 | 9.56E-03 |
| DUSP8 | 18.68 | 1.22E-03 | 3.76E-02 | -0.90 | 8.73E-02 | -2.00 | 1.01E-03 |
| HSFX1 | 18.67 | 1.22E-03 | 3.76E-02 | -0.67 | 1.96E-01 | -2.17 | 4.50E-04 |
| TSPAN16 | 18.67 | 1.22E-03 | 3.76E-02 | -0.36 | 4.73E-01 | -2.33 | 1.87E-04 |
| LOC101929504 | 18.66 | 1.22E-03 | 3.76E-02 | -1.39 | 1.30E-02 | -1.55 | 6.80E-03 |
| LOC101928748 | 18.65 | 1.23E-03 | 3.77E-02 | -0.66 | 2.01E-01 | -2.17 | 4.42E-04 |
| PHB | 18.63 | 1.24E-03 | 3.79E-02 | 1.58 | 5.83E-03 | 1.35 | 1.55E-02 |
| SMTN | 18.62 | 1.25E-03 | 3.79E-02 | -1.63 | 4.72E-03 | -1.30 | 1.92E-02 |
| RNF4 | 18.62 | 1.25E-03 | 3.79E-02 | -1.01 | 5.87E-02 | -1.91 | 1.54E-03 |
| CTNNBL1 | 18.61 | 1.25E-03 | 3.79E-02 | -1.00 | 6.18E-02 | -1.92 | 1.47E-03 |
| NAA16 | 18.60 | 1.25E-03 | 3.79E-02 | -0.13 | 7.99E-01 | -2.43 | 1.14E-04 |
| PTPN6 | 18.63 | 1.24E-03 | 3.79E-02 | -0.70 | 1.75E-01 | -2.14 | 5.15E-04 |
| SLC9A7 | 18.61 | 1.25E-03 | 3.79E-02 | 0.36 | 4.71E-01 | -2.33 | 1.93E-04 |
| HCRTR2 | 18.60 | 1.25E-03 | 3.79E-02 | -0.39 | 4.35E-01 | -2.31 | 2.10E-04 |
| PKMYT1 | 18.58 | 1.27E-03 | 3.80E-02 | -1.14 | 3.57E-02 | -1.79 | 2.59E-03 |
| OMA1 | 18.57 | 1.27E-03 | 3.80E-02 | 1.23 | 2.49E-02 | 1.70 | 3.72E-03 |
| ITGA7 | 18.57 | 1.27E-03 | 3.80E-02 | -1.22 | 2.57E-02 | -1.71 | 3.60E-03 |
| LRRC41 | 18.59 | 1.26E-03 | 3.80E-02 | 0.72 | 1.65E-01 | -2.12 | 5.58E-04 |
| ZNF383 | 18.58 | 1.27E-03 | 3.80E-02 | 1.21 | 2.67E-02 | 1.72 | 3.46E-03 |
| XAGE3 | 18.58 | 1.27E-03 | 3.80E-02 | -0.81 | 1.20E-01 | -2.06 | 7.67E-04 |
| DRD4 | 18.59 | 1.26E-03 | 3.80E-02 | 1.91 | 1.35E-03 | 0.97 | 6.80E-02 |
| CSH1 | 18.58 | 1.27E-03 | 3.80E-02 | -0.55 | 2.80E-01 | -2.23 | 3.30E-04 |
| JUND | 18.60 | 1.26E-03 | 3.80E-02 | -0.68 | 1.88E-01 | -2.15 | 4.86E-04 |
| RAB3GAP1 | 18.56 | 1.28E-03 | 3.81E-02 | -1.37 | 1.42E-02 | -1.56 | 6.54E-03 |
| GAPDHS | 18.55 | 1.29E-03 | 3.82E-02 | -1.47 | 9.07E-03 | -1.45 | 1.04E-02 |
| POLA1 | 18.55 | 1.29E-03 | 3.82E-02 | 1.35 | 1.51E-02 | 1.58 | 6.21E-03 |
| SSX2IP | 18.54 | 1.29E-03 | 3.82E-02 | 0.16 | 7.44E-01 | -2.40 | 1.27E-04 |
| PDGFRA | 18.54 | 1.29E-03 | 3.82E-02 | -0.69 | 1.81E-01 | -2.14 | 5.21E-04 |
| INF2 | 18.53 | 1.29E-03 | 3.83E-02 | -0.91 | 8.55E-02 | -1.98 | 1.11E-03 |
| PNMA1 | 18.53 | 1.30E-03 | 3.83E-02 | -1.51 | 7.96E-03 | -1.42 | 1.19E-02 |
| NLGN2 | 18.52 | 1.30E-03 | 3.84E-02 | 1.23 | 2.50E-02 | -1.69 | 3.81E-03 |
| B3GAT2 | 18.51 | 1.30E-03 | 3.84E-02 | 1.32 | 1.71E-02 | -1.60 | 5.59E-03 |
| IL6 | 18.50 | 1.31E-03 | 3.85E-02 | -1.02 | 5.67E-02 | -1.89 | 1.69E-03 |
| COMP | 18.49 | 1.32E-03 | 3.85E-02 | -1.04 | 5.22E-02 | -1.87 | 1.85E-03 |
| ISLR2 | 18.49 | 1.32E-03 | 3.85E-02 | -0.93 | 7.90E-02 | -1.96 | 1.22E-03 |
| LOC101928198 | 18.49 | 1.32E-03 | 3.85E-02 | -1.01 | 5.94E-02 | -1.90 | 1.62E-03 |
| TRIML2 | 18.48 | 1.33E-03 | 3.87E-02 | -0.84 | 1.08E-01 | -2.03 | 9.01E-04 |
| SH3KBP1 | 18.45 | 1.35E-03 | 3.92E-02 | 1.56 | 6.31E-03 | 1.35 | 1.57E-02 |
| MT2A | 18.43 | 1.35E-03 | 3.93E-02 | 1.31 | 1.80E-02 | 1.60 | 5.52E-03 |
| MYBPC2 | 18.43 | 1.36E-03 | 3.94E-02 | -1.76 | 2.68E-03 | -1.13 | 3.73E-02 |
| GLRA2 | 18.42 | 1.36E-03 | 3.94E-02 | -1.30 | 1.89E-02 | -1.62 | 5.27E-03 |
| SKI | 18.42 | 1.36E-03 | 3.94E-02 | 1.14 | 3.59E-02 | -1.77 | 2.78E-03 |
| SERPINA5 | 18.40 | 1.38E-03 | 3.95E-02 | -1.25 | 2.28E-02 | -1.66 | 4.44E-03 |
| CTB-176F20.3 | 18.40 | 1.38E-03 | 3.95E-02 | -1.53 | 7.33E-03 | -1.38 | 1.38E-02 |
| HNF1A | 18.41 | 1.37E-03 | 3.95E-02 | -0.75 | 1.52E-01 | -2.09 | 6.63E-04 |
| ATP8B5P | 18.41 | 1.37E-03 | 3.95E-02 | -0.64 | 2.17E-01 | -2.16 | 4.65E-04 |
| ALK | 18.41 | 1.37E-03 | 3.95E-02 | -1.12 | 3.82E-02 | -1.78 | 2.64E-03 |
| AP001347.6 | 18.38 | 1.39E-03 | 3.97E-02 | 1.62 | 4.84E-03 | 1.28 | 2.11E-02 |
| CHST14 | 18.38 | 1.39E-03 | 3.98E-02 | -1.18 | 3.09E-02 | -1.73 | 3.31E-03 |
| GYPB | 18.36 | 1.40E-03 | 3.99E-02 | 0.31 | 5.38E-01 | -2.33 | 1.91E-04 |
| SHPK | 18.37 | 1.40E-03 | 3.99E-02 | 0.48 | 3.45E-01 | -2.24 | 2.98E-04 |
| LOC100505718 | 18.37 | 1.40E-03 | 3.99E-02 | -0.88 | 9.56E-02 | -1.99 | 1.07E-03 |
| PROS1 | 18.36 | 1.40E-03 | 3.99E-02 | -1.25 | 2.28E-02 | -1.65 | 4.52E-03 |
| RPP40 | 18.33 | 1.42E-03 | 4.03E-02 | -1.36 | 1.44E-02 | -1.54 | 7.26E-03 |
| KRT8P12 | 18.33 | 1.42E-03 | 4.03E-02 | 1.62 | 4.84E-03 | -1.27 | 2.16E-02 |
| LYG2 | 18.33 | 1.42E-03 | 4.03E-02 | -1.12 | 3.85E-02 | -1.78 | 2.72E-03 |
| NAA11 | 18.32 | 1.43E-03 | 4.03E-02 | -1.09 | 4.38E-02 | -1.81 | 2.40E-03 |
| ENTPD3 | 18.31 | 1.43E-03 | 4.03E-02 | -1.36 | 1.49E-02 | -1.55 | 7.08E-03 |
| SLC1A1 | 18.32 | 1.43E-03 | 4.03E-02 | 0.79 | 1.32E-01 | -2.05 | 7.98E-04 |
| SPARC | 18.31 | 1.44E-03 | 4.04E-02 | -1.73 | 3.06E-03 | -1.15 | 3.47E-02 |
| HMGCR | 18.31 | 1.43E-03 | 4.04E-02 | 0.99 | 6.31E-02 | -1.89 | 1.68E-03 |
| FBXL7 | 18.29 | 1.44E-03 | 4.05E-02 | -1.17 | 3.11E-02 | -1.72 | 3.43E-03 |
| OIP5 | 18.29 | 1.45E-03 | 4.05E-02 | -1.67 | 3.93E-03 | -1.21 | 2.72E-02 |
| CELF1 | 18.29 | 1.45E-03 | 4.05E-02 | -1.12 | 3.90E-02 | -1.77 | 2.74E-03 |
| TST | 18.28 | 1.45E-03 | 4.05E-02 | 1.42 | 1.11E-02 | 1.47 | 9.63E-03 |
| C17ORF70 | 18.28 | 1.45E-03 | 4.05E-02 | -0.52 | 3.05E-01 | -2.22 | 3.51E-04 |
| SEC14L4 | 18.26 | 1.47E-03 | 4.09E-02 | 1.12 | 3.91E-02 | -1.77 | 2.77E-03 |
| C1R | 18.24 | 1.47E-03 | 4.10E-02 | 1.40 | 1.22E-02 | 1.49 | 8.92E-03 |
| RAB32 | 18.25 | 1.47E-03 | 4.10E-02 | -1.16 | 3.32E-02 | -1.73 | 3.29E-03 |
| LINC01278 | 18.24 | 1.48E-03 | 4.10E-02 | 1.53 | 7.15E-03 | 1.36 | 1.53E-02 |
| MAP2K2 | 18.23 | 1.49E-03 | 4.10E-02 | -1.28 | 2.02E-02 | -1.61 | 5.44E-03 |
| HMGB1P12 | 18.23 | 1.49E-03 | 4.10E-02 | 1.31 | 1.79E-02 | 1.58 | 6.16E-03 |
| FAM135A | 18.23 | 1.48E-03 | 4.10E-02 | 0.95 | 7.29E-02 | 1.91 | 1.51E-03 |
| PLCB2 | 18.22 | 1.49E-03 | 4.11E-02 | -1.11 | 3.97E-02 | -1.77 | 2.78E-03 |
| DICER1-AS1 | 18.22 | 1.49E-03 | 4.11E-02 | -0.77 | 1.42E-01 | -2.06 | 7.80E-04 |
| MRPS22 | 18.21 | 1.50E-03 | 4.12E-02 | 1.34 | 1.59E-02 | 1.55 | 6.98E-03 |
| CMTM3 | 18.19 | 1.51E-03 | 4.14E-02 | -0.40 | 4.29E-01 | -2.27 | 2.62E-04 |
| WHSC1 | 18.17 | 1.52E-03 | 4.17E-02 | 0.61 | 2.37E-01 | -2.16 | 4.79E-04 |
| PRPH2 | 18.17 | 1.52E-03 | 4.17E-02 | -1.35 | 1.49E-02 | -1.53 | 7.60E-03 |
| FCGR3A | 18.15 | 1.54E-03 | 4.20E-02 | -1.39 | 1.31E-02 | -1.49 | 8.75E-03 |
| SLC41A1 | 18.13 | 1.54E-03 | 4.20E-02 | 1.46 | 9.71E-03 | 1.42 | 1.19E-02 |
| LINC00452 | 18.14 | 1.54E-03 | 4.20E-02 | -1.08 | 4.58E-02 | -1.80 | 2.52E-03 |
| DYNC1I1 | 18.13 | 1.55E-03 | 4.20E-02 | -0.68 | 1.87E-01 | -2.10 | 6.20E-04 |
| SPATA21 | 18.13 | 1.55E-03 | 4.20E-02 | -0.88 | 9.39E-02 | -1.96 | 1.23E-03 |
| LOC101929125 | 18.13 | 1.54E-03 | 4.20E-02 | -0.57 | 2.63E-01 | -2.17 | 4.40E-04 |
| MAP3K6 | 18.12 | 1.55E-03 | 4.20E-02 | 0.81 | 1.21E-01 | 2.01 | 9.60E-04 |
| GFI1B | 18.12 | 1.56E-03 | 4.21E-02 | -1.01 | 5.93E-02 | -1.85 | 1.96E-03 |
| TNNI2 | 18.10 | 1.57E-03 | 4.22E-02 | -1.80 | 2.24E-03 | -1.04 | 5.24E-02 |
| IGLL5 | 18.10 | 1.57E-03 | 4.22E-02 | -1.33 | 1.68E-02 | -1.55 | 6.99E-03 |
| ADAMTS19 | 18.10 | 1.57E-03 | 4.22E-02 | -0.89 | 9.05E-02 | -1.95 | 1.30E-03 |
| C19ORF53 | 18.09 | 1.57E-03 | 4.22E-02 | 1.23 | 2.47E-02 | 1.64 | 4.78E-03 |
| MYL4 | 18.07 | 1.59E-03 | 4.26E-02 | -0.60 | 2.44E-01 | -2.15 | 4.88E-04 |
| RABEP1 | 18.05 | 1.60E-03 | 4.30E-02 | -0.92 | 8.09E-02 | -1.92 | 1.49E-03 |
| GPX2 | 18.04 | 1.61E-03 | 4.31E-02 | 1.36 | 1.47E-02 | 1.51 | 8.22E-03 |
| SERPINB5 | 18.04 | 1.61E-03 | 4.31E-02 | -0.79 | 1.29E-01 | -2.02 | 9.40E-04 |
| ZC3HAV1 | 18.02 | 1.62E-03 | 4.33E-02 | 1.56 | 6.37E-03 | 1.30 | 1.92E-02 |
| PACSIN1 | 18.02 | 1.62E-03 | 4.33E-02 | -0.73 | 1.62E-01 | -2.07 | 7.54E-04 |
| TTC18 | 17.98 | 1.66E-03 | 4.34E-02 | 1.26 | 2.22E-02 | 1.60 | 5.61E-03 |
| CTC-428G20.3 | 17.97 | 1.66E-03 | 4.34E-02 | -0.85 | 1.06E-01 | -1.97 | 1.18E-03 |
| SLC16A1 | 17.98 | 1.65E-03 | 4.34E-02 | 1.15 | 3.44E-02 | 1.71 | 3.63E-03 |
| CST3 | 17.98 | 1.65E-03 | 4.34E-02 | 1.23 | 2.49E-02 | 1.63 | 5.00E-03 |
| SHKBP1 | 18.00 | 1.64E-03 | 4.34E-02 | -1.02 | 5.70E-02 | -1.83 | 2.17E-03 |
| PAFAH1B3 | 18.00 | 1.64E-03 | 4.34E-02 | -0.69 | 1.85E-01 | -2.09 | 6.67E-04 |
| GPC1 | 17.99 | 1.64E-03 | 4.34E-02 | -0.58 | 2.58E-01 | -2.15 | 4.82E-04 |
| SLC5A4 | 17.98 | 1.66E-03 | 4.34E-02 | -1.22 | 2.62E-02 | -1.64 | 4.78E-03 |
| PHF7 | 18.00 | 1.64E-03 | 4.34E-02 | 0.49 | 3.34E-01 | -2.21 | 3.70E-04 |
| RARRES1 | 17.98 | 1.65E-03 | 4.34E-02 | 0.27 | 5.93E-01 | -2.31 | 2.10E-04 |
| NCF4 | 17.97 | 1.66E-03 | 4.34E-02 | -0.98 | 6.64E-02 | -1.86 | 1.88E-03 |
| BC041347 | 17.98 | 1.65E-03 | 4.34E-02 | -0.53 | 3.02E-01 | -2.18 | 4.12E-04 |
| LECT2 | 17.97 | 1.66E-03 | 4.34E-02 | 1.42 | 1.12E-02 | 1.43 | 1.11E-02 |
| POLE2 | 17.96 | 1.67E-03 | 4.36E-02 | -1.13 | 3.63E-02 | -1.72 | 3.46E-03 |
| PGK1 | 17.94 | 1.68E-03 | 4.38E-02 | -1.52 | 7.45E-03 | -1.33 | 1.70E-02 |
| LOC646482 | 17.93 | 1.69E-03 | 4.39E-02 | 1.62 | 4.92E-03 | 1.22 | 2.59E-02 |
| GPR78 | 17.93 | 1.69E-03 | 4.39E-02 | -1.22 | 2.60E-02 | -1.63 | 4.91E-03 |
| PAF1 | 17.92 | 1.70E-03 | 4.41E-02 | -1.11 | 3.99E-02 | -1.74 | 3.22E-03 |
| GEMIN7 | 17.90 | 1.71E-03 | 4.42E-02 | 1.39 | 1.28E-02 | 1.46 | 1.01E-02 |
| RARS | 17.90 | 1.71E-03 | 4.42E-02 | 1.13 | 3.76E-02 | 1.72 | 3.45E-03 |
| DUSP21 | 17.91 | 1.70E-03 | 4.42E-02 | -0.93 | 7.89E-02 | -1.89 | 1.64E-03 |
| BIK | 17.88 | 1.73E-03 | 4.45E-02 | -1.20 | 2.77E-02 | -1.64 | 4.72E-03 |
| MCPH1 | 17.88 | 1.73E-03 | 4.45E-02 | 1.06 | 4.79E-02 | 1.77 | 2.74E-03 |
| CPNE6 | 17.88 | 1.72E-03 | 4.45E-02 | -0.59 | 2.52E-01 | -2.14 | 5.19E-04 |
| FGFR1OP2 | 17.88 | 1.73E-03 | 4.45E-02 | 1.28 | 2.05E-02 | 1.57 | 6.39E-03 |
| CELF3 | 17.86 | 1.74E-03 | 4.45E-02 | 1.44 | 1.05E-02 | 1.40 | 1.26E-02 |
| KCNG2 | 17.87 | 1.73E-03 | 4.45E-02 | -0.31 | 5.32E-01 | -2.28 | 2.47E-04 |
| FAM167A | 17.87 | 1.74E-03 | 4.45E-02 | -1.01 | 6.00E-02 | -1.83 | 2.20E-03 |
| KRT15 | 17.86 | 1.74E-03 | 4.45E-02 | -0.10 | 8.43E-01 | -2.36 | 1.57E-04 |
| AC007787.2 | 17.85 | 1.75E-03 | 4.45E-02 | -1.19 | 2.90E-02 | -1.65 | 4.59E-03 |
| CACNG4 | 17.84 | 1.76E-03 | 4.45E-02 | 1.12 | 3.84E-02 | -1.72 | 3.49E-03 |
| PCDHB12 | 17.85 | 1.75E-03 | 4.45E-02 | -0.59 | 2.48E-01 | -2.13 | 5.35E-04 |
| WDR5 | 17.84 | 1.76E-03 | 4.45E-02 | -0.48 | 3.48E-01 | -2.20 | 3.85E-04 |
| CCDC108 | 17.85 | 1.75E-03 | 4.45E-02 | -0.34 | 4.98E-01 | -2.26 | 2.67E-04 |
| COL9A3 | 17.84 | 1.75E-03 | 4.45E-02 | -0.97 | 6.81E-02 | -1.85 | 1.96E-03 |
| ABTB1 | 17.84 | 1.75E-03 | 4.45E-02 | -0.51 | 3.17E-01 | -2.18 | 4.21E-04 |
| SLC37A1 | 17.83 | 1.76E-03 | 4.46E-02 | -0.25 | 6.17E-01 | -2.30 | 2.18E-04 |
| EFCAB12 | 17.83 | 1.76E-03 | 4.46E-02 | -0.05 | 9.17E-01 | -2.37 | 1.47E-04 |
| PKIG | 17.82 | 1.77E-03 | 4.46E-02 | 1.06 | 4.93E-02 | 1.77 | 2.73E-03 |
| LINC00964 | 17.82 | 1.77E-03 | 4.46E-02 | -0.82 | 1.16E-01 | -1.97 | 1.16E-03 |
| LOC145837 | 17.82 | 1.77E-03 | 4.46E-02 | -0.48 | 3.43E-01 | -2.19 | 3.94E-04 |
| LRRC2 | 17.81 | 1.77E-03 | 4.46E-02 | 1.39 | 1.29E-02 | 1.45 | 1.05E-02 |
| SIDT2 | 17.79 | 1.79E-03 | 4.47E-02 | -1.07 | 4.64E-02 | -1.76 | 2.96E-03 |
| SDCCAG8 | 17.80 | 1.78E-03 | 4.47E-02 | -1.63 | 4.63E-03 | -1.19 | 2.95E-02 |
| MSL3 | 17.79 | 1.79E-03 | 4.47E-02 | 0.61 | 2.32E-01 | -2.11 | 5.92E-04 |
| CELA3B | 17.79 | 1.79E-03 | 4.47E-02 | 0.00 | 9.98E-01 | -2.39 | 1.38E-04 |
| NLRP14 | 17.79 | 1.79E-03 | 4.47E-02 | -0.97 | 6.90E-02 | -1.85 | 1.99E-03 |
| LOC100996671 | 17.79 | 1.79E-03 | 4.47E-02 | 1.58 | 5.84E-03 | 1.25 | 2.35E-02 |
| PPP2R5D | 17.77 | 1.80E-03 | 4.49E-02 | -1.11 | 4.01E-02 | -1.72 | 3.45E-03 |
| WIZ | 17.76 | 1.81E-03 | 4.49E-02 | 1.00 | 6.16E-02 | -1.82 | 2.25E-03 |
| RPL39L | 17.77 | 1.81E-03 | 4.49E-02 | -1.02 | 5.64E-02 | -1.80 | 2.46E-03 |
| KNDC1 | 17.77 | 1.81E-03 | 4.49E-02 | -0.38 | 4.46E-01 | -2.24 | 3.11E-04 |
| LOC101929050 | 17.76 | 1.81E-03 | 4.49E-02 | 1.74 | 2.92E-03 | 1.07 | 4.77E-02 |
| LOC101927950 | 17.74 | 1.83E-03 | 4.50E-02 | 1.03 | 5.45E-02 | 1.79 | 2.58E-03 |
| HPS4 | 17.74 | 1.83E-03 | 4.50E-02 | -2.32 | 1.59E-04 | 0.07 | 8.84E-01 |
| CHRM3 | 17.74 | 1.83E-03 | 4.50E-02 | -0.94 | 7.53E-02 | -1.86 | 1.87E-03 |
| PI3 | 17.74 | 1.83E-03 | 4.50E-02 | -0.67 | 1.97E-01 | -2.08 | 7.13E-04 |
| CALML6 | 17.72 | 1.84E-03 | 4.51E-02 | -1.69 | 3.63E-03 | -1.12 | 3.91E-02 |
| ABCC9 | 17.73 | 1.83E-03 | 4.51E-02 | -1.29 | 1.92E-02 | -1.54 | 7.37E-03 |
| HCN4 | 17.72 | 1.84E-03 | 4.52E-02 | -0.36 | 4.72E-01 | -2.24 | 3.01E-04 |
| FGF17 | 17.71 | 1.84E-03 | 4.52E-02 | 1.47 | 9.07E-03 | 1.35 | 1.57E-02 |
| HBB | 17.71 | 1.85E-03 | 4.52E-02 | -1.81 | 2.19E-03 | -0.98 | 6.52E-02 |
| FBXL19-AS1 | 17.70 | 1.85E-03 | 4.52E-02 | -1.43 | 1.08E-02 | -1.39 | 1.32E-02 |
| BC015774 | 17.70 | 1.85E-03 | 4.52E-02 | 0.94 | 7.69E-02 | 1.86 | 1.86E-03 |
| LOC100507600 | 17.70 | 1.85E-03 | 4.52E-02 | -0.61 | 2.35E-01 | -2.11 | 6.11E-04 |
| SPSB2 | 17.69 | 1.86E-03 | 4.53E-02 | -0.65 | 2.08E-01 | -2.08 | 6.94E-04 |
| ST6GAL2 | 17.69 | 1.86E-03 | 4.53E-02 | 1.38 | 1.32E-02 | 1.44 | 1.09E-02 |
| WWTR1-AS1 | 17.69 | 1.87E-03 | 4.53E-02 | -0.92 | 8.22E-02 | -1.88 | 1.76E-03 |
| MMP28 | 17.68 | 1.87E-03 | 4.54E-02 | -0.37 | 4.64E-01 | -2.24 | 3.12E-04 |
| EPB41L4A-AS1 | 17.67 | 1.88E-03 | 4.55E-02 | -1.51 | 7.85E-03 | -1.31 | 1.85E-02 |
| TSPAN12 | 17.66 | 1.89E-03 | 4.56E-02 | 1.06 | 4.94E-02 | 1.76 | 2.95E-03 |
| RP11-862L9.3 | 17.65 | 1.90E-03 | 4.58E-02 | -1.08 | 4.49E-02 | -1.73 | 3.27E-03 |
| OARD1 | 17.64 | 1.90E-03 | 4.59E-02 | 1.26 | 2.20E-02 | 1.56 | 6.71E-03 |
| DLX3 | 17.64 | 1.90E-03 | 4.59E-02 | 1.42 | 1.13E-02 | 1.40 | 1.31E-02 |
| OSBPL6 | 17.63 | 1.91E-03 | 4.60E-02 | -1.54 | 7.01E-03 | -1.28 | 2.12E-02 |
| SMTNL2 | 17.62 | 1.93E-03 | 4.62E-02 | -1.68 | 3.81E-03 | -1.12 | 3.93E-02 |
| ZNF77 | 17.62 | 1.92E-03 | 4.62E-02 | -1.03 | 5.40E-02 | -1.77 | 2.77E-03 |
| TFAM | 17.61 | 1.93E-03 | 4.62E-02 | 1.20 | 2.84E-02 | 1.62 | 5.26E-03 |
| MCEE | 17.61 | 1.93E-03 | 4.63E-02 | 1.27 | 2.15E-02 | 1.55 | 7.00E-03 |
| CNGB3 | 17.60 | 1.94E-03 | 4.63E-02 | 1.23 | 2.45E-02 | 1.58 | 6.15E-03 |
| ATP13A1 | 17.60 | 1.94E-03 | 4.63E-02 | -1.07 | 4.70E-02 | -1.74 | 3.21E-03 |
| LRRN2 | 17.58 | 1.95E-03 | 4.65E-02 | -0.63 | 2.20E-01 | -2.08 | 6.90E-04 |
| CCDC22 | 17.59 | 1.95E-03 | 4.65E-02 | 0.55 | 2.82E-01 | -2.13 | 5.39E-04 |
| OR2I1P | 17.56 | 1.97E-03 | 4.68E-02 | 0.94 | 7.73E-02 | 1.85 | 1.99E-03 |
| LOC101928779 | 17.56 | 1.97E-03 | 4.68E-02 | -0.19 | 7.05E-01 | -2.30 | 2.18E-04 |
| SETD8 | 17.54 | 1.99E-03 | 4.71E-02 | -0.75 | 1.49E-01 | -2.00 | 1.04E-03 |
| PLEKHO1 | 17.54 | 1.99E-03 | 4.71E-02 | -1.32 | 1.74E-02 | -1.49 | 8.94E-03 |
| SNAI2 | 17.52 | 2.00E-03 | 4.74E-02 | 1.03 | 5.46E-02 | 1.76 | 2.87E-03 |
| CCDC69 | 17.46 | 2.06E-03 | 4.76E-02 | -1.60 | 5.48E-03 | -1.19 | 2.96E-02 |
| CDIPT | 17.45 | 2.06E-03 | 4.76E-02 | 1.36 | 1.44E-02 | 1.43 | 1.13E-02 |
| FAHD1 | 17.47 | 2.05E-03 | 4.76E-02 | 1.35 | 1.49E-02 | 1.44 | 1.08E-02 |
| LOC729680 | 17.47 | 2.05E-03 | 4.76E-02 | 1.41 | 1.19E-02 | 1.39 | 1.35E-02 |
| ZBTB2 | 17.45 | 2.07E-03 | 4.76E-02 | 1.19 | 2.96E-02 | 1.61 | 5.51E-03 |
| SPAG5-AS1 | 17.47 | 2.05E-03 | 4.76E-02 | 1.13 | 3.74E-02 | 1.67 | 4.30E-03 |
| PROM2 | 17.50 | 2.02E-03 | 4.76E-02 | -0.62 | 2.31E-01 | -2.08 | 6.86E-04 |
| HHIPL2 | 17.49 | 2.03E-03 | 4.76E-02 | -0.96 | 7.19E-02 | -1.82 | 2.21E-03 |
| SAMD10 | 17.47 | 2.05E-03 | 4.76E-02 | -0.57 | 2.65E-01 | -2.11 | 6.06E-04 |
| IFNW1 | 17.49 | 2.03E-03 | 4.76E-02 | 1.46 | 9.63E-03 | 1.34 | 1.65E-02 |
| ALDH1B1 | 17.47 | 2.05E-03 | 4.76E-02 | -0.77 | 1.41E-01 | -1.98 | 1.14E-03 |
| HOXB-AS1 | 17.47 | 2.05E-03 | 4.76E-02 | -0.71 | 1.71E-01 | -2.02 | 9.38E-04 |
| TBC1D16 | 17.51 | 2.02E-03 | 4.76E-02 | -0.74 | 1.53E-01 | -2.00 | 1.03E-03 |
| SOGA1 | 17.46 | 2.06E-03 | 4.76E-02 | -0.58 | 2.55E-01 | -2.10 | 6.34E-04 |
| SHANK1 | 17.47 | 2.05E-03 | 4.76E-02 | -0.59 | 2.53E-01 | -2.10 | 6.36E-04 |
| LOC100310756 | 17.47 | 2.05E-03 | 4.76E-02 | -0.36 | 4.71E-01 | -2.22 | 3.41E-04 |
| TMEM217 | 17.45 | 2.07E-03 | 4.76E-02 | -1.10 | 4.15E-02 | -1.69 | 3.92E-03 |
| ATP5G2 | 17.45 | 2.06E-03 | 4.76E-02 | -1.15 | 3.41E-02 | -1.64 | 4.76E-03 |
| CLVS1 | 17.48 | 2.04E-03 | 4.76E-02 | -0.91 | 8.40E-02 | -1.86 | 1.90E-03 |
| MC3R | 17.51 | 2.01E-03 | 4.76E-02 | -0.69 | 1.84E-01 | -2.04 | 8.58E-04 |
| SNORA74A | 17.45 | 2.06E-03 | 4.76E-02 | -0.36 | 4.70E-01 | -2.22 | 3.45E-04 |
| LOC284632 | 17.48 | 2.04E-03 | 4.76E-02 | 1.44 | 1.04E-02 | 1.35 | 1.55E-02 |
| DBF4B | 17.44 | 2.07E-03 | 4.77E-02 | 0.94 | 7.75E-02 | -1.84 | 2.11E-03 |
| SLC51B | 17.43 | 2.08E-03 | 4.78E-02 | 0.78 | 1.34E-01 | -1.96 | 1.22E-03 |
| CACTIN | 17.42 | 2.09E-03 | 4.78E-02 | 0.29 | 5.60E-01 | -2.25 | 2.94E-04 |
| RP11-1E4.1 | 17.41 | 2.10E-03 | 4.80E-02 | -1.11 | 3.97E-02 | -1.67 | 4.19E-03 |
| TMEM136 | 17.40 | 2.10E-03 | 4.80E-02 | 0.70 | 1.77E-01 | -2.02 | 9.41E-04 |
| RP11-38L15.2 | 17.40 | 2.10E-03 | 4.80E-02 | 1.35 | 1.52E-02 | 1.44 | 1.09E-02 |
| ORC3 | 17.38 | 2.12E-03 | 4.81E-02 | 1.25 | 2.29E-02 | 1.54 | 7.36E-03 |
| CNNM3 | 17.37 | 2.12E-03 | 4.81E-02 | -1.07 | 4.64E-02 | -1.71 | 3.64E-03 |
| NADK | 17.38 | 2.12E-03 | 4.81E-02 | -0.76 | 1.44E-01 | -1.97 | 1.17E-03 |
| PCDHB13 | 17.38 | 2.12E-03 | 4.81E-02 | -0.56 | 2.76E-01 | -2.11 | 6.11E-04 |
| COPE | 17.37 | 2.12E-03 | 4.81E-02 | 1.36 | 1.48E-02 | 1.43 | 1.14E-02 |
| ZDHHC13 | 17.38 | 2.12E-03 | 4.81E-02 | 0.87 | 9.91E-02 | -1.89 | 1.70E-03 |
| PIWIL4 | 17.38 | 2.12E-03 | 4.81E-02 | 1.61 | 5.15E-03 | 1.17 | 3.26E-02 |
| PQBP1 | 17.37 | 2.13E-03 | 4.82E-02 | -0.95 | 7.42E-02 | -1.82 | 2.28E-03 |
| TNNT1 | 17.32 | 2.17E-03 | 4.85E-02 | 1.48 | 8.93E-03 | 1.30 | 1.94E-02 |
| S100A8 | 17.32 | 2.17E-03 | 4.85E-02 | -1.82 | 2.10E-03 | -0.92 | 8.25E-02 |
| SLC7A9 | 17.33 | 2.16E-03 | 4.85E-02 | -1.12 | 3.84E-02 | -1.65 | 4.50E-03 |
| C14ORF80 | 17.32 | 2.17E-03 | 4.85E-02 | -0.72 | 1.63E-01 | -1.99 | 1.06E-03 |
| FLJ21408 | 17.34 | 2.15E-03 | 4.85E-02 | -0.75 | 1.48E-01 | -1.97 | 1.16E-03 |
| IPMK | 17.34 | 2.15E-03 | 4.85E-02 | 1.32 | 1.73E-02 | 1.46 | 9.91E-03 |
| ZNF30 | 17.32 | 2.17E-03 | 4.85E-02 | -0.99 | 6.38E-02 | -1.78 | 2.71E-03 |
| TRIM34 | 17.33 | 2.16E-03 | 4.85E-02 | -0.36 | 4.78E-01 | -2.21 | 3.62E-04 |
| ATP4B | 17.34 | 2.16E-03 | 4.85E-02 | 0.89 | 9.28E-02 | -1.87 | 1.85E-03 |
| ERBB2 | 17.33 | 2.16E-03 | 4.85E-02 | -0.56 | 2.73E-01 | -2.10 | 6.31E-04 |
| PPP1R16A | 17.30 | 2.18E-03 | 4.88E-02 | 1.56 | 6.46E-03 | 1.21 | 2.70E-02 |
| SSTR1 | 17.30 | 2.19E-03 | 4.88E-02 | 1.41 | 1.18E-02 | -1.36 | 1.49E-02 |
| HIST2H2AA3 | 17.30 | 2.19E-03 | 4.88E-02 | -1.32 | 1.70E-02 | -1.45 | 1.03E-02 |
| CLCC1 | 17.30 | 2.19E-03 | 4.88E-02 | -0.93 | 7.90E-02 | -1.82 | 2.22E-03 |
| FDPSP5 | 17.29 | 2.20E-03 | 4.89E-02 | -0.70 | 1.78E-01 | -2.01 | 9.90E-04 |
| HTR1D | 17.27 | 2.21E-03 | 4.91E-02 | 1.43 | 1.08E-02 | 1.34 | 1.64E-02 |
| GPATCH2L | 17.27 | 2.22E-03 | 4.92E-02 | -0.19 | 7.04E-01 | -2.27 | 2.52E-04 |
| HES2 | 17.27 | 2.22E-03 | 4.92E-02 | -0.17 | 7.28E-01 | -2.28 | 2.44E-04 |
| KCNK15 | 17.26 | 2.23E-03 | 4.93E-02 | 1.17 | 3.20E-02 | 1.60 | 5.59E-03 |
| GABARAPL1 | 17.25 | 2.23E-03 | 4.93E-02 | 1.29 | 1.97E-02 | 1.48 | 9.10E-03 |
| EGF | 17.25 | 2.24E-03 | 4.93E-02 | -1.40 | 1.22E-02 | -1.37 | 1.47E-02 |
| ZSCAN4 | 17.25 | 2.24E-03 | 4.93E-02 | 1.19 | 2.95E-02 | -1.58 | 6.09E-03 |
| TP73 | 17.24 | 2.24E-03 | 4.94E-02 | 0.12 | 8.14E-01 | -2.30 | 2.21E-04 |
| NRAS | 17.24 | 2.25E-03 | 4.94E-02 | 1.28 | 2.04E-02 | 1.49 | 8.83E-03 |
| PEX3 | 17.23 | 2.25E-03 | 4.95E-02 | -0.38 | 4.56E-01 | -2.19 | 3.97E-04 |
| IFT88 | 17.22 | 2.26E-03 | 4.95E-02 | -0.88 | 9.48E-02 | -1.86 | 1.92E-03 |
| PF4 | 17.21 | 2.27E-03 | 4.95E-02 | -1.03 | 5.38E-02 | -1.72 | 3.40E-03 |
| POLG2 | 17.21 | 2.27E-03 | 4.95E-02 | -1.10 | 4.16E-02 | -1.66 | 4.39E-03 |
| RPGRIP1 | 17.21 | 2.28E-03 | 4.95E-02 | -1.05 | 5.14E-02 | -1.71 | 3.57E-03 |
| COL18A1 | 17.21 | 2.28E-03 | 4.95E-02 | -0.95 | 7.33E-02 | -1.80 | 2.50E-03 |
| KIF2C | 17.21 | 2.28E-03 | 4.95E-02 | -0.85 | 1.05E-01 | -1.88 | 1.75E-03 |
| TNRC6A | 17.20 | 2.28E-03 | 4.95E-02 | -1.29 | 1.97E-02 | -1.48 | 9.32E-03 |
| ALMS1-IT1 | 17.22 | 2.26E-03 | 4.95E-02 | -1.28 | 2.00E-02 | -1.48 | 9.13E-03 |
| PHYHD1 | 17.22 | 2.26E-03 | 4.95E-02 | -0.88 | 9.56E-02 | -1.86 | 1.91E-03 |
| C19ORF80 | 17.21 | 2.28E-03 | 4.95E-02 | -0.46 | 3.62E-01 | -2.15 | 5.06E-04 |
| C4BPB | 17.19 | 2.29E-03 | 4.97E-02 | -1.08 | 4.46E-02 | -1.67 | 4.14E-03 |
| GAGE1 | 17.18 | 2.31E-03 | 4.99E-02 | 0.87 | 9.93E-02 | 1.86 | 1.87E-03 |
| ETS1 | 17.18 | 2.31E-03 | 4.99E-02 | 1.94 | 1.18E-03 | -0.73 | 1.57E-01 |
| LMOD2 | 17.16 | 2.32E-03 | 4.99E-02 | 1.46 | 9.71E-03 | 1.30 | 1.93E-02 |
| MNAT1 | 17.17 | 2.31E-03 | 4.99E-02 | 1.14 | 3.54E-02 | 1.62 | 5.27E-03 |
| MAN1B1 | 17.17 | 2.31E-03 | 4.99E-02 | -1.33 | 1.63E-02 | -1.43 | 1.14E-02 |
| STXBP2 | 17.16 | 2.33E-03 | 4.99E-02 | -0.55 | 2.78E-01 | -2.09 | 6.76E-04 |
| SPECC1 | 17.16 | 2.32E-03 | 4.99E-02 | -0.33 | 5.17E-01 | -2.21 | 3.64E-04 |
| LRPAP1 | 17.16 | 2.33E-03 | 4.99E-02 | 0.73 | 1.58E-01 | -1.97 | 1.19E-03 |
| PAXIP1OS | 17.17 | 2.32E-03 | 4.99E-02 | -0.17 | 7.37E-01 | -2.27 | 2.54E-04 |
| HMHA1 | 17.14 | 2.34E-03 | 5.00E-02 | -1.08 | 4.57E-02 | -1.67 | 4.15E-03 |
| AC018755.16 | 17.15 | 2.33E-03 | 5.00E-02 | -0.46 | 3.65E-01 | -2.14 | 5.17E-04 |
| PSMA3 | 17.15 | 2.34E-03 | 5.00E-02 | -0.85 | 1.05E-01 | -1.87 | 1.79E-03 |
| ING2 | 17.14 | 2.34E-03 | 5.00E-02 | -1.30 | 1.86E-02 | -1.46 | 1.02E-02 |
| LTBP3 | 17.14 | 2.34E-03 | 5.00E-02 | -0.80 | 1.26E-01 | -1.91 | 1.50E-03 |

**Table S2. The 287 DEGs identified in 6 datasets from PCOS6.**

| **PCOS6** | **Fisher** | | | **gse1615** | | **gse10946_obese** | | **gse43264** | | **gse48301_eEP** | | **gse48301_eSF** | | **gse48301_eMSC** | |
| --- | --- | --- | --- | --- | --- | --- | --- | --- | --- | --- | --- | --- | --- | --- | --- |
| gene | meta.  stastic | meta.  p_value | meta.  q_value | ind.  statistic | Ind  .p_value | Ind  .statistic | Ind  .p_value | Ind  .statistic | Ind  .p_value | ind.  statistic | Ind  .p_value | ind.  statistic | ind.  p_value | Ind  .statistic | ind.  p_value |
| TMEM45A | 52.97 | 4.45E-06 | 1.33E-02 | -0.23 | 6.46E-01 | -0.78 | 1.45E-01 | 0.91 | 8.81E-02 | 3.72 | 1.15E-04 | 1.90 | 4.71E-03 | 2.48 | 7.02E-04 |
| LOX | 52.39 | 4.45E-06 | 1.33E-02 | 1.23 | 3.49E-02 | -1.18 | 3.63E-02 | -1.44 | 1.12E-02 | 2.26 | 1.82E-03 | 1.76 | 7.29E-03 | 1.39 | 2.23E-02 |
| RHOJ | 54.86 | 2.97E-06 | 1.33E-02 | 2.14 | 2.12E-03 | 0.32 | 5.29E-01 | -1.24 | 2.53E-02 | 3.26 | 2.26E-04 | -2.47 | 8.57E-04 | -0.67 | 2.21E-01 |
| MBNL3 | 51.49 | 4.75E-06 | 1.33E-02 | -2.11 | 2.34E-03 | 1.10 | 4.81E-02 | -1.62 | 5.12E-03 | -0.56 | 3.07E-01 | 0.92 | 1.03E-01 | 2.70 | 3.61E-04 |
| ICAM1 | 49.72 | 8.01E-06 | 1.50E-02 | 0.77 | 1.51E-01 | -0.04 | 9.32E-01 | -1.66 | 4.50E-03 | -0.17 | 7.56E-01 | -3.67 | 4.39E-05 | -2.46 | 7.59E-04 |
| SEMA3A | 50.14 | 7.42E-06 | 1.50E-02 | -1.14 | 4.73E-02 | 1.04 | 5.93E-02 | -2.49 | 1.29E-04 | -1.11 | 5.97E-02 | -2.12 | 2.48E-03 | -0.64 | 2.42E-01 |
| TNFSF10 | 49.34 | 9.79E-06 | 1.54E-02 | -2.61 | 5.91E-04 | -0.72 | 1.76E-01 | -0.90 | 9.29E-02 | -1.12 | 5.75E-02 | -1.83 | 5.84E-03 | -1.79 | 5.95E-03 |
| SVEP1 | 48.97 | 1.10E-05 | 1.54E-02 | -1.52 | 1.38E-02 | 0.02 | 9.61E-01 | -1.66 | 4.47E-03 | 3.11 | 2.82E-04 | 0.56 | 3.02E-01 | 1.87 | 4.58E-03 |
| NFKBIZ | 45.65 | 2.85E-05 | 1.56E-02 | -1.37 | 2.28E-02 | -0.54 | 3.02E-01 | -1.38 | 1.41E-02 | -0.56 | 3.08E-01 | -2.70 | 4.41E-04 | -1.66 | 9.32E-03 |
| CDH11 | 47.32 | 1.87E-05 | 1.56E-02 | -1.58 | 1.16E-02 | 0.39 | 4.51E-01 | -1.19 | 3.12E-02 | 4.91 | 2.17E-05 | 0.31 | 5.60E-01 | 1.33 | 2.67E-02 |
| REL | 48.22 | 1.34E-05 | 1.56E-02 | -1.04 | 6.53E-02 | 0.62 | 2.37E-01 | -1.04 | 5.59E-02 | -1.09 | 6.35E-02 | -3.75 | 3.15E-05 | -1.43 | 1.95E-02 |
| TLE4 | 45.44 | 3.00E-05 | 1.56E-02 | 3.08 | 1.93E-04 | -0.13 | 7.90E-01 | -0.07 | 8.84E-01 | -0.15 | 7.75E-01 | -2.08 | 2.71E-03 | -2.61 | 4.78E-04 |
| PUS7 | 45.26 | 3.06E-05 | 1.56E-02 | -1.00 | 7.33E-02 | 0.23 | 6.49E-01 | -2.61 | 7.80E-05 | -1.40 | 2.34E-02 | 1.28 | 3.30E-02 | 1.13 | 5.17E-02 |
| MRPL20 | 45.74 | 2.73E-05 | 1.56E-02 | -0.68 | 1.97E-01 | -0.96 | 7.70E-02 | 0.81 | 1.25E-01 | 1.83 | 6.13E-03 | 2.50 | 7.80E-04 | 1.55 | 1.29E-02 |
| FLRT2 | 47.59 | 1.72E-05 | 1.56E-02 | -2.72 | 4.41E-04 | 0.81 | 1.31E-01 | -1.12 | 4.09E-02 | 4.14 | 6.47E-05 | 0.55 | 3.07E-01 | -0.01 | 9.90E-01 |
| RGS10 | 47.29 | 1.87E-05 | 1.56E-02 | 1.66 | 9.06E-03 | 0.26 | 6.10E-01 | 0.40 | 4.29E-01 | -1.07 | 6.74E-02 | 3.40 | 8.52E-05 | 1.92 | 3.95E-03 |
| MAX | 45.83 | 2.70E-05 | 1.56E-02 | 2.64 | 5.42E-04 | 1.03 | 6.07E-02 | 0.13 | 7.84E-01 | 1.10 | 6.06E-02 | -2.01 | 3.36E-03 | -1.40 | 2.13E-02 |
| LYRM4 | 45.43 | 3.00E-05 | 1.56E-02 | 1.50 | 1.48E-02 | 0.62 | 2.39E-01 | 1.01 | 6.17E-02 | -1.86 | 5.52E-03 | 1.91 | 4.56E-03 | 1.35 | 2.48E-02 |
| MAD2L1 | 47.84 | 1.60E-05 | 1.56E-02 | -4.21 | 2.20E-05 | 0.62 | 2.37E-01 | 0.54 | 2.88E-01 | -0.11 | 8.43E-01 | 2.12 | 2.47E-03 | 1.55 | 1.31E-02 |
| LRRN1 | 45.84 | 2.70E-05 | 1.56E-02 | -2.10 | 2.39E-03 | 0.84 | 1.17E-01 | 1.77 | 2.73E-03 | -1.87 | 5.30E-03 | -1.19 | 4.39E-02 | -0.26 | 6.24E-01 |
| PREX2 | 46.08 | 2.61E-05 | 1.56E-02 | -0.84 | 1.20E-01 | 1.13 | 4.29E-02 | -2.10 | 6.61E-04 | -1.38 | 2.45E-02 | -2.18 | 2.02E-03 | 0.29 | 5.87E-01 |
| SGOL2 | 45.42 | 3.00E-05 | 1.56E-02 | -2.64 | 5.33E-04 | 0.26 | 6.14E-01 | -0.89 | 9.57E-02 | -0.58 | 2.92E-01 | 2.29 | 1.41E-03 | 1.61 | 1.06E-02 |
| NR1D1 | 45.05 | 3.41E-05 | 1.67E-02 | -0.24 | 6.35E-01 | -0.02 | 9.67E-01 | 0.53 | 2.96E-01 | 0.43 | 4.26E-01 | -4.23 | 6.82E-06 | -2.74 | 3.12E-04 |
| XAF1 | 44.69 | 3.83E-05 | 1.79E-02 | -0.72 | 1.74E-01 | -1.03 | 6.15E-02 | -1.61 | 5.49E-03 | -1.44 | 2.01E-02 | -1.29 | 3.16E-02 | -1.83 | 5.29E-03 |
| NXF1 | 44.08 | 4.90E-05 | 2.04E-02 | 0.54 | 2.98E-01 | 0.19 | 7.14E-01 | -1.81 | 2.34E-03 | -0.76 | 1.78E-01 | -2.57 | 6.34E-04 | -1.86 | 4.75E-03 |
| SERPINI1 | 44.24 | 4.72E-05 | 2.04E-02 | 1.22 | 3.67E-02 | -0.36 | 4.78E-01 | -1.14 | 3.70E-02 | -1.73 | 8.19E-03 | 0.91 | 1.05E-01 | 2.64 | 4.42E-04 |
| ELOVL7 | 44.15 | 4.84E-05 | 2.04E-02 | 0.16 | 7.47E-01 | 0.47 | 3.64E-01 | -2.99 | 1.72E-05 | -2.56 | 8.57E-04 | -0.27 | 6.11E-01 | -0.91 | 1.06E-01 |
| WDR41 | 43.56 | 5.73E-05 | 2.08E-02 | -3.45 | 8.46E-05 | 1.14 | 4.17E-02 | 1.32 | 1.84E-02 | 0.19 | 7.19E-01 | 0.30 | 5.68E-01 | 1.55 | 1.31E-02 |
| FN1 | 43.36 | 5.93E-05 | 2.08E-02 | 2.67 | 5.01E-04 | -0.30 | 5.56E-01 | -0.44 | 3.82E-01 | 4.06 | 7.89E-05 | 0.75 | 1.74E-01 | 0.61 | 2.62E-01 |
| STX8 | 43.89 | 5.34E-05 | 2.08E-02 | -1.48 | 1.58E-02 | 0.31 | 5.41E-01 | 1.15 | 3.59E-02 | -0.97 | 9.35E-02 | 2.79 | 3.42E-04 | 1.30 | 3.00E-02 |
| LDLR | 43.43 | 5.90E-05 | 2.08E-02 | -0.77 | 1.50E-01 | 0.27 | 5.92E-01 | -2.27 | 3.22E-04 | 1.87 | 5.29E-03 | -1.15 | 5.05E-02 | -1.15 | 4.87E-02 |
| FBXO4 | 43.65 | 5.67E-05 | 2.08E-02 | -1.36 | 2.30E-02 | 0.41 | 4.21E-01 | 0.85 | 1.10E-01 | -0.43 | 4.32E-01 | 1.92 | 4.37E-03 | 2.98 | 1.66E-04 |
| SMAD2 | 43.01 | 6.79E-05 | 2.19E-02 | -1.13 | 4.83E-02 | -0.13 | 7.96E-01 | -0.11 | 8.27E-01 | -0.56 | 3.13E-01 | 1.70 | 8.60E-03 | 4.68 | 5.34E-06 |
| IGF1 | 43.15 | 6.47E-05 | 2.19E-02 | 0.68 | 1.96E-01 | 0.41 | 4.27E-01 | -0.63 | 2.22E-01 | 6.33 | 2.37E-06 | -1.47 | 1.78E-02 | 0.33 | 5.42E-01 |
| TSPAN8 | 42.99 | 6.82E-05 | 2.19E-02 | -3.84 | 3.77E-05 | -0.44 | 3.93E-01 | 1.13 | 3.86E-02 | -1.06 | 7.01E-02 | 0.53 | 3.24E-01 | -1.24 | 3.57E-02 |
| DDX58 | 42.86 | 7.24E-05 | 2.26E-02 | -0.71 | 1.82E-01 | -0.31 | 5.44E-01 | -2.70 | 5.37E-05 | -2.99 | 3.42E-04 | 0.08 | 8.81E-01 | 0.55 | 3.07E-01 |
| ING5 | 42.74 | 7.51E-05 | 2.28E-02 | 3.50 | 7.63E-05 | -0.45 | 3.87E-01 | -1.42 | 1.20E-02 | -0.11 | 8.44E-01 | -1.28 | 3.33E-02 | 1.13 | 5.24E-02 |
| HSPB11 | 42.27 | 9.02E-05 | 2.60E-02 | -1.67 | 8.67E-03 | -0.40 | 4.34E-01 | 0.82 | 1.19E-01 | 0.20 | 7.08E-01 | 2.15 | 2.24E-03 | 2.38 | 9.34E-04 |
| LCN2 | 42.32 | 8.90E-05 | 2.60E-02 | -1.94 | 3.82E-03 | 1.05 | 5.79E-02 | -0.38 | 4.46E-01 | 0.80 | 1.56E-01 | -1.03 | 7.41E-02 | -2.56 | 5.65E-04 |
| FBN1 | 42.16 | 9.41E-05 | 2.62E-02 | -1.09 | 5.48E-02 | 0.51 | 3.29E-01 | -0.24 | 6.31E-01 | 5.24 | 1.69E-05 | -0.30 | 5.74E-01 | 1.77 | 6.34E-03 |
| DACH1 | 42.13 | 9.61E-05 | 2.62E-02 | -2.09 | 2.44E-03 | 0.73 | 1.69E-01 | -2.18 | 4.74E-04 | -1.22 | 4.20E-02 | -0.39 | 4.64E-01 | 0.73 | 1.87E-01 |
| GEMIN6 | 42.10 | 9.79E-05 | 2.62E-02 | -1.21 | 3.74E-02 | 0.01 | 9.81E-01 | 1.16 | 3.49E-02 | 0.68 | 2.25E-01 | 1.78 | 6.80E-03 | 2.70 | 3.69E-04 |
| PCDH18 | 41.98 | 1.02E-04 | 2.66E-02 | 1.65 | 9.44E-03 | -0.17 | 7.32E-01 | -0.15 | 7.58E-01 | 6.13 | 7.12E-06 | 0.13 | 8.06E-01 | 1.35 | 2.55E-02 |
| ATP5O | 41.40 | 1.28E-04 | 2.68E-02 | -1.17 | 4.20E-02 | 0.11 | 8.32E-01 | 0.59 | 2.48E-01 | -1.15 | 5.27E-02 | 2.58 | 6.23E-04 | 1.95 | 3.59E-03 |
| TGFBI | 41.45 | 1.26E-04 | 2.68E-02 | -0.21 | 6.82E-01 | 0.60 | 2.50E-01 | 0.78 | 1.40E-01 | 3.35 | 2.01E-04 | 1.15 | 5.05E-02 | 1.91 | 4.12E-03 |
| CD83 | 41.69 | 1.14E-04 | 2.68E-02 | -0.22 | 6.59E-01 | 0.28 | 5.86E-01 | 1.27 | 2.22E-02 | -1.27 | 3.54E-02 | -2.52 | 7.37E-04 | -1.92 | 3.94E-03 |
| TOMM22 | 41.66 | 1.15E-04 | 2.68E-02 | -2.69 | 4.67E-04 | 0.94 | 8.49E-02 | 1.22 | 2.71E-02 | 1.28 | 3.45E-02 | -0.66 | 2.27E-01 | 0.91 | 1.07E-01 |
| PLEKHM3 | 41.68 | 1.15E-04 | 2.68E-02 | -0.86 | 1.13E-01 | -0.67 | 2.04E-01 | -0.72 | 1.65E-01 | -1.62 | 1.14E-02 | -1.13 | 5.39E-02 | 2.68 | 3.80E-04 |
| ARID4B | 41.88 | 1.05E-04 | 2.68E-02 | -2.12 | 2.23E-03 | 0.93 | 8.61E-02 | -1.02 | 5.98E-02 | -1.17 | 4.82E-02 | -1.45 | 1.93E-02 | -1.02 | 7.55E-02 |
| TSEN15 | 41.65 | 1.15E-04 | 2.68E-02 | 3.49 | 7.83E-05 | -0.10 | 8.40E-01 | -1.13 | 3.85E-02 | -0.22 | 6.88E-01 | 1.75 | 7.42E-03 | 1.04 | 6.98E-02 |
| PNPLA4 | 41.47 | 1.25E-04 | 2.68E-02 | -1.10 | 5.28E-02 | -0.26 | 6.14E-01 | -1.63 | 5.11E-03 | -0.54 | 3.24E-01 | 2.84 | 3.06E-04 | 1.09 | 6.04E-02 |
| INPP4B | 41.57 | 1.19E-04 | 2.68E-02 | 1.01 | 7.17E-02 | -0.11 | 8.23E-01 | -0.84 | 1.11E-01 | 2.44 | 1.14E-03 | -1.57 | 1.34E-02 | 1.65 | 9.47E-03 |
| FAM26E | 41.43 | 1.26E-04 | 2.68E-02 | 0.89 | 1.04E-01 | -0.11 | 8.34E-01 | -0.83 | 1.17E-01 | -0.83 | 1.44E-01 | 2.39 | 1.12E-03 | 2.53 | 6.19E-04 |
| STK11IP | 41.38 | 1.29E-04 | 2.68E-02 | 4.80 | 5.04E-06 | 0.10 | 8.42E-01 | -1.47 | 9.96E-03 | 0.83 | 1.43E-01 | -0.36 | 5.03E-01 | 0.51 | 3.41E-01 |
| MRPS23 | 41.24 | 1.37E-04 | 2.75E-02 | -0.93 | 9.17E-02 | 0.90 | 9.48E-02 | 0.63 | 2.21E-01 | -0.33 | 5.45E-01 | 2.22 | 1.76E-03 | 2.54 | 6.01E-04 |
| NLGN1 | 41.27 | 1.35E-04 | 2.75E-02 | -1.35 | 2.40E-02 | 0.67 | 2.02E-01 | -0.97 | 7.17E-02 | 2.42 | 1.20E-03 | 0.75 | 1.75E-01 | 1.50 | 1.51E-02 |
| CIRBP | 41.00 | 1.48E-04 | 2.81E-02 | -0.49 | 3.43E-01 | -0.50 | 3.34E-01 | -2.03 | 9.19E-04 | 1.73 | 8.23E-03 | -1.52 | 1.56E-02 | -0.96 | 9.24E-02 |
| POLE4 | 41.01 | 1.48E-04 | 2.81E-02 | 0.58 | 2.64E-01 | 1.10 | 4.73E-02 | 1.23 | 2.65E-02 | -1.41 | 2.24E-02 | 1.79 | 6.57E-03 | 1.35 | 2.56E-02 |
| COL5A2 | 41.01 | 1.48E-04 | 2.81E-02 | -0.70 | 1.84E-01 | -0.46 | 3.77E-01 | -0.52 | 3.04E-01 | 2.37 | 1.38E-03 | 1.91 | 4.63E-03 | 1.66 | 9.23E-03 |
| GCH1 | 40.87 | 1.54E-04 | 2.87E-02 | -0.74 | 1.65E-01 | -0.12 | 8.10E-01 | -2.40 | 1.86E-04 | -1.72 | 8.41E-03 | -1.78 | 6.87E-03 | -0.05 | 9.29E-01 |
| PTPLAD2 | 40.83 | 1.56E-04 | 2.87E-02 | -1.10 | 5.38E-02 | -0.60 | 2.48E-01 | 0.59 | 2.53E-01 | 0.54 | 3.30E-01 | 1.99 | 3.55E-03 | 2.71 | 3.45E-04 |
| IGFBP5 | 40.62 | 1.67E-04 | 2.94E-02 | 1.83 | 5.29E-03 | 0.38 | 4.63E-01 | -0.74 | 1.56E-01 | 3.56 | 1.45E-04 | -0.79 | 1.55E-01 | 0.75 | 1.77E-01 |
| MAOB | 40.68 | 1.64E-04 | 2.94E-02 | 1.82 | 5.45E-03 | 0.13 | 7.92E-01 | -0.32 | 5.24E-01 | 2.86 | 4.54E-04 | 0.47 | 3.84E-01 | 1.94 | 3.73E-03 |
| N4BP2L1 | 40.66 | 1.65E-04 | 2.94E-02 | -2.61 | 5.99E-04 | -0.84 | 1.19E-01 | 0.97 | 7.11E-02 | -1.87 | 5.28E-03 | -0.54 | 3.15E-01 | 0.75 | 1.76E-01 |
| BTG2 | 40.58 | 1.70E-04 | 2.94E-02 | 1.59 | 1.13E-02 | 0.09 | 8.59E-01 | -0.52 | 3.10E-01 | 1.67 | 9.75E-03 | -2.08 | 2.73E-03 | -1.43 | 1.93E-02 |
| WDR60 | 40.51 | 1.74E-04 | 2.97E-02 | -2.09 | 2.47E-03 | -0.32 | 5.28E-01 | -1.89 | 1.67E-03 | 1.77 | 7.22E-03 | -0.79 | 1.56E-01 | 0.24 | 6.54E-01 |
| SLC3A2 | 40.32 | 1.86E-04 | 2.98E-02 | 2.42 | 9.48E-04 | 0.33 | 5.21E-01 | 0.72 | 1.68E-01 | 1.62 | 1.13E-02 | -1.51 | 1.63E-02 | -0.89 | 1.14E-01 |
| EDNRA | 40.44 | 1.79E-04 | 2.98E-02 | 0.46 | 3.73E-01 | 0.13 | 8.04E-01 | -0.97 | 7.01E-02 | 4.63 | 3.32E-05 | 1.81 | 6.12E-03 | 0.47 | 3.86E-01 |
| RAB32 | 40.29 | 1.87E-04 | 2.98E-02 | 1.48 | 1.58E-02 | -0.31 | 5.40E-01 | 0.63 | 2.19E-01 | -1.62 | 1.14E-02 | 2.27 | 1.52E-03 | 1.12 | 5.49E-02 |
| TNFAIP6 | 40.25 | 1.88E-04 | 2.98E-02 | -0.48 | 3.52E-01 | 0.87 | 1.06E-01 | -0.54 | 2.93E-01 | -1.13 | 5.59E-02 | 3.13 | 1.58E-04 | 1.44 | 1.87E-02 |
| UQCRB | 40.36 | 1.84E-04 | 2.98E-02 | -1.99 | 3.27E-03 | 0.51 | 3.24E-01 | 1.66 | 4.32E-03 | 0.56 | 3.14E-01 | 0.34 | 5.27E-01 | 2.09 | 2.28E-03 |
| CEP55 | 40.21 | 1.91E-04 | 2.98E-02 | -0.67 | 2.06E-01 | 1.15 | 3.94E-02 | 1.09 | 4.62E-02 | -0.36 | 5.12E-01 | 2.41 | 1.05E-03 | 1.66 | 9.18E-03 |
| GUCY1B3 | 40.01 | 2.01E-04 | 3.09E-02 | 0.47 | 3.60E-01 | 0.61 | 2.44E-01 | -1.95 | 1.25E-03 | -0.48 | 3.82E-01 | 1.92 | 4.40E-03 | 1.60 | 1.11E-02 |
| ZBTB20 | 39.74 | 2.20E-04 | 3.22E-02 | -1.73 | 7.14E-03 | -1.45 | 1.32E-02 | -0.54 | 2.93E-01 | -0.23 | 6.75E-01 | -1.89 | 4.79E-03 | -1.34 | 2.62E-02 |
| PAMR1 | 39.84 | 2.14E-04 | 3.22E-02 | 0.60 | 2.50E-01 | -0.74 | 1.64E-01 | 0.36 | 4.70E-01 | 4.34 | 4.75E-05 | -1.31 | 2.97E-02 | 0.99 | 8.23E-02 |
| ZFP1 | 39.70 | 2.24E-04 | 3.22E-02 | -2.86 | 3.09E-04 | 0.45 | 3.85E-01 | -1.36 | 1.54E-02 | -1.68 | 9.44E-03 | 0.61 | 2.61E-01 | 0.34 | 5.28E-01 |
| ULK4 | 39.66 | 2.26E-04 | 3.22E-02 | -1.03 | 6.73E-02 | -0.49 | 3.43E-01 | 1.30 | 1.97E-02 | 1.45 | 1.93E-02 | 1.71 | 8.43E-03 | 1.27 | 3.30E-02 |
| SFPQ | 39.63 | 2.26E-04 | 3.22E-02 | -1.93 | 3.97E-03 | 0.93 | 8.79E-02 | -1.55 | 6.92E-03 | 0.58 | 2.91E-01 | -0.31 | 5.61E-01 | -1.77 | 6.31E-03 |
| DPY19L2 | 39.66 | 2.25E-04 | 3.22E-02 | -3.23 | 1.45E-04 | -0.75 | 1.60E-01 | -1.71 | 3.49E-03 | 0.10 | 8.55E-01 | -0.14 | 7.95E-01 | 1.18 | 4.45E-02 |
| MYLK | 39.37 | 2.44E-04 | 3.24E-02 | 1.48 | 1.61E-02 | -1.19 | 3.49E-02 | -0.54 | 2.93E-01 | 1.66 | 1.01E-02 | 1.40 | 2.26E-02 | 1.02 | 7.46E-02 |
| TNFAIP3 | 39.33 | 2.45E-04 | 3.24E-02 | -0.87 | 1.10E-01 | 0.54 | 2.99E-01 | -0.55 | 2.80E-01 | 1.63 | 1.10E-02 | -2.32 | 1.35E-03 | -1.41 | 2.09E-02 |
| EIF4E2 | 39.42 | 2.39E-04 | 3.24E-02 | -1.19 | 3.97E-02 | 0.43 | 4.09E-01 | 1.10 | 4.42E-02 | 0.23 | 6.67E-01 | 2.12 | 2.49E-03 | 2.08 | 2.32E-03 |
| CD248 | 39.35 | 2.45E-04 | 3.24E-02 | 1.63 | 9.81E-03 | -0.08 | 8.67E-01 | -0.38 | 4.52E-01 | 5.08 | 1.93E-05 | 0.54 | 3.14E-01 | 0.87 | 1.22E-01 |
| COL6A1 | 39.48 | 2.36E-04 | 3.24E-02 | 1.88 | 4.57E-03 | 1.40 | 1.57E-02 | -2.40 | 1.90E-04 | 0.28 | 6.07E-01 | -0.29 | 5.86E-01 | -0.32 | 5.52E-01 |
| TMPO | 39.53 | 2.33E-04 | 3.24E-02 | -1.43 | 1.85E-02 | 0.29 | 5.70E-01 | -1.64 | 4.77E-03 | 1.21 | 4.35E-02 | 1.31 | 2.98E-02 | 1.21 | 3.99E-02 |
| RPP30 | 39.22 | 2.52E-04 | 3.29E-02 | -0.69 | 1.93E-01 | 0.55 | 2.94E-01 | 1.21 | 2.84E-02 | -0.48 | 3.83E-01 | 1.57 | 1.31E-02 | 2.68 | 3.77E-04 |
| ACTN1 | 38.42 | 3.22E-04 | 3.33E-02 | 2.32 | 1.25E-03 | -0.61 | 2.45E-01 | -0.61 | 2.39E-01 | 2.29 | 1.70E-03 | -0.15 | 7.81E-01 | 1.16 | 4.69E-02 |
| IGBP1 | 38.41 | 3.23E-04 | 3.33E-02 | -0.83 | 1.25E-01 | -0.19 | 7.14E-01 | 0.89 | 9.34E-02 | -1.37 | 2.55E-02 | 1.93 | 4.26E-03 | 1.84 | 5.02E-03 |
| LSM1 | 38.59 | 3.03E-04 | 3.33E-02 | -2.73 | 4.23E-04 | 0.68 | 1.96E-01 | 0.81 | 1.25E-01 | 0.24 | 6.59E-01 | 1.70 | 8.78E-03 | 1.04 | 6.98E-02 |
| NFKB1 | 38.45 | 3.19E-04 | 3.33E-02 | 0.39 | 4.43E-01 | 0.62 | 2.38E-01 | -1.51 | 8.36E-03 | -0.96 | 9.39E-02 | -1.86 | 5.40E-03 | -1.63 | 1.00E-02 |
| GGH | 38.88 | 2.76E-04 | 3.33E-02 | -1.07 | 5.90E-02 | 0.12 | 8.05E-01 | 2.16 | 5.20E-04 | -1.02 | 7.81E-02 | 2.19 | 1.94E-03 | -0.03 | 9.62E-01 |
| TCF7L2 | 38.90 | 2.74E-04 | 3.33E-02 | -1.34 | 2.50E-02 | -1.63 | 6.75E-03 | -0.48 | 3.44E-01 | -0.35 | 5.16E-01 | -1.49 | 1.69E-02 | -1.74 | 7.08E-03 |
| SDHB | 38.65 | 2.95E-04 | 3.33E-02 | -0.37 | 4.71E-01 | 0.72 | 1.76E-01 | 0.95 | 7.68E-02 | -0.48 | 3.83E-01 | 2.39 | 1.09E-03 | 2.22 | 1.53E-03 |
| KLHDC10 | 38.63 | 2.96E-04 | 3.33E-02 | -1.01 | 7.09E-02 | 0.15 | 7.69E-01 | -0.97 | 7.08E-02 | -1.57 | 1.34E-02 | 2.05 | 2.91E-03 | 1.33 | 2.71E-02 |
| PDGFRA | 38.89 | 2.75E-04 | 3.33E-02 | -1.55 | 1.29E-02 | -0.47 | 3.66E-01 | -0.38 | 4.45E-01 | 4.37 | 4.51E-05 | -0.82 | 1.40E-01 | 0.60 | 2.71E-01 |
| TMEM220 | 38.50 | 3.13E-04 | 3.33E-02 | 1.26 | 3.19E-02 | 0.00 | 9.98E-01 | 1.79 | 2.51E-03 | -1.77 | 7.35E-03 | 1.67 | 9.42E-03 | 0.14 | 7.89E-01 |
| RAD51C | 38.42 | 3.23E-04 | 3.33E-02 | -1.39 | 2.12E-02 | 1.56 | 8.92E-03 | 0.64 | 2.17E-01 | -0.90 | 1.16E-01 | 0.89 | 1.13E-01 | 1.68 | 8.46E-03 |
| BGN | 38.85 | 2.77E-04 | 3.33E-02 | 3.41 | 9.20E-05 | -0.12 | 8.06E-01 | -1.87 | 1.79E-03 | 0.76 | 1.75E-01 | -0.78 | 1.61E-01 | 0.01 | 9.82E-01 |
| CENPV | 38.93 | 2.73E-04 | 3.33E-02 | 1.94 | 3.81E-03 | 0.69 | 1.88E-01 | 0.90 | 9.25E-02 | -1.94 | 4.34E-03 | 1.54 | 1.46E-02 | 0.11 | 8.38E-01 |
| TRPC6 | 38.74 | 2.88E-04 | 3.33E-02 | 1.10 | 5.35E-02 | 1.04 | 5.88E-02 | -1.60 | 5.66E-03 | 0.89 | 1.19E-01 | 0.25 | 6.34E-01 | 2.02 | 2.89E-03 |
| CASP2 | 38.42 | 3.23E-04 | 3.33E-02 | 2.27 | 1.47E-03 | 0.75 | 1.59E-01 | -1.02 | 5.95E-02 | 1.01 | 8.21E-02 | -1.29 | 3.23E-02 | 0.87 | 1.24E-01 |
| GABBR1 | 38.63 | 2.98E-04 | 3.33E-02 | -0.34 | 5.04E-01 | -0.78 | 1.43E-01 | -2.32 | 2.64E-04 | 0.50 | 3.65E-01 | -1.89 | 4.79E-03 | -0.87 | 1.23E-01 |
| MTMR1 | 38.53 | 3.09E-04 | 3.33E-02 | 2.36 | 1.12E-03 | 0.37 | 4.71E-01 | -0.50 | 3.22E-01 | -1.47 | 1.82E-02 | -1.47 | 1.80E-02 | -1.01 | 7.68E-02 |
| TM6SF1 | 38.83 | 2.78E-04 | 3.33E-02 | 1.93 | 3.87E-03 | -0.58 | 2.67E-01 | -1.11 | 4.22E-02 | -0.90 | 1.14E-01 | 0.22 | 6.72E-01 | 2.33 | 1.11E-03 |
| NRXN3 | 38.67 | 2.94E-04 | 3.33E-02 | -0.65 | 2.17E-01 | -1.36 | 1.85E-02 | -1.81 | 2.28E-03 | 0.68 | 2.24E-01 | -2.17 | 2.12E-03 | 0.05 | 9.26E-01 |
| SLC1A1 | 38.63 | 2.98E-04 | 3.33E-02 | 0.92 | 9.48E-02 | -0.95 | 8.12E-02 | -1.91 | 1.49E-03 | 0.22 | 6.82E-01 | 0.55 | 3.07E-01 | 2.18 | 1.70E-03 |
| PBK | 38.51 | 3.12E-04 | 3.33E-02 | -1.46 | 1.71E-02 | 0.93 | 8.72E-02 | -0.19 | 6.99E-01 | -0.36 | 5.01E-01 | 2.27 | 1.55E-03 | 1.82 | 5.37E-03 |
| GNRH1 | 39.04 | 2.64E-04 | 3.33E-02 | -0.78 | 1.46E-01 | -1.31 | 2.19E-02 | -1.64 | 4.84E-03 | -2.22 | 2.02E-03 | 0.79 | 1.55E-01 | 0.21 | 6.87E-01 |
| DEPDC1B | 38.57 | 3.06E-04 | 3.33E-02 | -0.80 | 1.37E-01 | 0.19 | 7.12E-01 | 1.41 | 1.26E-02 | -0.79 | 1.62E-01 | 2.18 | 2.03E-03 | 1.62 | 1.04E-02 |
| IMPAD1 | 38.32 | 3.29E-04 | 3.36E-02 | -1.81 | 5.76E-03 | -0.31 | 5.49E-01 | -0.95 | 7.57E-02 | 1.68 | 9.42E-03 | -0.66 | 2.27E-01 | 1.66 | 9.33E-03 |
| TMEM203 | 37.93 | 3.71E-04 | 3.47E-02 | 1.17 | 4.23E-02 | 0.17 | 7.35E-01 | 0.30 | 5.41E-01 | -1.86 | 5.43E-03 | 1.26 | 3.47E-02 | 2.16 | 1.83E-03 |
| ACTA2 | 38.22 | 3.44E-04 | 3.47E-02 | 0.33 | 5.15E-01 | 0.07 | 8.90E-01 | -0.95 | 7.69E-02 | 1.66 | 1.02E-02 | 1.64 | 1.05E-02 | 2.27 | 1.34E-03 |
| MT1F | 38.11 | 3.55E-04 | 3.47E-02 | -0.29 | 5.62E-01 | 0.71 | 1.79E-01 | 1.89 | 1.67E-03 | 2.14 | 2.42E-03 | 0.34 | 5.23E-01 | 1.35 | 2.50E-02 |
| TSPYL2 | 38.00 | 3.65E-04 | 3.47E-02 | -0.94 | 8.74E-02 | -0.30 | 5.57E-01 | -0.16 | 7.46E-01 | 0.17 | 7.53E-01 | -3.85 | 1.87E-05 | -1.60 | 1.10E-02 |
| RPL34 | 38.05 | 3.60E-04 | 3.47E-02 | -1.26 | 3.21E-02 | 0.07 | 8.82E-01 | 1.70 | 3.72E-03 | -1.17 | 4.94E-02 | 1.40 | 2.27E-02 | 1.17 | 4.63E-02 |
| EFEMP1 | 38.20 | 3.46E-04 | 3.47E-02 | -0.69 | 1.90E-01 | -0.14 | 7.88E-01 | -0.48 | 3.46E-01 | 2.38 | 1.34E-03 | 2.65 | 5.08E-04 | 0.82 | 1.44E-01 |
| FAM46C | 37.93 | 3.71E-04 | 3.47E-02 | 2.13 | 2.16E-03 | 0.78 | 1.44E-01 | -0.30 | 5.40E-01 | -2.08 | 2.83E-03 | -1.18 | 4.55E-02 | 0.60 | 2.68E-01 |
| SLC35D2 | 38.03 | 3.62E-04 | 3.47E-02 | -0.16 | 7.56E-01 | 0.64 | 2.25E-01 | 0.65 | 2.07E-01 | -0.63 | 2.55E-01 | 2.27 | 1.54E-03 | 2.67 | 3.99E-04 |
| DLGAP5 | 38.05 | 3.60E-04 | 3.47E-02 | -0.80 | 1.35E-01 | 0.95 | 8.25E-02 | 1.29 | 2.04E-02 | 0.44 | 4.20E-01 | 2.24 | 1.67E-03 | 1.26 | 3.44E-02 |
| TP53TG1 | 38.10 | 3.56E-04 | 3.47E-02 | -0.83 | 1.24E-01 | 0.60 | 2.49E-01 | 1.25 | 2.38E-02 | -0.59 | 2.85E-01 | 2.53 | 7.29E-04 | 1.25 | 3.48E-02 |
| DIO2 | 37.89 | 3.75E-04 | 3.48E-02 | -0.82 | 1.30E-01 | -0.71 | 1.79E-01 | 0.83 | 1.18E-01 | 3.61 | 1.38E-04 | 0.52 | 3.36E-01 | 1.17 | 4.67E-02 |
| SEC61A1 | 37.78 | 3.85E-04 | 3.49E-02 | 1.97 | 3.49E-03 | 1.24 | 2.87E-02 | -0.34 | 5.00E-01 | 1.67 | 9.77E-03 | -0.76 | 1.68E-01 | 1.02 | 7.60E-02 |
| MMP2 | 37.78 | 3.85E-04 | 3.49E-02 | 0.51 | 3.26E-01 | -0.10 | 8.38E-01 | 0.40 | 4.26E-01 | 4.26 | 4.98E-05 | -2.22 | 1.75E-03 | 0.27 | 6.13E-01 |
| ZRANB3 | 37.83 | 3.81E-04 | 3.49E-02 | -0.47 | 3.63E-01 | -0.55 | 2.87E-01 | -0.92 | 8.43E-02 | -1.22 | 4.13E-02 | 1.72 | 8.06E-03 | 2.12 | 2.09E-03 |
| MXRA7 | 37.63 | 4.00E-04 | 3.53E-02 | 1.56 | 1.25E-02 | -0.46 | 3.71E-01 | 0.56 | 2.76E-01 | 1.32 | 3.02E-02 | 0.88 | 1.17E-01 | 2.23 | 1.50E-03 |
| BLVRA | 37.62 | 4.01E-04 | 3.53E-02 | 0.87 | 1.08E-01 | 0.52 | 3.16E-01 | 0.83 | 1.18E-01 | 1.26 | 3.60E-02 | 2.62 | 5.44E-04 | 0.98 | 8.56E-02 |
| PSPH | 37.64 | 3.99E-04 | 3.53E-02 | -2.31 | 1.28E-03 | 1.87 | 2.83E-03 | 0.65 | 2.08E-01 | 1.30 | 3.16E-02 | 0.50 | 3.53E-01 | -0.13 | 8.05E-01 |
| LSM14B | 37.61 | 4.02E-04 | 3.53E-02 | 2.28 | 1.40E-03 | -0.36 | 4.82E-01 | 0.60 | 2.42E-01 | -1.49 | 1.74E-02 | 1.83 | 5.81E-03 | 0.44 | 4.13E-01 |
| COPS4 | 37.20 | 4.57E-04 | 3.55E-02 | -1.82 | 5.59E-03 | -0.36 | 4.80E-01 | 0.50 | 3.25E-01 | -0.93 | 1.04E-01 | 2.25 | 1.65E-03 | 1.11 | 5.59E-02 |
| MGP | 37.25 | 4.52E-04 | 3.55E-02 | -0.16 | 7.54E-01 | -0.20 | 6.98E-01 | 0.39 | 4.36E-01 | 6.17 | 4.75E-06 | 1.73 | 7.91E-03 | 0.03 | 9.48E-01 |
| TRAPPC2L | 37.46 | 4.23E-04 | 3.55E-02 | 3.35 | 1.05E-04 | 0.69 | 1.89E-01 | 1.08 | 4.66E-02 | 0.71 | 2.04E-01 | 0.26 | 6.18E-01 | 1.08 | 6.29E-02 |
| CCL2 | 37.20 | 4.57E-04 | 3.55E-02 | -1.50 | 1.49E-02 | 1.10 | 4.86E-02 | 0.10 | 8.42E-01 | -1.07 | 6.71E-02 | -2.77 | 3.60E-04 | 0.30 | 5.71E-01 |
| PPP2R1B | 37.44 | 4.26E-04 | 3.55E-02 | -4.97 | 3.26E-06 | -0.03 | 9.53E-01 | 0.32 | 5.26E-01 | 0.52 | 3.45E-01 | -1.20 | 4.25E-02 | -0.55 | 3.09E-01 |
| A2M | 37.33 | 4.37E-04 | 3.55E-02 | 0.64 | 2.24E-01 | -0.44 | 3.96E-01 | -0.94 | 8.03E-02 | 5.71 | 1.45E-05 | -0.62 | 2.52E-01 | 0.56 | 3.00E-01 |
| DACT1 | 37.53 | 4.13E-04 | 3.55E-02 | 0.82 | 1.28E-01 | -0.94 | 8.31E-02 | -2.36 | 2.27E-04 | 0.22 | 6.90E-01 | 0.42 | 4.26E-01 | 1.63 | 9.99E-03 |
| PIGM | 37.42 | 4.27E-04 | 3.55E-02 | -2.68 | 4.82E-04 | -0.09 | 8.62E-01 | -0.98 | 6.91E-02 | 1.99 | 3.73E-03 | 0.12 | 8.17E-01 | 0.98 | 8.55E-02 |
| HDGFRP3 | 37.14 | 4.65E-04 | 3.55E-02 | 1.30 | 2.77E-02 | 0.73 | 1.70E-01 | -0.92 | 8.56E-02 | -1.39 | 2.39E-02 | 1.87 | 5.25E-03 | 0.76 | 1.70E-01 |
| TGM2 | 37.40 | 4.31E-04 | 3.55E-02 | 2.25 | 1.56E-03 | -0.51 | 3.24E-01 | -0.40 | 4.24E-01 | 2.39 | 1.30E-03 | -1.07 | 6.41E-02 | -0.43 | 4.24E-01 |
| ALDH1A2 | 37.18 | 4.60E-04 | 3.55E-02 | 1.14 | 4.65E-02 | -0.21 | 6.86E-01 | 0.55 | 2.83E-01 | 1.62 | 1.13E-02 | -1.82 | 6.05E-03 | 1.53 | 1.37E-02 |
| MED24 | 37.26 | 4.50E-04 | 3.55E-02 | 0.57 | 2.74E-01 | -0.21 | 6.84E-01 | -1.68 | 4.12E-03 | 1.80 | 6.67E-03 | -0.86 | 1.22E-01 | 1.56 | 1.28E-02 |
| RFX3 | 37.15 | 4.64E-04 | 3.55E-02 | -0.73 | 1.70E-01 | -0.28 | 5.86E-01 | 2.05 | 8.24E-04 | 1.17 | 4.85E-02 | -1.03 | 7.27E-02 | 1.30 | 2.97E-02 |
| KIF20B | 37.40 | 4.30E-04 | 3.55E-02 | -2.73 | 4.32E-04 | -0.34 | 5.06E-01 | 0.77 | 1.42E-01 | -1.00 | 8.30E-02 | 1.22 | 4.04E-02 | 1.03 | 7.22E-02 |
| KIAA1524 | 37.16 | 4.63E-04 | 3.55E-02 | -0.66 | 2.10E-01 | 0.46 | 3.74E-01 | 0.21 | 6.75E-01 | -2.42 | 1.18E-03 | 1.69 | 9.04E-03 | 1.50 | 1.51E-02 |
| PCDHB16 | 37.24 | 4.52E-04 | 3.55E-02 | -0.28 | 5.82E-01 | -0.53 | 3.11E-01 | -2.96 | 2.11E-05 | 0.85 | 1.36E-01 | -1.02 | 7.62E-02 | 0.69 | 2.07E-01 |
| KAL1 | 37.23 | 4.53E-04 | 3.55E-02 | 1.00 | 7.28E-02 | -0.84 | 1.17E-01 | -0.54 | 2.88E-01 | -1.21 | 4.29E-02 | 1.17 | 4.62E-02 | 2.19 | 1.69E-03 |
| CSTA | 37.23 | 4.53E-04 | 3.55E-02 | 1.91 | 4.22E-03 | 0.87 | 1.07E-01 | 0.53 | 2.94E-01 | -1.10 | 6.09E-02 | 0.84 | 1.30E-01 | -1.71 | 7.87E-03 |
| C20ORF96 | 37.34 | 4.37E-04 | 3.55E-02 | -0.84 | 1.20E-01 | -0.48 | 3.52E-01 | 0.61 | 2.38E-01 | 1.87 | 5.25E-03 | -1.83 | 5.88E-03 | -1.35 | 2.51E-02 |
| MRPS15 | 37.12 | 4.69E-04 | 3.56E-02 | 1.34 | 2.52E-02 | -0.15 | 7.68E-01 | 1.44 | 1.13E-02 | -0.80 | 1.54E-01 | 2.62 | 5.54E-04 | 0.39 | 4.67E-01 |
| CPT1A | 37.08 | 4.74E-04 | 3.58E-02 | 1.00 | 7.35E-02 | 1.16 | 3.78E-02 | -1.22 | 2.69E-02 | 1.23 | 3.96E-02 | -0.02 | 9.74E-01 | 2.00 | 3.07E-03 |
| L3MBTL1 | 37.05 | 4.80E-04 | 3.60E-02 | 1.71 | 7.75E-03 | -0.82 | 1.24E-01 | -2.46 | 1.43E-04 | -0.47 | 3.91E-01 | -0.31 | 5.54E-01 | 0.56 | 3.04E-01 |
| IGFBP3 | 36.98 | 4.93E-04 | 3.67E-02 | 1.09 | 5.44E-02 | -1.56 | 8.87E-03 | -0.59 | 2.50E-01 | 0.56 | 3.10E-01 | 2.91 | 2.55E-04 | 0.01 | 9.80E-01 |
| CCNA2 | 36.96 | 4.96E-04 | 3.67E-02 | -1.39 | 2.13E-02 | 0.44 | 3.97E-01 | -0.60 | 2.46E-01 | 0.38 | 4.83E-01 | 2.07 | 2.77E-03 | 1.97 | 3.39E-03 |
| NDC80 | 36.92 | 5.02E-04 | 3.68E-02 | -2.59 | 6.31E-04 | 0.28 | 5.79E-01 | 0.15 | 7.61E-01 | -0.07 | 8.92E-01 | 2.05 | 2.97E-03 | 1.55 | 1.31E-02 |
| ZNF512 | 36.81 | 5.21E-04 | 3.80E-02 | 0.17 | 7.29E-01 | -0.34 | 5.04E-01 | -1.87 | 1.80E-03 | 1.32 | 3.03E-02 | -0.96 | 9.14E-02 | 1.81 | 5.57E-03 |
| PSMB5 | 36.78 | 5.28E-04 | 3.83E-02 | -0.45 | 3.85E-01 | 0.73 | 1.70E-01 | 0.72 | 1.70E-01 | 0.81 | 1.53E-01 | 2.08 | 2.69E-03 | 2.09 | 2.24E-03 |
| ECM1 | 36.56 | 5.61E-04 | 3.83E-02 | -0.93 | 9.22E-02 | 0.26 | 6.09E-01 | 1.08 | 4.74E-02 | 2.96 | 3.71E-04 | -1.43 | 2.04E-02 | -0.30 | 5.71E-01 |
| NUP37 | 36.59 | 5.58E-04 | 3.83E-02 | 0.63 | 2.32E-01 | 0.06 | 9.00E-01 | 0.94 | 7.90E-02 | -0.63 | 2.60E-01 | 2.60 | 5.82E-04 | 1.87 | 4.55E-03 |
| FAM13B | 36.54 | 5.64E-04 | 3.83E-02 | -1.11 | 5.21E-02 | -0.52 | 3.15E-01 | -1.06 | 5.01E-02 | -2.03 | 3.37E-03 | -1.74 | 7.53E-03 | 0.31 | 5.55E-01 |
| SIAE | 36.57 | 5.59E-04 | 3.83E-02 | -1.04 | 6.41E-02 | -0.35 | 4.97E-01 | -0.41 | 4.12E-01 | 2.04 | 3.28E-03 | 2.13 | 2.40E-03 | 0.90 | 1.10E-01 |
| RPL22L1 | 36.66 | 5.47E-04 | 3.83E-02 | -1.71 | 7.69E-03 | -0.18 | 7.20E-01 | 0.72 | 1.67E-01 | -0.79 | 1.61E-01 | 3.06 | 1.83E-04 | 0.45 | 4.01E-01 |
| VCAM1 | 36.65 | 5.49E-04 | 3.83E-02 | -1.67 | 8.68E-03 | -0.90 | 9.63E-02 | -2.04 | 8.53E-04 | -0.88 | 1.24E-01 | -0.65 | 2.32E-01 | -0.33 | 5.37E-01 |
| RBM14 | 36.52 | 5.69E-04 | 3.83E-02 | -0.82 | 1.27E-01 | 0.05 | 9.17E-01 | -0.63 | 2.22E-01 | 0.97 | 9.19E-02 | -3.39 | 8.75E-05 | -1.10 | 5.69E-02 |
| DENND4A | 36.57 | 5.59E-04 | 3.83E-02 | -0.38 | 4.52E-01 | -0.36 | 4.79E-01 | -1.82 | 2.23E-03 | -2.20 | 2.11E-03 | -1.55 | 1.41E-02 | -0.14 | 7.99E-01 |
| SLC7A8 | 36.73 | 5.36E-04 | 3.83E-02 | 1.46 | 1.68E-02 | 0.47 | 3.60E-01 | 0.94 | 7.81E-02 | 1.52 | 1.58E-02 | -1.69 | 8.87E-03 | -0.78 | 1.61E-01 |
| KRT19 | 36.53 | 5.66E-04 | 3.83E-02 | -0.06 | 9.11E-01 | 0.34 | 5.07E-01 | -0.05 | 9.20E-01 | 2.37 | 1.37E-03 | 1.79 | 6.63E-03 | -2.00 | 3.02E-03 |
| SLC6A6 | 36.66 | 5.47E-04 | 3.83E-02 | 1.60 | 1.09E-02 | -0.12 | 8.14E-01 | -1.07 | 4.88E-02 | 2.59 | 8.02E-04 | -0.69 | 2.08E-01 | -0.80 | 1.52E-01 |
| HLF | 36.74 | 5.34E-04 | 3.83E-02 | -0.99 | 7.64E-02 | -0.19 | 7.11E-01 | -0.66 | 2.04E-01 | -0.15 | 7.78E-01 | -2.81 | 3.33E-04 | -1.94 | 3.67E-03 |
| SF1 | 36.48 | 5.78E-04 | 3.87E-02 | 1.02 | 6.89E-02 | -1.34 | 1.97E-02 | -0.70 | 1.78E-01 | 1.47 | 1.87E-02 | -1.94 | 4.15E-03 | -0.25 | 6.41E-01 |
| TRMT61B | 36.40 | 5.88E-04 | 3.87E-02 | -0.12 | 8.08E-01 | 0.04 | 9.33E-01 | -0.44 | 3.87E-01 | -1.53 | 1.55E-02 | 2.13 | 2.36E-03 | 2.31 | 1.17E-03 |
| NUP93 | 36.41 | 5.86E-04 | 3.87E-02 | -0.63 | 2.27E-01 | 0.49 | 3.40E-01 | -1.55 | 7.09E-03 | -0.14 | 7.89E-01 | 1.59 | 1.23E-02 | 2.08 | 2.33E-03 |
| FAM65B | 36.44 | 5.84E-04 | 3.87E-02 | -2.22 | 1.69E-03 | -1.85 | 3.05E-03 | -0.92 | 8.63E-02 | 0.71 | 2.05E-01 | 0.69 | 2.07E-01 | 0.24 | 6.48E-01 |
| SRSF6 | 36.30 | 6.10E-04 | 3.98E-02 | 1.87 | 4.71E-03 | -0.13 | 7.94E-01 | -1.41 | 1.27E-02 | 0.91 | 1.12E-01 | -1.71 | 8.51E-03 | -0.58 | 2.87E-01 |
| S100A8 | 36.24 | 6.22E-04 | 4.04E-02 | -0.64 | 2.21E-01 | 1.29 | 2.38E-02 | 0.27 | 5.89E-01 | -1.52 | 1.56E-02 | -1.07 | 6.45E-02 | -1.89 | 4.35E-03 |
| SURF4 | 36.21 | 6.28E-04 | 4.04E-02 | 2.95 | 2.55E-04 | 0.31 | 5.50E-01 | -0.56 | 2.75E-01 | 1.74 | 8.04E-03 | -0.57 | 2.91E-01 | 0.80 | 1.52E-01 |
| WDR1 | 36.15 | 6.41E-04 | 4.04E-02 | 2.68 | 4.85E-04 | -0.88 | 1.03E-01 | -0.92 | 8.63E-02 | 0.80 | 1.55E-01 | 0.83 | 1.39E-01 | 0.79 | 1.54E-01 |
| SETD7 | 36.15 | 6.41E-04 | 4.04E-02 | -2.54 | 7.07E-04 | 0.29 | 5.69E-01 | -0.71 | 1.72E-01 | 0.93 | 1.06E-01 | -1.99 | 3.49E-03 | 0.32 | 5.50E-01 |
| CBL | 36.14 | 6.42E-04 | 4.04E-02 | 2.00 | 3.13E-03 | -0.53 | 3.09E-01 | -0.93 | 8.21E-02 | -1.18 | 4.71E-02 | -0.96 | 9.19E-02 | 1.20 | 4.12E-02 |
| GABPB1 | 36.12 | 6.45E-04 | 4.04E-02 | 1.65 | 9.33E-03 | 0.47 | 3.64E-01 | -0.92 | 8.43E-02 | -1.21 | 4.32E-02 | 0.40 | 4.56E-01 | 2.06 | 2.54E-03 |
| INTS3 | 36.10 | 6.48E-04 | 4.04E-02 | 0.28 | 5.83E-01 | -0.84 | 1.19E-01 | -1.10 | 4.44E-02 | 1.83 | 6.06E-03 | -1.11 | 5.63E-02 | 1.53 | 1.38E-02 |
| EMCN | 36.15 | 6.41E-04 | 4.04E-02 | -2.97 | 2.44E-04 | 0.14 | 7.76E-01 | -1.66 | 4.33E-03 | 1.03 | 7.65E-02 | 0.51 | 3.45E-01 | 0.24 | 6.52E-01 |
| ATP5C1 | 36.05 | 6.58E-04 | 4.08E-02 | -0.65 | 2.17E-01 | -0.34 | 5.11E-01 | 0.61 | 2.36E-01 | -0.82 | 1.46E-01 | 2.20 | 1.83E-03 | 2.11 | 2.12E-03 |
| SARS | 36.04 | 6.62E-04 | 4.08E-02 | 1.66 | 8.99E-03 | 0.92 | 9.11E-02 | -0.04 | 9.36E-01 | 2.13 | 2.55E-03 | -0.57 | 2.94E-01 | 1.34 | 2.60E-02 |
| NUDT9 | 35.97 | 6.77E-04 | 4.11E-02 | -1.22 | 3.59E-02 | -0.73 | 1.70E-01 | -0.60 | 2.43E-01 | -0.48 | 3.78E-01 | 1.12 | 5.42E-02 | 2.60 | 5.10E-04 |
| ADAMTS5 | 36.00 | 6.71E-04 | 4.11E-02 | -0.68 | 1.99E-01 | -0.71 | 1.80E-01 | -1.21 | 2.87E-02 | 1.05 | 7.14E-02 | 1.61 | 1.14E-02 | 1.45 | 1.82E-02 |
| SIRT4 | 35.97 | 6.77E-04 | 4.11E-02 | 0.91 | 9.75E-02 | -0.85 | 1.15E-01 | 0.55 | 2.84E-01 | -1.73 | 8.30E-03 | 0.67 | 2.18E-01 | -2.04 | 2.70E-03 |
| VPS25 | 35.91 | 6.87E-04 | 4.13E-02 | -0.38 | 4.62E-01 | 0.58 | 2.68E-01 | 0.54 | 2.89E-01 | 2.72 | 6.07E-04 | 0.94 | 9.70E-02 | 1.72 | 7.53E-03 |
| C15ORF61 | 35.88 | 6.94E-04 | 4.13E-02 | 1.16 | 4.41E-02 | -0.03 | 9.48E-01 | 0.92 | 8.63E-02 | -1.22 | 4.21E-02 | 2.49 | 8.06E-04 | 0.84 | 1.32E-01 |
| KIAA0101 | 35.89 | 6.91E-04 | 4.13E-02 | -0.95 | 8.57E-02 | 0.88 | 1.04E-01 | 0.86 | 1.07E-01 | -0.06 | 9.17E-01 | 2.31 | 1.37E-03 | 1.54 | 1.35E-02 |
| SPCS2 | 35.89 | 6.90E-04 | 4.13E-02 | -1.32 | 2.68E-02 | 0.62 | 2.35E-01 | -1.41 | 1.26E-02 | 0.42 | 4.44E-01 | 1.65 | 1.02E-02 | -1.18 | 4.49E-02 |
| IRF1 | 35.86 | 7.01E-04 | 4.15E-02 | 1.59 | 1.12E-02 | -0.55 | 2.87E-01 | -1.06 | 5.03E-02 | 0.42 | 4.44E-01 | -2.42 | 1.02E-03 | -0.67 | 2.24E-01 |
| HIVEP2 | 35.80 | 7.15E-04 | 4.20E-02 | -2.24 | 1.58E-03 | -0.08 | 8.72E-01 | -1.76 | 2.87E-03 | -1.26 | 3.66E-02 | -0.70 | 2.03E-01 | -0.30 | 5.73E-01 |
| ARPC4 | 35.76 | 7.23E-04 | 4.21E-02 | 1.14 | 4.61E-02 | 0.47 | 3.62E-01 | 1.47 | 9.87E-03 | 1.68 | 9.55E-03 | 0.13 | 8.02E-01 | 1.54 | 1.36E-02 |
| KRT80 | 35.77 | 7.20E-04 | 4.21E-02 | 1.77 | 6.46E-03 | -1.12 | 4.53E-02 | 1.79 | 2.51E-03 | 0.09 | 8.62E-01 | -0.46 | 3.86E-01 | -1.04 | 6.99E-02 |
| IER2 | 35.70 | 7.38E-04 | 4.21E-02 | 1.02 | 6.89E-02 | 0.07 | 8.91E-01 | -0.51 | 3.15E-01 | 1.66 | 1.01E-02 | -1.98 | 3.64E-03 | -1.35 | 2.49E-02 |
| DDX26B | 35.72 | 7.31E-04 | 4.21E-02 | -0.52 | 3.17E-01 | -0.34 | 5.07E-01 | -2.22 | 4.15E-04 | -2.15 | 2.41E-03 | -0.84 | 1.34E-01 | -0.13 | 8.13E-01 |
| COG7 | 35.72 | 7.32E-04 | 4.21E-02 | 0.14 | 7.75E-01 | 0.32 | 5.28E-01 | -1.39 | 1.40E-02 | 2.76 | 5.43E-04 | -1.54 | 1.47E-02 | 0.47 | 3.84E-01 |
| TP53 | 35.69 | 7.39E-04 | 4.21E-02 | 2.49 | 7.92E-04 | -0.46 | 3.75E-01 | 1.06 | 5.13E-02 | 0.82 | 1.48E-01 | -1.38 | 2.42E-02 | -0.53 | 3.26E-01 |
| C14ORF2 | 35.68 | 7.43E-04 | 4.21E-02 | -1.02 | 6.79E-02 | 0.73 | 1.71E-01 | 1.22 | 2.74E-02 | -0.36 | 5.02E-01 | 2.49 | 8.02E-04 | 0.83 | 1.40E-01 |
| ENPP2 | 35.64 | 7.54E-04 | 4.25E-02 | 0.46 | 3.70E-01 | -0.98 | 7.40E-02 | -0.79 | 1.32E-01 | 1.82 | 6.28E-03 | -1.46 | 1.89E-02 | -1.19 | 4.26E-02 |
| FABP3 | 35.62 | 7.57E-04 | 4.25E-02 | -1.03 | 6.64E-02 | -1.33 | 2.07E-02 | 0.89 | 9.60E-02 | -0.68 | 2.23E-01 | 0.75 | 1.76E-01 | 1.95 | 3.54E-03 |
| GPNMB | 35.61 | 7.64E-04 | 4.27E-02 | -0.46 | 3.75E-01 | 0.04 | 9.32E-01 | 0.49 | 3.37E-01 | 3.63 | 1.36E-04 | 1.01 | 7.88E-02 | 1.51 | 1.48E-02 |
| C14ORF166 | 35.54 | 7.76E-04 | 4.27E-02 | -0.72 | 1.76E-01 | -0.35 | 4.89E-01 | 0.40 | 4.24E-01 | -0.81 | 1.51E-01 | 2.70 | 4.34E-04 | 1.70 | 8.03E-03 |
| ZBTB45 | 35.57 | 7.69E-04 | 4.27E-02 | 3.82 | 3.98E-05 | -0.33 | 5.20E-01 | 0.96 | 7.48E-02 | 1.26 | 3.67E-02 | 0.36 | 4.99E-01 | -0.23 | 6.68E-01 |
| C20ORF85 | 35.54 | 7.76E-04 | 4.27E-02 | 0.62 | 2.39E-01 | 1.31 | 2.20E-02 | 1.14 | 3.71E-02 | 2.87 | 4.44E-04 | 0.18 | 7.27E-01 | 0.56 | 3.04E-01 |
| PNPT1 | 35.50 | 7.82E-04 | 4.29E-02 | -0.47 | 3.59E-01 | 0.61 | 2.44E-01 | -1.52 | 7.95E-03 | -0.55 | 3.19E-01 | 0.93 | 1.01E-01 | 2.41 | 8.69E-04 |
| CCNL1 | 35.48 | 7.88E-04 | 4.30E-02 | -0.72 | 1.75E-01 | 0.74 | 1.64E-01 | -1.64 | 4.81E-03 | -0.14 | 7.93E-01 | -1.96 | 3.86E-03 | -1.17 | 4.66E-02 |
| ACTB | 35.42 | 8.02E-04 | 4.30E-02 | 1.41 | 1.96E-02 | 0.03 | 9.52E-01 | -1.25 | 2.44E-02 | 2.69 | 6.43E-04 | -0.49 | 3.61E-01 | 0.72 | 1.92E-01 |
| BCAT1 | 35.45 | 7.95E-04 | 4.30E-02 | 1.64 | 9.61E-03 | -0.79 | 1.39E-01 | 0.79 | 1.34E-01 | -1.57 | 1.34E-02 | 1.64 | 1.05E-02 | -0.14 | 7.99E-01 |
| ABHD2 | 35.42 | 8.02E-04 | 4.30E-02 | 3.43 | 8.63E-05 | -0.42 | 4.16E-01 | -0.82 | 1.19E-01 | 1.05 | 7.05E-02 | -0.93 | 1.01E-01 | 0.23 | 6.68E-01 |
| APCDD1 | 35.40 | 8.07E-04 | 4.30E-02 | 0.63 | 2.28E-01 | 0.37 | 4.70E-01 | -0.07 | 8.88E-01 | 2.92 | 4.01E-04 | -1.77 | 6.95E-03 | -1.01 | 7.76E-02 |
| ZNF709 | 35.40 | 8.08E-04 | 4.30E-02 | 0.24 | 6.29E-01 | 0.58 | 2.67E-01 | -1.03 | 5.73E-02 | 1.45 | 1.98E-02 | -1.32 | 2.94E-02 | -1.94 | 3.67E-03 |
| RABEPK | 35.37 | 8.14E-04 | 4.31E-02 | 0.97 | 7.99E-02 | 0.11 | 8.23E-01 | 0.45 | 3.72E-01 | -0.82 | 1.45E-01 | 2.25 | 1.63E-03 | 1.95 | 3.61E-03 |
| NDUFAB1 | 35.31 | 8.29E-04 | 4.33E-02 | -0.68 | 2.00E-01 | -0.08 | 8.72E-01 | 0.70 | 1.79E-01 | -1.18 | 4.72E-02 | 2.59 | 6.10E-04 | 1.37 | 2.40E-02 |
| NASP | 35.33 | 8.25E-04 | 4.33E-02 | -2.23 | 1.66E-03 | -0.66 | 2.12E-01 | -1.41 | 1.29E-02 | -0.93 | 1.03E-01 | -0.73 | 1.82E-01 | -0.63 | 2.49E-01 |
| PHF1 | 35.31 | 8.28E-04 | 4.33E-02 | -1.39 | 2.14E-02 | -0.48 | 3.57E-01 | 0.67 | 1.95E-01 | 1.37 | 2.52E-02 | -2.39 | 1.12E-03 | -0.35 | 5.10E-01 |
| CDK5 | 35.25 | 8.47E-04 | 4.37E-02 | 0.33 | 5.22E-01 | 0.21 | 6.73E-01 | 2.14 | 5.69E-04 | 1.95 | 4.24E-03 | -0.91 | 1.06E-01 | 0.63 | 2.47E-01 |
| C9ORF131 | 35.25 | 8.46E-04 | 4.37E-02 | 0.28 | 5.76E-01 | 0.13 | 7.96E-01 | -0.11 | 8.16E-01 | -2.60 | 7.87E-04 | -2.52 | 7.44E-04 | -0.93 | 1.01E-01 |
| HOXB3 | 35.24 | 8.48E-04 | 4.37E-02 | -0.89 | 1.03E-01 | -0.37 | 4.73E-01 | -1.27 | 2.22E-02 | 1.47 | 1.86E-02 | -1.36 | 2.58E-02 | -1.19 | 4.31E-02 |
| CYTH3 | 35.21 | 8.57E-04 | 4.40E-02 | 2.29 | 1.37E-03 | -1.02 | 6.40E-02 | -0.71 | 1.75E-01 | 0.30 | 5.79E-01 | -1.02 | 7.45E-02 | -1.26 | 3.43E-02 |
| CACHD1 | 35.15 | 8.71E-04 | 4.44E-02 | -0.15 | 7.73E-01 | -0.39 | 4.42E-01 | -0.88 | 9.75E-02 | -1.27 | 3.51E-02 | -2.57 | 6.32E-04 | -1.28 | 3.15E-02 |
| DHRS3 | 35.12 | 8.78E-04 | 4.44E-02 | -1.03 | 6.60E-02 | -1.46 | 1.30E-02 | 0.36 | 4.72E-01 | 2.09 | 2.78E-03 | 1.20 | 4.26E-02 | 0.36 | 4.95E-01 |
| PIK3R3 | 35.12 | 8.76E-04 | 4.44E-02 | 1.39 | 2.12E-02 | 0.76 | 1.55E-01 | -1.36 | 1.53E-02 | -1.50 | 1.70E-02 | -0.68 | 2.13E-01 | -0.85 | 1.29E-01 |
| CA5B | 35.08 | 8.87E-04 | 4.45E-02 | -0.78 | 1.43E-01 | 0.85 | 1.14E-01 | 1.69 | 3.89E-03 | 0.91 | 1.12E-01 | -0.07 | 8.92E-01 | 1.93 | 3.79E-03 |
| SLC10A1 | 35.06 | 8.91E-04 | 4.45E-02 | 0.67 | 2.04E-01 | -0.49 | 3.41E-01 | 0.11 | 8.26E-01 | -2.23 | 1.95E-03 | -2.51 | 7.54E-04 | -0.58 | 2.89E-01 |
| ARMC9 | 35.07 | 8.90E-04 | 4.45E-02 | -1.36 | 2.35E-02 | 0.69 | 1.92E-01 | -1.48 | 9.26E-03 | 2.15 | 2.40E-03 | 0.48 | 3.72E-01 | -0.24 | 6.52E-01 |
| CISD1 | 35.01 | 9.04E-04 | 4.48E-02 | -1.55 | 1.28E-02 | -0.32 | 5.25E-01 | 0.98 | 6.86E-02 | -0.60 | 2.78E-01 | 2.47 | 8.84E-04 | 0.68 | 2.19E-01 |
| DDX50 | 34.98 | 9.13E-04 | 4.48E-02 | -0.34 | 5.07E-01 | -1.01 | 6.63E-02 | -0.06 | 9.01E-01 | -1.05 | 7.22E-02 | 2.63 | 5.31E-04 | 1.39 | 2.19E-02 |
| UQCR10 | 34.99 | 9.10E-04 | 4.48E-02 | 0.46 | 3.73E-01 | 0.69 | 1.89E-01 | 1.17 | 3.37E-02 | 0.84 | 1.40E-01 | 2.18 | 2.03E-03 | 1.23 | 3.75E-02 |
| ATRX | 35.00 | 9.09E-04 | 4.48E-02 | -1.38 | 2.21E-02 | -0.67 | 2.04E-01 | -1.43 | 1.18E-02 | -1.82 | 6.27E-03 | 0.85 | 1.30E-01 | -0.29 | 5.82E-01 |
| RBM5 | 34.94 | 9.27E-04 | 4.53E-02 | 0.33 | 5.19E-01 | -1.02 | 6.45E-02 | -1.38 | 1.44E-02 | 0.76 | 1.74E-01 | -1.65 | 1.03E-02 | -1.30 | 3.01E-02 |
| QRSL1 | 34.93 | 9.31E-04 | 4.53E-02 | -0.66 | 2.09E-01 | 0.61 | 2.45E-01 | -1.27 | 2.23E-02 | 1.31 | 3.05E-02 | 0.75 | 1.72E-01 | 1.89 | 4.36E-03 |
| TMBIM4 | 34.87 | 9.45E-04 | 4.56E-02 | -0.25 | 6.19E-01 | 0.99 | 7.05E-02 | 0.79 | 1.35E-01 | -0.63 | 2.57E-01 | 2.03 | 3.17E-03 | 1.81 | 5.57E-03 |
| SLC2A1 | 34.88 | 9.42E-04 | 4.56E-02 | 0.50 | 3.31E-01 | 0.06 | 9.11E-01 | 1.20 | 2.98E-02 | 2.86 | 4.49E-04 | -1.28 | 3.33E-02 | -0.71 | 1.99E-01 |
| ANKZF1 | 34.86 | 9.50E-04 | 4.56E-02 | -0.96 | 8.43E-02 | -0.62 | 2.39E-01 | -1.55 | 7.13E-03 | 2.01 | 3.52E-03 | -1.10 | 5.91E-02 | -0.07 | 9.02E-01 |
| EIF2B3 | 34.80 | 9.70E-04 | 4.60E-02 | 0.25 | 6.20E-01 | 0.65 | 2.19E-01 | 0.59 | 2.49E-01 | -0.46 | 3.96E-01 | 1.92 | 4.35E-03 | 2.61 | 4.79E-04 |
| CLTA | 34.76 | 9.80E-04 | 4.60E-02 | 2.05 | 2.77E-03 | 0.36 | 4.79E-01 | 0.92 | 8.39E-02 | -1.09 | 6.39E-02 | 1.30 | 3.06E-02 | -0.85 | 1.30E-01 |
| ELP4 | 34.80 | 9.68E-04 | 4.60E-02 | 0.06 | 9.05E-01 | 0.29 | 5.64E-01 | 0.97 | 7.01E-02 | -0.98 | 8.86E-02 | 1.76 | 7.17E-03 | 2.29 | 1.22E-03 |
| ZNF124 | 34.78 | 9.77E-04 | 4.60E-02 | -1.03 | 6.60E-02 | -0.98 | 7.20E-02 | 0.93 | 8.19E-02 | 0.82 | 1.46E-01 | -1.02 | 7.53E-02 | 1.76 | 6.55E-03 |
| CENPK | 34.74 | 9.86E-04 | 4.60E-02 | -1.53 | 1.36E-02 | 0.77 | 1.49E-01 | -1.04 | 5.50E-02 | -0.04 | 9.43E-01 | 1.98 | 3.72E-03 | 1.03 | 7.31E-02 |
| POLR3G | 34.74 | 9.87E-04 | 4.60E-02 | -1.49 | 1.54E-02 | 0.82 | 1.27E-01 | -0.51 | 3.15E-01 | -1.83 | 6.01E-03 | 0.35 | 5.07E-01 | -1.50 | 1.53E-02 |
| SP110 | 34.75 | 9.83E-04 | 4.60E-02 | -1.97 | 3.52E-03 | 0.83 | 1.21E-01 | -0.99 | 6.54E-02 | -0.69 | 2.16E-01 | 1.61 | 1.13E-02 | 0.43 | 4.18E-01 |
| NDUFB8 | 34.69 | 1.00E-03 | 4.60E-02 | -1.05 | 6.29E-02 | -0.74 | 1.65E-01 | 0.80 | 1.27E-01 | -0.59 | 2.83E-01 | 2.15 | 2.21E-03 | 1.24 | 3.57E-02 |
| CRIPAK | 34.69 | 1.00E-03 | 4.60E-02 | -0.60 | 2.53E-01 | -0.63 | 2.33E-01 | -1.99 | 1.09E-03 | -1.80 | 6.70E-03 | -0.53 | 3.25E-01 | 0.69 | 2.10E-01 |
| GFRA2 | 34.72 | 9.93E-04 | 4.60E-02 | -0.91 | 9.61E-02 | 0.10 | 8.48E-01 | 1.01 | 6.18E-02 | 0.24 | 6.55E-01 | -2.02 | 3.28E-03 | -2.04 | 2.67E-03 |
| FGF7 | 34.69 | 1.00E-03 | 4.60E-02 | -0.70 | 1.84E-01 | -0.38 | 4.56E-01 | -1.32 | 1.83E-02 | -0.70 | 2.09E-01 | 3.04 | 1.88E-04 | 0.37 | 4.87E-01 |
| GTF3A | 34.65 | 1.02E-03 | 4.64E-02 | 0.23 | 6.44E-01 | 0.33 | 5.15E-01 | 0.79 | 1.34E-01 | -1.06 | 6.95E-02 | 2.47 | 8.82E-04 | 1.60 | 1.10E-02 |
| SPARCL1 | 34.65 | 1.02E-03 | 4.64E-02 | 0.49 | 3.40E-01 | -0.15 | 7.60E-01 | -0.97 | 6.98E-02 | 4.18 | 5.73E-05 | -0.86 | 1.23E-01 | -0.65 | 2.35E-01 |
| DHX35 | 34.63 | 1.02E-03 | 4.64E-02 | 1.71 | 7.82E-03 | -0.64 | 2.20E-01 | -1.40 | 1.31E-02 | 0.42 | 4.38E-01 | -0.79 | 1.56E-01 | 1.42 | 1.97E-02 |
| NDUFA4 | 34.59 | 1.04E-03 | 4.65E-02 | -1.52 | 1.40E-02 | 0.10 | 8.45E-01 | 1.18 | 3.27E-02 | -0.46 | 3.95E-01 | 1.96 | 3.90E-03 | 1.13 | 5.16E-02 |
| ATL3 | 34.59 | 1.04E-03 | 4.65E-02 | 1.06 | 6.05E-02 | 0.97 | 7.64E-02 | 1.30 | 1.96E-02 | 0.94 | 1.02E-01 | 0.34 | 5.27E-01 | 1.77 | 6.33E-03 |
| ELOF1 | 34.57 | 1.04E-03 | 4.65E-02 | -0.48 | 3.54E-01 | 0.28 | 5.84E-01 | 1.35 | 1.62E-02 | 2.73 | 5.77E-04 | -1.36 | 2.56E-02 | 0.26 | 6.31E-01 |
| GNB4 | 34.57 | 1.05E-03 | 4.65E-02 | 1.57 | 1.19E-02 | -0.55 | 2.94E-01 | -0.14 | 7.72E-01 | 0.47 | 3.87E-01 | 1.33 | 2.79E-02 | 2.34 | 1.07E-03 |
| ARMC3 | 34.61 | 1.03E-03 | 4.65E-02 | -1.28 | 3.01E-02 | 0.21 | 6.74E-01 | 1.76 | 2.87E-03 | 1.29 | 3.33E-02 | -0.66 | 2.28E-01 | 1.05 | 6.92E-02 |
| NDUFB6 | 34.52 | 1.06E-03 | 4.69E-02 | -0.85 | 1.16E-01 | -0.25 | 6.25E-01 | 1.11 | 4.21E-02 | 0.36 | 5.07E-01 | 2.56 | 6.50E-04 | 1.28 | 3.17E-02 |
| TSHZ3 | 34.51 | 1.06E-03 | 4.69E-02 | 1.26 | 3.17E-02 | -0.10 | 8.46E-01 | -1.39 | 1.37E-02 | 2.31 | 1.62E-03 | -0.19 | 7.13E-01 | 1.02 | 7.58E-02 |
| MORC4 | 34.47 | 1.08E-03 | 4.69E-02 | 0.38 | 4.62E-01 | -0.54 | 2.97E-01 | -0.49 | 3.31E-01 | 0.02 | 9.78E-01 | 1.04 | 7.12E-02 | 4.01 | 1.04E-05 |
| MDH1B | 34.47 | 1.08E-03 | 4.69E-02 | 0.59 | 2.56E-01 | 0.54 | 3.01E-01 | 0.62 | 2.31E-01 | 1.60 | 1.23E-02 | 1.55 | 1.43E-02 | 1.62 | 1.04E-02 |
| GDF9 | 34.47 | 1.08E-03 | 4.69E-02 | 0.32 | 5.30E-01 | 0.05 | 9.23E-01 | 0.69 | 1.83E-01 | -3.29 | 2.17E-04 | -2.02 | 3.22E-03 | -0.34 | 5.23E-01 |
| ANAPC11 | 34.45 | 1.08E-03 | 4.70E-02 | 0.99 | 7.65E-02 | 0.37 | 4.70E-01 | 1.17 | 3.28E-02 | 1.83 | 6.10E-03 | 0.35 | 5.08E-01 | 1.66 | 9.03E-03 |
| VDAC3 | 34.37 | 1.11E-03 | 4.75E-02 | -1.77 | 6.38E-03 | -0.31 | 5.38E-01 | 0.17 | 7.35E-01 | -0.01 | 9.78E-01 | 1.41 | 2.20E-02 | 2.51 | 6.33E-04 |
| PYGL | 34.40 | 1.10E-03 | 4.75E-02 | -0.12 | 8.07E-01 | 0.49 | 3.42E-01 | -0.90 | 9.19E-02 | -1.05 | 7.14E-02 | 2.81 | 3.38E-04 | 1.11 | 5.56E-02 |
| COL1A1 | 34.37 | 1.11E-03 | 4.75E-02 | 2.53 | 7.17E-04 | 0.48 | 3.54E-01 | -0.16 | 7.39E-01 | 2.32 | 1.54E-03 | -0.79 | 1.54E-01 | 0.15 | 7.72E-01 |
| TMC4 | 34.37 | 1.11E-03 | 4.75E-02 | 0.26 | 6.11E-01 | 0.02 | 9.76E-01 | -2.30 | 2.91E-04 | 0.97 | 9.26E-02 | -1.03 | 7.37E-02 | -1.31 | 2.91E-02 |
| DOCK1 | 34.34 | 1.12E-03 | 4.77E-02 | -1.66 | 8.95E-03 | 0.89 | 1.01E-01 | -1.70 | 3.64E-03 | 1.06 | 6.84E-02 | -0.23 | 6.58E-01 | 0.65 | 2.36E-01 |
| PRDM1 | 34.33 | 1.13E-03 | 4.77E-02 | 0.39 | 4.51E-01 | 0.64 | 2.20E-01 | -2.06 | 7.90E-04 | 1.38 | 2.47E-02 | -0.90 | 1.10E-01 | -0.77 | 1.65E-01 |
| LIN9 | 34.29 | 1.13E-03 | 4.79E-02 | -1.05 | 6.20E-02 | 0.29 | 5.67E-01 | 0.58 | 2.60E-01 | -0.76 | 1.75E-01 | 1.69 | 9.06E-03 | 2.07 | 2.47E-03 |
| GADD45B | 34.28 | 1.14E-03 | 4.80E-02 | 0.07 | 8.89E-01 | 0.79 | 1.38E-01 | -0.53 | 2.97E-01 | 0.58 | 2.90E-01 | -2.14 | 2.31E-03 | -2.23 | 1.48E-03 |
| CDK6 | 34.24 | 1.15E-03 | 4.82E-02 | 4.30 | 1.60E-05 | -0.49 | 3.46E-01 | -0.91 | 8.97E-02 | -0.08 | 8.81E-01 | 0.47 | 3.77E-01 | 0.67 | 2.22E-01 |
| FREM1 | 34.24 | 1.15E-03 | 4.82E-02 | -1.82 | 5.53E-03 | -0.79 | 1.37E-01 | -0.98 | 6.90E-02 | 1.99 | 3.77E-03 | -0.69 | 2.08E-01 | -0.07 | 8.96E-01 |
| KARS | 34.12 | 1.19E-03 | 4.85E-02 | 1.18 | 4.16E-02 | -0.15 | 7.68E-01 | 0.14 | 7.70E-01 | 0.31 | 5.64E-01 | 2.05 | 2.99E-03 | 2.38 | 9.39E-04 |
| NID1 | 34.13 | 1.19E-03 | 4.85E-02 | 1.67 | 8.85E-03 | 0.21 | 6.86E-01 | -0.41 | 4.12E-01 | 3.07 | 3.03E-04 | -0.80 | 1.50E-01 | 0.51 | 3.43E-01 |
| MTF2 | 34.14 | 1.19E-03 | 4.85E-02 | -0.25 | 6.22E-01 | -0.83 | 1.22E-01 | -1.58 | 6.25E-03 | 0.71 | 2.04E-01 | -1.49 | 1.70E-02 | 1.37 | 2.35E-02 |
| IKBKB | 34.13 | 1.19E-03 | 4.85E-02 | 1.87 | 4.72E-03 | -0.33 | 5.22E-01 | -1.67 | 4.14E-03 | 0.55 | 3.22E-01 | -0.95 | 9.43E-02 | 0.86 | 1.26E-01 |
| MTRF1 | 34.21 | 1.17E-03 | 4.85E-02 | -1.64 | 9.61E-03 | -0.30 | 5.56E-01 | -1.34 | 1.72E-02 | -0.59 | 2.87E-01 | 0.85 | 1.26E-01 | 1.60 | 1.12E-02 |
| SGOL1 | 34.15 | 1.18E-03 | 4.85E-02 | -2.20 | 1.78E-03 | -0.74 | 1.65E-01 | 0.89 | 9.58E-02 | -0.98 | 9.03E-02 | 1.36 | 2.60E-02 | 0.29 | 5.81E-01 |
| RPS6KA6 | 34.15 | 1.19E-03 | 4.85E-02 | -2.55 | 6.85E-04 | -0.45 | 3.78E-01 | -0.07 | 8.86E-01 | -2.01 | 3.49E-03 | -0.55 | 3.03E-01 | 0.79 | 1.58E-01 |
| UBXN2A | 34.11 | 1.20E-03 | 4.85E-02 | -0.71 | 1.79E-01 | -0.47 | 3.65E-01 | 0.61 | 2.38E-01 | -1.60 | 1.21E-02 | 0.90 | 1.09E-01 | 2.15 | 1.91E-03 |
| POLDIP2 | 34.09 | 1.20E-03 | 4.87E-02 | 2.31 | 1.27E-03 | 0.65 | 2.15E-01 | 0.44 | 3.84E-01 | 0.86 | 1.32E-01 | -0.10 | 8.48E-01 | 1.97 | 3.37E-03 |
| ANKRD12 | 34.07 | 1.21E-03 | 4.87E-02 | -0.64 | 2.21E-01 | -1.35 | 1.91E-02 | -1.56 | 6.67E-03 | -1.78 | 7.01E-03 | -0.20 | 7.06E-01 | -0.58 | 2.86E-01 |
| C1S | 34.06 | 1.22E-03 | 4.88E-02 | -0.94 | 8.86E-02 | -0.66 | 2.11E-01 | -0.81 | 1.24E-01 | 3.58 | 1.40E-04 | -0.48 | 3.72E-01 | 0.52 | 3.32E-01 |
| TCTEX1D2 | 34.04 | 1.22E-03 | 4.88E-02 | 0.14 | 7.88E-01 | -0.32 | 5.36E-01 | 1.81 | 2.27E-03 | 0.61 | 2.68E-01 | 2.68 | 4.59E-04 | 0.51 | 3.45E-01 |
| PIAS3 | 34.03 | 1.23E-03 | 4.88E-02 | 0.84 | 1.22E-01 | 0.30 | 5.59E-01 | -1.29 | 2.09E-02 | 1.60 | 1.21E-02 | -1.89 | 4.82E-03 | -0.36 | 4.95E-01 |
| GALNT10 | 34.03 | 1.23E-03 | 4.88E-02 | 0.90 | 1.00E-01 | -0.92 | 8.92E-02 | -1.93 | 1.36E-03 | 0.59 | 2.86E-01 | -0.93 | 9.87E-02 | -0.88 | 1.19E-01 |
| TTC27 | 33.96 | 1.25E-03 | 4.96E-02 | 0.38 | 4.58E-01 | 0.95 | 8.17E-02 | -0.95 | 7.75E-02 | -1.66 | 9.98E-03 | 1.44 | 1.99E-02 | 1.03 | 7.32E-02 |
| UBE2F | 33.94 | 1.26E-03 | 4.98E-02 | 1.55 | 1.27E-02 | 0.45 | 3.80E-01 | 0.21 | 6.66E-01 | -0.66 | 2.39E-01 | 1.68 | 9.22E-03 | 1.79 | 6.02E-03 |
| CAMK2D | 33.92 | 1.27E-03 | 4.99E-02 | 2.11 | 2.31E-03 | -1.37 | 1.75E-02 | -0.44 | 3.86E-01 | 0.63 | 2.56E-01 | 0.74 | 1.79E-01 | 1.09 | 6.00E-02 |
| RXRB | 33.93 | 1.27E-03 | 4.99E-02 | 1.23 | 3.53E-02 | -0.09 | 8.56E-01 | 0.22 | 6.52E-01 | 3.07 | 3.01E-04 | -1.63 | 1.10E-02 | -0.23 | 6.61E-01 |

**Table S3. The result of DAVID gene ontology (GO) enrichment for 869 DEGs from Muscle2.**

| **GO_term** | **GO_category** | **GO_rank** | **Num_of_symbols_in_list_in_GO** | **Percentage_of_symbols_in_list** | **Num_of _symbols_in_bglist_in_GO** | **Num_of_symbols_in_list** | **Num_of_symbols_in_bglist** | **Symbols_in_list** | **Fold_enrichment** | **P value** | **FDR** |
| --- | --- | --- | --- | --- | --- | --- | --- | --- | --- | --- | --- |
| GO:0043292~contractile fiber | CC_FAT | 1 | 17 | 0.0241 | 489 | 121 | 12782 | OBSCN, MYL4, MYL5, MYH1, SYNC, PDLIM3, MYH4, MYOZ2, MYH8, TNNI2, VCL, JUP, TNNT1, ANKRD23, TRIM32, MYH13, PLEC | 3.6724 | 1.41 E-05 | 0.0197 |
| GO:0044449~contractile fiber part | CC_FAT | 2 | 16 | 0.0227 | 489 | 113 | 12782 | OBSCN, MYL4, MYL5, MYH1, SYNC, PDLIM3, MYH4, MYOZ2, MYH8, TNNI2, VCL, JUP, TNNT1, ANKRD23, TRIM32, MYH13 | 3.7011 | 2.49 E-05 | 0.0348 |
| GO:0008307~structural constituent of muscle | MF_FAT | 3 | 10 | 0.0142 | 508 | 42 | 12983 | OBSCN, MYL4, MYL5, SMTN, MYBPC2, PDLIM3, MYH4, ACTN3, MYH8, PLEC | 6.0850 | 2.77 E-05 | 0.0425 |
| GO:0005859~muscle myosin complex | CC_FAT | 4 | 7 | 0.0099 | 489 | 20 | 12782 | MYL4, MYL5, MYH1, TRIM32, MYH4, MYH13, MYH8 | 9.1487 | 7.37E-05 | 0.1030 |
| GO:0030016~myofibril | CC_FAT | 5 | 15 | 0.0212 | 489 | 111 | 12782 | OBSCN, MYL4, MYH1, SYNC, PDLIM3, MYH4, MYOZ2, MYH8, TNNI2, VCL, JUP, TNNT1, ANKRD23, TRIM32, MYH13 | 3.5323 | 8.17 E-05 | 0.1142 |
| GO:0016460~myosin II complex | CC_FAT | 6 | 7 | 0.0099 | 489 | 22 | 12782 | MYL4, MYL5, MYH1, TRIM32, MYH4, MYH13, MYH8 | 8.3170 | 0.0001 | 0.1858 |
| GO:0003012~muscle system process | BP_FAT | 7 | 18 | 0.0255 | 520 | 168 | 13528 | MYL4, MYBPC2, MYH1, MYH4, MSTN, GAL, MYH8, TNNI2, ARHGEF11, SMTN, P2RX6, TNNT1, CHRM3, RYR3, MYH13, HCN4, STBD1, DTNA | 2.7874 | 0.0003 | 0.4445 |
| GO:0006936~muscle contraction | BP_FAT | 8 | 17 | 0.0241 | 520 | 153 | 13528 | MYL4, MYBPC2, MYH1, MYH4, GAL, MYH8, TNNI2, ARHGEF11, SMTN, P2RX6, TNNT1, CHRM3, RYR3, MYH13, HCN4, STBD1, DTNA | 2.8906 | 0.0003 | 0.4611 |
| GO:0032982~myosin filament | CC_FAT | 9 | 6 | 0.0085 | 489 | 19 | 12782 | MYBPC2, MYH1, TRIM32, MYH4, MYH13, MYH8 | 8.2544 | 0.0006 | 0.8254 |
| GO:0030017~sarcomere | CC_FAT | 10 | 12 | 0.0170 | 489 | 98 | 12782 | JUP, OBSCN, MYL4, TNNT1, MYH1, ANKRD23, TRIM32, SYNC, PDLIM3, MYH4, MYOZ2, TNNI2 | 3.2007 | 0.0013 | 1.7392 |
| GO:0034330~cell junction organization | BP_FAT | 11 | 9 | 0.0127 | 520 | 57 | 13528 | SHROOM2, NF2, TAOK2, NLGN4X, NLGN2, ACTN3, VCL, TGFB2, FN1 | 4.1077 | 0.0014 | 2.4514 |
| GO:0015629~actin cytoskeleton | CC_FAT | 12 | 22 | 0.0312 | 489 | 269 | 12782 | IPP, MYL4, SHROOM2, SH3PXD2A, MYL5, MYBPC2, MYH1, CAPZA1, PDLIM3, MYH4, MYOZ2, ACTN3, MYH8, TNNI2, VCL, JUP, SMTN, TNNT1, TRIM32, MYH13, ZYX, BIN1 | 2.1378 | 0.0015 | 2.1460 |
| GO:0008201~heparin binding | MF_FAT | 13 | 12 | 0.0170 | 508 | 103 | 12983 | LPL, FGFR1, FGF7, APOE, SELL, SERPINA5, RSPO3, COMP, PF4, CCL7, LRPAP1, FN1 | 2.9775 | 0.0022 | 3.3929 |
| GO:0006793~phosphorus metabolic process | BP_FAT | 14 | 56 | 0.0793 | 520 | 973 | 13528 | PASK, AURKC, PKMYT1, CAMKK2, TGFB2, MAP3K6, SIK2, IPMK, CHUK, RET, PHKG1, PTPRN2, MYLK4, PTPRS, ALK, OSM, MTMR11, HIPK1, PDGFRA, LRRK2, ERC1, ACVR1, FGFR1, FGFR4, PPP1R2P9, ERBB2, DRD4, MAPKAPK3, FPR1, ATP5G2, PPM1B, SRC, MTMR3, GNPTAB, PTPLA, EGF, PIK3R4, GPD2, FCER1A, TCIRG1, PTPN6, GPD1, OBSCN, NDUFA3, TAOK2, PTPN18, ALPK2, MAP2K2, NADK, DUSP21, EPHA1, DUSP3, MAPK14, PGK1, DUSP8, PHPT1 | 1.4973 | 0.0024 | 4.1515 |
| GO:0006796~phosphate metabolic process | BP_FAT | 15 | 56 | 0.0793 | 520 | 973 | 13528 | PASK, AURKC, PKMYT1, CAMKK2, TGFB2, MAP3K6, SIK2, IPMK, CHUK, RET, PHKG1, PTPRN2, MYLK4, PTPRS, ALK, OSM, MTMR11, HIPK1, PDGFRA, LRRK2, ERC1, ACVR1, FGFR1, FGFR4, PPP1R2P9, ERBB2, DRD4, MAPKAPK3, FPR1, ATP5G2, PPM1B, SRC, MTMR3, GNPTAB, PTPLA, EGF, PIK3R4, GPD2, FCER1A, TCIRG1, PTPN6, GPD1, OBSCN, NDUFA3, TAOK2, PTPN18, ALPK2, MAP2K2, NADK, DUSP21, EPHA1, DUSP3, MAPK14, PGK1, DUSP8, PHPT1 | 1.4973 | 0.0024 | 4.1515 |
| GO:0046983~protein dimerization activity | MF_FAT | 16 | 36 | 0.0510 | 508 | 542 | 12983 | SLC9A7, HNF1A, MASP1, HMGCR, ERBB2, HPS4, POLA1, RNASEH1, CDSN, TGFB2, BATF, PEF1, APOE, OXCT1, JUND, ENO3, GPD1, NFE2, MSH3, NCF4, TBX1, TPD52L1, WRN, ACTN3, MAFK, FOXP2, RENBP, P2RX6, TSC2, NLGN4X, PDGFRA, TFAP2C, LRRK2, BIN1, RNF40, ACVR1 | 1.6975 | 0.0024 | 3.6423 |
| GO:0008092~cytoskeletal protein binding | MF_FAT | 17 | 34 | 0.0482 | 508 | 504 | 12983 | MYL4, SHROOM2, MYBPC2, CAPZA1, PDLIM3, KLC3, KLHL1, VCL, PACSIN1, PACSIN2, APOE, SSX2IP, LMOD2, PLEC, DYNC1I1, INF2, OBSCN, IPP, RET, GABARAPL1, NF2, MYH1, MYH4, ACTN3, MYH8, GPR98, TNNI2, JUP, TNNT1, SMTN, XIRP1, TRIM32, MYH13, TMSB4Y | 1.7241 | 0.0026 | 3.9083 |
| GO:0042803~protein homodimerization activity | MF_FAT | 18 | 25 | 0.0354 | 508 | 334 | 12983 | GPD1, SLC9A7, HNF1A, MASP1, MSH3, HMGCR, HPS4, RNASEH1, TPD52L1, TBX1, WRN, ACTN3, CDSN, FOXP2, TGFB2, RENBP, APOE, OXCT1, NLGN4X, TSC2, PDGFRA, ENO3, LRRK2, RNF40, ACVR1 | 1.9130 | 0.0030 | 4.5511 |
| GO:0007167~enzyme linked receptor protein signaling pathway | BP_FAT | 19 | 25 | 0.0354 | 520 | 342 | 13528 | FGFR1, FGFR4, FGF7, LTBP3, ERBB2, FGF17, LOC220077, SRC, TGFB2, ANGPTL1, EGF, SIK2, RET, SMAD9, MSTN, SKI, FGF22, ALK, EPHA1, ERBB2IP, TSC2, PDGFRA, TGFBRAP1, PDZD3, ACVR1 | 1.9017 | 0.0033 | 5.6639 |
| GO:0006090~pyruvate metabolic process | BP_FAT | 20 | 7 | 0.0099 | 520 | 42 | 13528 | GPD2, GPD1, LDHB, SLC16A1, SLC16A7, SLC16A8, PDHA1 | 4.3359 | 0.0050 | 8.5148 |
| GO:0045178~basal part of cell | CC_FAT | 21 | 6 | 0.0085 | 489 | 30 | 12782 | ERBB2IP, ERBB2, SLC16A8, HFE, DOCK7, FKBPL | 5.2278 | 0.0051 | 6.9505 |
| GO:0006006~glucose metabolic process | BP_FAT | 22 | 14 | 0.0198 | 520 | 153 | 13528 | PRKAG3, GPD2, LDHB, GPD1, PHKG1, SLC37A4, PGD, KCNJ11, GAPDHS, MAPK14, ENO3, PDHA1, PGK1, DCXR | 2.3805 | 0.0060 | 10.1384 |
| GO:0043395~heparan sulfate proteoglycan binding | MF_FAT | 23 | 4 | 0.0057 | 508 | 11 | 12983 | GPC4, GPC3, COMP, GPC1 | 9.2935 | 0.0077 | 11.2186 |
| GO:0006941~striated muscle contraction | BP_FAT | 24 | 7 | 0.0099 | 520 | 46 | 13528 | MYL4, TNNT1, MYH1, RYR3, TNNI2, DTNA, ARHGEF11 | 3.9589 | 0.0079 | 13.0584 |
| GO:0005539~glycosaminoglycan binding | MF_FAT | 25 | 13 | 0.0184 | 508 | 140 | 12983 | LPL, FGFR1, FGF7, SELL, PGLYRP1, PF4, CCL7, LRPAP1, APOE, COMP, RSPO3, SERPINA5, FN1 | 2.3732 | 0.0087 | 12.5479 |
| GO:0005833~hemoglobin complex | CC_FAT | 26 | 4 | 0.0057 | 489 | 12 | 12782 | HBM, HBG1, HBB, HBD | 8.7130 | 0.0094 | 12.3808 |
| GO:0014823~response to activity | BP_FAT | 27 | 4 | 0.0057 | 520 | 12 | 13528 | OXT, MYH4, MSTN, MYH8 | 8.6718 | 0.0095 | 15.5787 |
| GO:0005913~cell-cell adherens junction | CC_FAT | 28 | 6 | 0.0085 | 489 | 35 | 12782 | JUP, SHROOM2, XIRP1, ZYX, SSX2IP, VCL | 4.4810 | 0.0100 | 13.1306 |
| GO:0045216~cell-cell junction organization | BP_FAT | 29 | 6 | 0.0085 | 520 | 35 | 13528 | SHROOM2, NF2, NLGN4X, NLGN2, VCL, TGFB2 | 4.4598 | 0.0102 | 16.6062 |
| GO:0016459~myosin complex | CC_FAT | 30 | 8 | 0.0113 | 489 | 65 | 12782 | MYL4, MYL5, MYBPC2, MYH1, TRIM32, MYH4, MYH13, MYH8 | 3.2171 | 0.0117 | 15.1332 |
| GO:0015671~oxygen transport | BP_FAT | 31 | 4 | 0.0057 | 520 | 13 | 13528 | HBM, HBG1, HBB, HBD | 8.0047 | 0.0120 | 19.2844 |
| GO:0022610~biological adhesion | BP_FAT | 32 | 40 | 0.0567 | 520 | 701 | 13528 | MYBPC2, PCDHB13, PCDHB12, CDSN, SRC, SCARF2, VCL, CLDN15, LAMB3, COMP, RHOB, SSX2IP, ZYX, FN1, COL18A1, ICAM1, RET, TAOK2, LPP, PCDHB6, SELL, LRRN2, PTPRS, NLGN2, ACTN3, SLAMF7, SSPO, PRPH2, RAPH1, GPR98, NCAM1, JUP, ERBB2IP, PKP3, NLGN4X, ITGA7, FCGBP, ADAM22, MFAP4, MUC5B | 1.4845 | 0.0124 | 19.8241 |
| GO:0043394~proteoglycan binding | MF_FAT | 33 | 4 | 0.0057 | 508 | 13 | 12983 | GPC4, GPC3, COMP, GPC1 | 7.8637 | 0.0126 | 17.7245 |
| GO:0005344~oxygen transporter activity | MF_FAT | 34 | 4 | 0.0057 | 508 | 13 | 12983 | HBM, HBG1, HBB, HBD | 7.8637 | 0.0126 | 17.7245 |
| GO:0007169~transmembrane receptor protein tyrosine kinase signaling pathway | BP_FAT | 35 | 17 | 0.0241 | 520 | 224 | 13528 | FGFR1, FGFR4, RET, FGF7, ERBB2, FGF17, FGF22, LOC220077, ALK, EPHA1, SRC, ERBB2IP, TSC2, PDGFRA, ANGPTL1, EGF, SIK2 | 1.9744 | 0.0127 | 20.1749 |
| GO:0007155~cell adhesion | BP_FAT | 36 | 40 | 0.0567 | 520 | 700 | 13528 | MYBPC2, PCDHB13, PCDHB12, CDSN, SRC, SCARF2, VCL, CLDN15, LAMB3, COMP, RHOB, SSX2IP, ZYX, FN1, COL18A1, ICAM1, RET, TAOK2, LPP, PCDHB6, SELL, LRRN2, PTPRS, NLGN2, ACTN3, SLAMF7, SSPO, PRPH2, RAPH1, GPR98, NCAM1, JUP, ERBB2IP, PKP3, NLGN4X, ITGA7, FCGBP, ADAM22, MFAP4, MUC5B | 1.4866 | 0.0127 | 20.1800 |
| GO:0005615~extracellular space | CC_FAT | 37 | 39 | 0.0552 | 489 | 685 | 12782 | FGF7, MASP1, FGF17, OXT, PGC, LEPR, IFNW1, PF4, CCL7, TGFB2, GPC4, IL17D, GPC3, APOE, DMKN, ANGPTL1, LECT2, EGF, GPC1, FKBPL, FN1, COL18A1, ICAM1, LPL, IL6, PSAP, CST3, MSTN, FGF22, SSPO, OSM, NUCB1, SERPINB5, DEFA4, KCP, TFPI, DEFA1, CMTM3, CMTM6 | 1.4882 | 0.0133 | 17.1307 |
| GO:0007610~behavior | BP_FAT | 38 | 29 | 0.0411 | 520 | 469 | 13528 | HCRT, HMGCR, LEPR, OXT, DRD4, PGLYRP1, FPR1, PF4, CCL7, TGFB2, KLHL1, HCRTR2, ATP2B2, PPP1R1B, MRGPRX2, LECT2, IL6, TBX1, GAL, APRT, FOXP2, OSM, NRAS, MAPK14, TSC2, NLGN4X, DEFA1, CMTM3, CMTM6 | 1.6086 | 0.0138 | 21.7975 |
| GO:0015355~secondary active monocarboxylate transmembrane transporter activity | MF_FAT | 39 | 3 | 0.0042 | 508 | 5 | 12983 | SLC16A1, SLC16A7, SLC16A8 | 15.3343 | 0.0141 | 19.5403 |
| GO:0070161~anchoring junction | CC_FAT | 40 | 14 | 0.0198 | 489 | 172 | 12782 | SHROOM2, MYH1, NF2, LPP, TMEM53, ACTN3, CDSN, VCL, JUP, XIRP1, PKP3, SH3KBP1, SSX2IP, ZYX | 2.1276 | 0.0147 | 18.6880 |
| GO:0044421~extracellular region part | CC_FAT | 41 | 51 | 0.0722 | 489 | 960 | 12782 | FGF7, MASP1, PGC, LEPR, FGF17, MMP28, TGFB2, GPC4, WNT4, GPC3, APOE, DMKN, GPC1, COCH, ICAM1, CST3, FGF22, SSPO, NUCB1, OSM, SERPINB5, KCP, DEFA4, TFPI, DEFA1, MFAP4, ADAMTS19, OXT, IFNW1, PF4, CCL7, IL17D, LAMB3, COL9A3, COMP, ANGPTL1, LECT2, EGF, FKBPL, FN1, COL18A1, LPL, IL6, PSAP, NTN4, MSTN, SPARC, ERBB2IP, PI3, CMTM3, CMTM6 | 1.3886 | 0.0148 | 18.7926 |
| GO:0016311~dephosphorylation | BP_FAT | 42 | 13 | 0.0184 | 520 | 154 | 13528 | PTPN6, PTPN18, PPP1R2P9, PTPRN2, PTPRS, DUSP21, PPM1B, MTMR11, MTMR3, DUSP3, PTPLA, DUSP8, PHPT1 | 2.1961 | 0.0155 | 24.1710 |
| GO:0005509~calcium ion binding | MF_FAT | 43 | 50 | 0.0708 | 508 | 919 | 12983 | ARSE, S100A8, MASP1, LTBP3, ANO1, MAN1B1, MYL10, MMP28, CAMKK2, FAHD1, ATP2B2, SLC24A3, PVALB, SPATA21, CIB2, PLCB2, RET, PCDHB6, CACNG4, ACTN3, SSPO, GPR98, LRPAP1, NUCB1, RYR3, MFAP4, PROS1, GALNT2, MYL4, MYL5, PPP3R1, C1R, PCDHB13, PCDHB12, PEF1, GNPTAB, CRB2, COMP, CALML6, ENTPD3, EGF, DTNA, GPD2, CPNE6, SPARC, ITPR3, DAGLB, PLCG1, EFHB, ITGA7 | 1.3905 | 0.0157 | 21.6111 |
| GO:0019318~hexose metabolic process | BP_FAT | 44 | 15 | 0.0212 | 520 | 192 | 13528 | PRKAG3, GPD2, LDHB, GPD1, PHKG1, SLC37A4, PGD, KCNJ11, GAPDHS, RENBP, MAPK14, ENO3, PDHA1, PGK1, DCXR | 2.0325 | 0.0161 | 24.9809 |
| GO:0001871~pattern binding | MF_FAT | 45 | 13 | 0.0184 | 508 | 154 | 12983 | LPL, FGFR1, FGF7, SELL, PGLYRP1, PF4, CCL7, LRPAP1, APOE, COMP, RSPO3, SERPINA5, FN1 | 2.1574 | 0.0176 | 23.8220 |
| GO:0030247~polysaccharide binding | MF_FAT | 46 | 13 | 0.0184 | 508 | 154 | 12983 | LPL, FGFR1, FGF7, SELL, PGLYRP1, PF4, CCL7, LRPAP1, APOE, COMP, RSPO3, SERPINA5, FN1 | 2.1574 | 0.0176 | 23.8220 |
| GO:0031093~platelet alpha granule lumen | CC_FAT | 47 | 6 | 0.0085 | 489 | 41 | 12782 | PF4, SPARC, EGF, PROS1, TGFB2, FN1 | 3.8252 | 0.0192 | 23.7441 |
| GO:0007265~Ras protein signal transduction | BP_FAT | 48 | 10 | 0.0142 | 520 | 105 | 13528 | NRAS, ARHGDIG, MAP2K2, APOE, MAPK14, MAPKAPK3, RHOB, LOC220077, SRC, ARHGEF11 | 2.4777 | 0.0195 | 29.3426 |
| GO:0006811~ion transport | BP_FAT | 49 | 42 | 0.0595 | 520 | 768 | 13528 | SLC9A9, SLC9A7, KCNK15, SLC5A4, GLRA2, ANO1, HFE, ATP5G2, KCNK13, KCNJ11, CNGB3, SHKBP1, ATP2B2, KCNQ5, SLC16A1, SLC24A3, SLC25A22, HCN4, SLC4A3, SLC1A1, KCNG2, TCIRG1, CNNM3, CLCC1, ATP4B, SLC12A3, ATP11B, CACNG4, ITPR3, KCTD7, KCNK4, TST, ABCC9, P2RX6, ATP13A1, SLC16A7, RYR3, SLC16A8, SLC41A1, SLC13A4, SLC38A1, PDZD3 | 1.4227 | 0.0198 | 29.7881 |
| GO:0006813~potassium ion transport | BP_FAT | 50 | 13 | 0.0184 | 520 | 160 | 13528 | KCNK15, SLC9A7, ATP4B, KCNK13, KCNJ11, KCTD7, KCNK4, SHKBP1, KCNQ5, ABCC9, SLC24A3, HCN4, KCNG2 | 2.1138 | 0.0204 | 30.5068 |
| GO:0043086~negative regulation of catalytic activity | BP_FAT | 51 | 19 | 0.0269 | 520 | 277 | 13528 | NF2, HMGCR, OPRL1, LEPR, PIF1, DRD4, PKIG, CST3, INTS1, TP73, TGFB2, CDKN1C, PSMB7, GMIP, APOE, PSMA3, TSC2, ADCY10, DUSP8 | 1.7844 | 0.0205 | 30.6737 |
| GO:0005887~integral to plasma membrane | CC_FAT | 52 | 60 | 0.0850 | 489 | 1188 | 12782 | GYPB, IL27RA, GLRA2, AQP4, SLC7A9, CD52, KCNJ11, LGR5, ART1, SHKBP1, GPC4, KCNQ5, GPC3, ZYX, HTR1D, SLC4A3, SLC1A1, GPC1, KCNG2, ICAM1, PCDHB6, PTPRN2, NCF4, PTPRS, CACNG4, ALK, OSM, CHRM3, SSTR1, PDGFRA, STBD1, ACVR1, FGFR1, FGFR4, DRD4, HFE, PCDHB12, GPR68, TPSG1, HCRTR2, SLC29A1, MC3R, FCER1A, TCIRG1, IL6, OPRL1, SELL, SLC12A3, PHB, ITPR3, EPHA1, CYB561, KCTD7, ABCC9, P2RX6, SLC16A7, NLGN4X, SLC16A8, ITGA7, SLC13A4 | 1.3202 | 0.0209 | 25.5558 |
| GO:0048729~tissue morphogenesis | BP_FAT | 53 | 14 | 0.0198 | 520 | 180 | 13528 | RET, FGF7, HNF1A, NF2, TGFB2, WNT4, GPC3, SIX1, TSC2, IFT52, IPMK, CHUK, ACVR1, SMARCA4 | 2.0234 | 0.0214 | 31.8062 |
| GO:0032403~protein complex binding | MF_FAT | 54 | 15 | 0.0212 | 508 | 196 | 12983 | FCER1A, ICAM1, ADAM11, BCKDHB, WRN, ACTN3, LOC220077, LAMB3, ERBB2IP, FCGR2C, ADAM22, FCGR3A, GRB14, RNF40, ACVR1 | 1.9559 | 0.0217 | 28.5369 |
| GO:0006820~anion transport | BP_FAT | 55 | 12 | 0.0170 | 520 | 143 | 13528 | TST, SLC16A1, SLC16A7, CLCC1, SLC12A3, SLC16A8, SLC25A22, ANO1, GLRA2, SLC13A4, SLC4A3, SLC1A1 | 2.1831 | 0.0218 | 32.2635 |
| GO:0031226~intrinsic to plasma membrane | CC_FAT | 56 | 61 | 0.0864 | 489 | 1215 | 12782 | GYPB, IL27RA, GLRA2, AQP4, SLC7A9, CD52, KCNJ11, LGR5, ART1, SHKBP1, GPC4, KCNQ5, GPC3, ZYX, HTR1D, SLC4A3, SLC1A1, GPC1, KCNG2, ICAM1, PCDHB6, PTPRN2, NCF4, PTPRS, CACNG4, ALK, OSM, CHRM3, SSTR1, PDGFRA, STBD1, ACVR1, FGFR1, FGFR4, DRD4, HFE, PCDHB12, GPR68, TPSG1, HCRTR2, SLC29A1, MC3R, FCER1A, TCIRG1, IL6, OPRL1, SELL, SLC12A3, PHB, GAS1, ITPR3, EPHA1, CYB561, KCTD7, ABCC9, P2RX6, SLC16A7, NLGN4X, SLC16A8, ITGA7, SLC13A4 | 1.3123 | 0.0224 | 27.1961 |
| GO:0005217~intracellular ligand-gated ion channel activity | MF_FAT | 57 | 4 | 0.0057 | 508 | 16 | 12983 | RYR3, HCN4, ITPR3, CNGB3 | 6.3893 | 0.0227 | 29.6778 |
| GO:0005856~cytoskeleton | CC_FAT | 58 | 68 | 0.0963 | 489 | 1381 | 12782 | MAD1L1, MYBPC2, TTLL9, FAM110B, CAPZA1, PDLIM3, AURKC, CDSN, KRT33A, KLHL1, VCL, KIF2C, FRMD6, ZYX, ADCY10, LMOD2, DYNC1I1, SH3PXD2A, MYH1, MYH4, ACTN3, MYH8, JUP, TNNT1, SMTN, HIPK1, KRT15, PSMA3, TRIM32, SH3KBP1, TMSB4Y, MAP7D1, BIN1, IFT88, KIF26B, SDCCAG8, SHROOM2, MYL4, KRT6B, MYL5, LMNB1, SYNC, KLC3, KRTAP10-12, C10ORF113, NDRG2, ELMOD2, PLEC, IPP, GABARAPL1, NFE2, NF2, MYOZ2, WRN, SHANK1, KIF3C, RAPH1, ACTL9, TNNI2, RASSF3, P2RX6, SEPT7P2, MAPK14, MCPH1, KRT78, TSC2, MAP4, MYH13 | 1.2871 | 0.0228 | 27.5670 |
| GO:0008543~fibroblast growth factor receptor signaling pathway | BP_FAT | 59 | 5 | 0.0071 | 520 | 29 | 13528 | FGFR1, FGFR4, FGF7, FGF17, FGF22 | 4.4854 | 0.0238 | 34.6473 |
| GO:0043408~regulation of MAPKKK cascade | BP_FAT | 60 | 10 | 0.0142 | 520 | 109 | 13528 | OSM, FCER1A, MAP3K6, IL6, NF2, TAOK2, ERBB2, LEPR, TP73, TGFB2 | 2.3867 | 0.0242 | 35.1182 |
| GO:0060205~cytoplasmic membrane-bounded vesicle lumen | CC_FAT | 61 | 6 | 0.0085 | 489 | 44 | 12782 | PF4, SPARC, EGF, PROS1, TGFB2, FN1 | 3.5644 | 0.0254 | 30.1809 |
| GO:0004721~phosphoprotein phosphatase activity | MF_FAT | 62 | 13 | 0.0184 | 508 | 165 | 12983 | PTPN6, MTMR3, DUSP3, PTPN18, PTPRN2, PTPLA, PPP3R1, PTPRS, DUSP21, PPM1B, DUSP8, PHPT1, SSU72 | 2.0136 | 0.0284 | 35.6829 |
| GO:0010647~positive regulation of cell communication | BP_FAT | 63 | 21 | 0.0297 | 520 | 329 | 13528 | FCER1A, HCRT, IL6, ING2, TAOK2, LEPR, ERBB2, OXT, GAS1, LGALS9, SRC, TGFB2, CDKN1C, NCAM1, OSM, NRAS, GPC3, ZDHHC13, KCP, EEF1D, EGF | 1.6606 | 0.0288 | 40.3203 |
| GO:0050808~synapse organization | BP_FAT | 64 | 7 | 0.0099 | 520 | 61 | 13528 | ATP2B2, PCDHB6, ERBB2, GLRA2, NLGN4X, NLGN2, PCDHB13 | 2.9854 | 0.0290 | 40.5367 |
| GO:0005626~insoluble fraction | CC_FAT | 65 | 44 | 0.0623 | 489 | 839 | 12782 | ARHGDIG, FGFR1, HMGCR, HPS4, MAN1B1, PKMYT1, CD52, KCNJ11, SLC29A1, ATP2B2, MTMR3, TSPAN12, SLC16A1, RHOB, ENO3, SLC4A3, SLC1A1, PLEC, DTNA, RAB8B, ACY3, SLC12A3, CLCC1, CPNE6, ITPR3, SHANK1, JUP, NRAS, ABCC9, P2RX6, SLC16A7, SSTR1, SLC16A8, TSC2, SH3KBP1, AMFR, SLC38A1, TAPBPL, STBD1, LRRK2, PDZD3, RAPGEFL1, RNF40, GRB14 | 1.3708 | 0.0296 | 34.2823 |
| GO:0015669~gas transport | BP_FAT | 66 | 4 | 0.0057 | 520 | 18 | 13528 | HBM, HBG1, HBB, HBD | 5.7812 | 0.0298 | 41.4529 |
| GO:0031983~vesicle lumen | CC_FAT | 67 | 6 | 0.0085 | 489 | 46 | 12782 | PF4, SPARC, EGF, PROS1, TGFB2, FN1 | 3.4094 | 0.0301 | 34.7838 |
| GO:0051348~negative regulation of transferase activity | BP_FAT | 68 | 9 | 0.0127 | 520 | 96 | 13528 | CDKN1C, NF2, HMGCR, APOE, PIF1, PKIG, TSC2, DUSP8, TP73 | 2.4389 | 0.0310 | 42.6984 |
| GO:0006470~protein amino acid dephosphorylation | BP_FAT | 69 | 11 | 0.0156 | 520 | 133 | 13528 | PTPN6, MTMR3, DUSP3, PTPN18, PPP1R2P9, PTPRN2, PTPLA, PTPRS, DUSP21, PPM1B, DUSP8 | 2.1516 | 0.0320 | 43.7594 |
| GO:0002252~immune effector process | BP_FAT | 70 | 11 | 0.0156 | 520 | 134 | 13528 | ICAM1, GALNT2, ABCC9, IL6, MASP1, MSH3, STXBP2, C4BPB, C1R, SLAMF7, CTNNBL1 | 2.1356 | 0.0335 | 45.2296 |
| GO:0007224~smoothened signaling pathway | BP_FAT | 71 | 4 | 0.0057 | 520 | 19 | 13528 | HIPK1, DISP1, IFT52, IFT88 | 5.4769 | 0.0344 | 46.1805 |
| GO:0046626~regulation of insulin receptor signaling pathway | BP_FAT | 72 | 4 | 0.0057 | 520 | 19 | 13528 | TSC2, SIK2, GRB14, SRC | 5.4769 | 0.0344 | 46.1805 |
| GO:0035176~social behavior | BP_FAT | 73 | 4 | 0.0057 | 520 | 19 | 13528 | OXT, DRD4, NLGN4X, TBX1 | 5.4769 | 0.0344 | 46.1805 |
| GO:0051051~negative regulation of transport | BP_FAT | 74 | 11 | 0.0156 | 520 | 135 | 13528 | OSM, HCRT, PACSIN1, NFKBIE, PACSIN2, OXT, PKIG, DRD4, EGF, TACC3, KCNJ11 | 2.1198 | 0.0350 | 46.7077 |
| GO:0005912~adherens junction | CC_FAT | 75 | 12 | 0.0170 | 489 | 155 | 12782 | JUP, SHROOM2, MYH1, NF2, XIRP1, LPP, TMEM53, SH3KBP1, ZYX, SSX2IP, ACTN3, VCL | 2.0237 | 0.0353 | 39.5309 |
| GO:0009967~positive regulation of signal transduction | BP_FAT | 76 | 19 | 0.0269 | 520 | 295 | 13528 | FCER1A, IL6, ING2, TAOK2, LEPR, ERBB2, GAS1, LGALS9, SRC, TGFB2, CDKN1C, NCAM1, OSM, NRAS, GPC3, ZDHHC13, KCP, EEF1D, EGF | 1.6756 | 0.0358 | 47.5336 |
| GO:0043410~positive regulation of MAPKKK cascade | BP_FAT | 77 | 6 | 0.0085 | 520 | 48 | 13528 | OSM, IL6, TAOK2, ERBB2, LEPR, TGFB2 | 3.2519 | 0.0360 | 47.7142 |
| GO:0031047~gene silencing by RNA | BP_FAT | 78 | 5 | 0.0071 | 520 | 33 | 13528 | MOV10, RBM3, CELF1, PIWIL4, TNRC6A | 3.9417 | 0.0364 | 48.0414 |
| GO:0005198~structural molecule activity | MF_FAT | 79 | 35 | 0.0496 | 508 | 634 | 12983 | MYL4, KRT6B, MYL5, LMNB1, MYBPC2, SYNC, PDLIM3, KRT33A, VCL, CLDN15, LAMB3, WNT4, COL9A3, COMP, RPL39L, PLEC, CCDC108, FN1, COL18A1, OBSCN, RPL35A, MOBP, MRPS22, MYH4, ACTN3, MYH8, JUP, SMTN, ISCA2, ERBB2IP, KRT15, KRT78, MAP4, MUC5B, COPE | 1.4109 | 0.0377 | 44.5665 |
| GO:0015672~monovalent inorganic cation transport | BP_FAT | 80 | 20 | 0.0283 | 520 | 318 | 13528 | TCIRG1, SLC9A9, SLC9A7, KCNK15, SLC5A4, SLC12A3, ATP4B, ATP5G2, KCNK13, KCNJ11, KCTD7, KCNK4, SHKBP1, KCNQ5, ABCC9, SLC24A3, SLC38A1, SLC13A4, HCN4, KCNG2 | 1.6362 | 0.0379 | 49.5031 |
| GO:0005624~membrane fraction | CC_FAT | 81 | 42 | 0.0595 | 489 | 809 | 12782 | ARHGDIG, FGFR1, HMGCR, HPS4, MAN1B1, PKMYT1, CD52, KCNJ11, SLC29A1, ATP2B2, MTMR3, TSPAN12, SLC16A1, RHOB, ENO3, SLC4A3, SLC1A1, DTNA, SLC12A3, CLCC1, ACY3, CPNE6, ITPR3, SHANK1, JUP, NRAS, ABCC9, P2RX6, SLC16A7, SSTR1, TSC2, SLC16A8, SH3KBP1, AMFR, SLC38A1, TAPBPL, STBD1, LRRK2, PDZD3, RAPGEFL1, RNF40, GRB14 | 1.3570 | 0.0381 | 41.9380 |
| GO:0015293~symporter activity | MF_FAT | 82 | 11 | 0.0156 | 508 | 137 | 12983 | SLC16A1, SLC12A9, SLC5A4, SLC16A7, SLC24A3, SLC12A3, SLC16A8, SLC25A22, SLC13A4, SLC38A1, SLC1A1 | 2.0520 | 0.0421 | 48.3165 |
| GO:0060562~epithelial tube morphogenesis | BP_FAT | 83 | 7 | 0.0099 | 520 | 67 | 13528 | WNT4, RET, GPC3, SIX1, TSC2, IFT52, IPMK | 2.7180 | 0.0431 | 54.1029 |
| GO:0006928~cell motion | BP_FAT | 84 | 27 | 0.0382 | 520 | 475 | 13528 | ICAM1, SHROOM2, RET, IL6, SOX1, TAOK2, HMGCR, ERBB2, CAPZA1, FPR1, PF4, TBX1, GAS1, SRC, TGFB2, VCL, ARHGEF11, GAPDHS, SERPINB5, ETS1, MAPK14, SIX1, CELF3, AMFR, ETV4, ACVR1, FN1 | 1.4788 | 0.0437 | 54.6444 |
| GO:0051705~behavioral interaction between organisms | BP_FAT | 85 | 5 | 0.0071 | 520 | 35 | 13528 | PPP1R1B, OXT, DRD4, NLGN4X, TBX1 | 3.7165 | 0.0438 | 54.7324 |
| GO:0045859~regulation of protein kinase activity | BP_FAT | 86 | 21 | 0.0297 | 520 | 345 | 13528 | FCER1A, HCRT, NF2, TAOK2, HMGCR, ERBB2, DRD4, PKIG, FPR1, PKMYT1, TP73, CAMKK2, TGFB2, CDKN1C, MNAT1, MAP3K6, APOE, TSC2, ADCY10, EGF, DUSP8 | 1.5835 | 0.0444 | 55.2168 |
| GO:0002673~regulation of acute inflammatory response | BP_FAT | 87 | 4 | 0.0057 | 520 | 21 | 13528 | OSM, FCER1A, IL6, MASP1 | 4.9553 | 0.0447 | 55.4347 |
| GO:0035194~posttranscriptional gene silencing by RNA | BP_FAT | 88 | 4 | 0.0057 | 520 | 21 | 13528 | MOV10, RBM3, CELF1, TNRC6A | 4.9553 | 0.0447 | 55.4347 |
| GO:0016441~posttranscriptional gene silencing | BP_FAT | 89 | 4 | 0.0057 | 520 | 21 | 13528 | MOV10, RBM3, CELF1, TNRC6A | 4.9553 | 0.0447 | 55.4347 |
| GO:0043405~regulation of MAP kinase activity | BP_FAT | 90 | 11 | 0.0156 | 520 | 141 | 13528 | FCER1A, MAP3K6, HMGCR, APOE, ERBB2, DRD4, TSC2, FPR1, EGF, DUSP8, TP73 | 2.0296 | 0.0449 | 55.6302 |
| GO:0004714~transmembrane receptor protein tyrosine kinase activity | MF_FAT | 91 | 7 | 0.0099 | 508 | 67 | 12983 | FGFR1, RET, FGFR4, ERBB2, PDGFRA, ALK, EPHA1 | 2.6701 | 0.0463 | 51.6610 |
| GO:0005996~monosaccharide metabolic process | BP_FAT | 92 | 15 | 0.0212 | 520 | 222 | 13528 | PRKAG3, GPD2, LDHB, GPD1, PHKG1, SLC37A4, PGD, KCNJ11, GAPDHS, RENBP, MAPK14, ENO3, PDHA1, PGK1, DCXR | 1.7578 | 0.0471 | 57.4062 |
| GO:0007243~protein kinase cascade | BP_FAT | 93 | 22 | 0.0312 | 520 | 370 | 13528 | FCER1A, PRKAG3, FGFR1, RET, TAOK2, ERBB2, DRD4, FPR1, LOC220077, SRC, CAMKK2, OSM, MAP3K6, MAPK14, TSC2, LRRK2, EGF, ERC1, DUSP8, SIK2, CHUK, FKBPL | 1.5469 | 0.0478 | 57.9112 |
| GO:0043407~negative regulation of MAP kinase activity | BP_FAT | 94 | 5 | 0.0071 | 520 | 36 | 13528 | HMGCR, APOE, TSC2, DUSP8, TP73 | 3.6132 | 0.0479 | 57.9991 |
| GO:0000267~cell fraction | CC_FAT | 95 | 53 | 0.0751 | 489 | 1083 | 12782 | LDHB, HMGCR, RAB3GAP1, HPS4, MAN1B1, PKMYT1, CD52, KCNJ11, ATP2B2, TSPAN12, SLC16A1, RHOB, ADCY10, SLC1A1, SLC4A3, PKIG, JUP, SSTR1, RARS, SH3KBP1, AMFR, SLC38A1, STBD1, LRRK2, RAPGEFL1, GRB14, ARHGDIG, FGFR1, PPP4R1, SLC29A1, MTMR3, ENO3, EGF, DTNA, PLEC, GPD1, RAB8B, MOBP, ACY3, CLCC1, SLC12A3, CPNE6, SHANK1, ITPR3, NRAS, ABCC9, P2RX6, SLC16A7, SLC16A8, TSC2, TAPBPL, PDZD3, RNF40 | 1.2792 | 0.0490 | 50.4630 |
| GO:0043062~extracellular structure organization | BP_FAT | 96 | 12 | 0.0170 | 520 | 163 | 13528 | COL18A1, ATP2B2, PCDHB6, ERBB2, GLRA2, NLGN4X, PDGFRA, CST3, NLGN2, PCDHB13, SMARCA4, TGFB2 | 1.9152 | 0.0494 | 59.1670 |
| GO:0051338~regulation of transferase activity | BP_FAT | 97 | 22 | 0.0312 | 520 | 372 | 13528 | FCER1A, HCRT, NF2, TAOK2, HMGCR, ERBB2, PIF1, DRD4, PKIG, FPR1, PKMYT1, TP73, CAMKK2, TGFB2, CDKN1C, MNAT1, MAP3K6, APOE, TSC2, ADCY10, EGF, DUSP8 | 1.5385 | 0.0496 | 59.3037 |
| GO:0004725~protein tyrosine phosphatase activity | MF_FAT | 98 | 9 | 0.0127 | 508 | 104 | 12983 | PTPN6, MTMR3, DUSP3, PTPN18, PTPRN2, PTPLA, PTPRS, DUSP21, DUSP8 | 2.2117 | 0.0505 | 54.8621 |
| GO:0003774~motor activity | MF_FAT | 99 | 11 | 0.0156 | 508 | 142 | 12983 | DYNC1I1, KIF2C, MYL4, MYL5, MYH1, MYH4, MYH13, KLC3, MYH8, KIF3C, KIF26B | 1.9798 | 0.0515 | 55.5860 |
| GO:0016310~phosphorylation | BP_FAT | 100 | 41 | 0.0581 | 520 | 800 | 13528 | FGFR1, FGFR4, ERBB2, DRD4, PASK, FPR1, MAPKAPK3, AURKC, PKMYT1, ATP5G2, SRC, CAMKK2, TGFB2, MAP3K6, GNPTAB, EGF, SIK2, IPMK, PIK3R4, CHUK, FCER1A, TCIRG1, OBSCN, RET, NDUFA3, TAOK2, ALPK2, MAP2K2, PHKG1, MYLK4, NADK, ALK, EPHA1, OSM, HIPK1, MAPK14, PDGFRA, PGK1, ERC1, LRRK2, ACVR1 | 1.3333 | 0.0521 | 61.2031 |
| GO:0044459~plasma membrane part | CC_FAT | 101 | 99 | 0.1402 | 489 | 2203 | 12782 | AQP4, SLC7A9, EIF5A, CD52, VCL, SHKBP1, ATP2B2, APOE, HTR1D, SLC4A3, MYH1, PTPRN2, NCF4, PTPRS, ACTN3, NCAM1, JUP, SSTR1, PDGFRA, FGFR1, FGFR4, ERBB2, DRD4, HFE, SRC, HCRTR2, SLC29A1, SSX2IP, FN1, FCER1A, RAB8B, LPP, ACY3, PHB, GAS1, SHANK1, EPHA1, CYB561, KCTD7, P2RX6, PKP3, NLGN4X, TSC2, ITGA7, SLC13A4, PDZD3, GYPB, IL27RA, GLRA2, KCNJ11, LGR5, CDSN, ART1, GPC4, KCNQ5, FRMD6, GPC3, ZYX, ADCY10, SLC1A1, GPC1, KCNG2, ICAM1, PCDHB6, CACNG4, ALK, OSM, XIRP1, CHRM3, SH3KBP1, SERP2, STBD1, COPE, FAIM2, ACVR1, HCRT, SNX18, SHROOM2, PCDHB12, GPR68, CLDN15, TPSG1, MC3R, FKBPL, DTNA, TCIRG1, IL6, NF2, SLC12A3, SELL, OPRL1, TMEM53, ITPR3, RAPH1, NRAS, ABCC9, SLC16A7, ERBB2IP, SLC16A8 | 1.1747 | 0.0529 | 53.2245 |
| GO:0016051~carbohydrate biosynthetic process | BP_FAT | 102 | 9 | 0.0127 | 520 | 107 | 13528 | GPD2, B3GAT2, PRKAG3, GPD1, B3GALT6, PHKG1, MPDU1, PGD, CHST14 | 2.1882 | 0.0533 | 62.0241 |
| GO:0005916~fascia adherens | CC_FAT | 103 | 3 | 0.0042 | 489 | 10 | 12782 | JUP, XIRP1, VCL | 7.8417 | 0.0534 | 53.6119 |
| GO:0033138~positive regulation of peptidyl-serine phosphorylation | BP_FAT | 104 | 3 | 0.0042 | 520 | 10 | 13528 | OSM, IL6, DOCK7 | 7.8046 | 0.0539 | 62.4570 |
| GO:0050678~regulation of epithelial cell proliferation | BP_FAT | 105 | 7 | 0.0099 | 520 | 71 | 13528 | CDKN1C, FGF7, ERBB2, TSC2, GAS1, FOXP2, TGFB2 | 2.5649 | 0.0545 | 62.9034 |
| GO:0006163~purine nucleotide metabolic process | BP_FAT | 106 | 13 | 0.0184 | 520 | 186 | 13528 | TCIRG1, ATP4B, ATP11B, NADK, ATP5G2, AMPD3, MTHFD1, ATP2B2, ATP13A1, ATP9B, GUCA2B, ADCY10, GUK1 | 1.8183 | 0.0548 | 63.0801 |
| GO:0001948~glycoprotein binding | MF_FAT | 107 | 5 | 0.0071 | 508 | 37 | 12983 | GPC4, GPC3, ERBB2, COMP, GPC1 | 3.4537 | 0.0550 | 57.9949 |
| GO:0055037~recycling endosome | CC_FAT | 108 | 4 | 0.0057 | 489 | 23 | 12782 | SLC9A9, SLC9A7, RABEP1, HFE | 4.5459 | 0.0556 | 55.0735 |
| GO:0044092~negative regulation of molecular function | BP_FAT | 109 | 20 | 0.0283 | 520 | 334 | 13528 | NF2, HMGCR, OPRL1, LEPR, PIF1, DRD4, PKIG, CST3, INTS1, TP73, TGFB2, LRPAP1, CDKN1C, PSMB7, GMIP, APOE, PSMA3, TSC2, ADCY10, DUSP8 | 1.5578 | 0.0566 | 64.2981 |
| GO:0044430~cytoskeletal part | CC_FAT | 110 | 47 | 0.0666 | 489 | 952 | 12782 | MAD1L1, SDCCAG8, SHROOM2, MYL4, KRT6B, MYL5, LMNB1, MYBPC2, TTLL9, CAPZA1, FAM110B, SYNC, AURKC, KLC3, KRTAP10-12, C10ORF113, KRT33A, KIF2C, NDRG2, ZYX, DYNC1I1, GABARAPL1, SH3PXD2A, MYH1, MYH4, WRN, ACTN3, SHANK1, KIF3C, MYH8, TNNI2, RASSF3, TNNT1, P2RX6, HIPK1, SEPT7P2, MAPK14, KRT15, TRIM32, KRT78, TSC2, MCPH1, MAP4, MYH13, MAP7D1, IFT88, KIF26B | 1.2905 | 0.0573 | 56.2260 |
| GO:0033673~negative regulation of kinase activity | BP_FAT | 111 | 8 | 0.0113 | 520 | 90 | 13528 | CDKN1C, NF2, HMGCR, APOE, PKIG, TSC2, DUSP8, TP73 | 2.3125 | 0.0574 | 64.8269 |
| GO:0031674~I band | CC_FAT | 112 | 6 | 0.0085 | 489 | 55 | 12782 | JUP, OBSCN, ANKRD23, SYNC, PDLIM3, MYOZ2 | 2.8515 | 0.0580 | 56.6671 |
| GO:0016052~carbohydrate catabolic process | BP_FAT | 113 | 9 | 0.0127 | 520 | 109 | 13528 | GAPDHS, GPD1, LDHB, LYG2, PGD, PGLYRP1, ENO3, PDHA1, PGK1 | 2.1481 | 0.0582 | 65.3717 |
| GO:0043549~regulation of kinase activity | BP_FAT | 114 | 21 | 0.0297 | 520 | 357 | 13528 | FCER1A, HCRT, NF2, TAOK2, HMGCR, ERBB2, DRD4, PKIG, FPR1, PKMYT1, TP73, CAMKK2, TGFB2, CDKN1C, MNAT1, MAP3K6, APOE, TSC2, ADCY10, EGF, DUSP8 | 1.5303 | 0.0585 | 65.5827 |
| GO:0007423~sensory organ development | BP_FAT | 115 | 15 | 0.0212 | 520 | 229 | 13528 | DFNA5, SHROOM2, SOX1, ERBB2, NFYC, RPGRIP1, TBX1, GAS1, PRPH2, GPR98, TGFB2, FOXP2, ATP2B2, SIX1, PDGFRA | 1.7041 | 0.0586 | 65.5874 |
| GO:0016620~oxidoreductase activity, acting on the aldehyde or oxo group of donors, NAD or NADP as acceptor | MF_FAT | 116 | 4 | 0.0057 | 508 | 23 | 12983 | GAPDHS, ALDH6A1, ALDH18A1, ALDH1B1 | 4.4447 | 0.0587 | 60.4965 |
| GO:0006164~purine nucleotide biosynthetic process | BP_FAT | 117 | 11 | 0.0156 | 520 | 148 | 13528 | MTHFD1, TCIRG1, ATP2B2, ATP13A1, ATP4B, ATP9B, ATP11B, ATP5G2, ADCY10, GUCA2B, AMPD3 | 1.9336 | 0.0588 | 65.7236 |
| GO:0004713~protein tyrosine kinase activity | MF_FAT | 118 | 12 | 0.0170 | 508 | 166 | 12983 | FGFR1, OBSCN, RET, FGFR4, MAP2K2, ERBB2, PDGFRA, ALK, PIK3R4, EPHA1, SRC, CAMKK2 | 1.8475 | 0.0608 | 61.7951 |
| GO:0032559~adenyl ribonucleotide binding | MF_FAT | 119 | 71 | 0.1006 | 508 | 1497 | 12983 | PASK, AURKC, PKMYT1, KCNJ11, CAMKK2, MTHFD1, MAP3K6, KIF2C, ATP2B2, POLG2, ADCY10, IPMK, SIK2, CHUK, RET, MYH1, PHKG1, MYLK4, MYH4, ALK, MYH8, RENBP, CBWD1, HIPK1, ATP9B, RARS, PDGFRA, LRRK2, SRXN1, SMARCA4, KIF26B, ACVR1, FGFR1, FGFR4, ALDH18A1, ERBB2, MAPKAPK3, SHPK, SRC, MOV10, DDX49, MORC4, HSPA4, ENTPD3, UCK1, HCN4, PIK3R4, OBSCN, MSH3, TAOK2, ALPK2, MAP2K2, PIF1, ATP11B, NADK, YTHDC2, WRN, KIF3C, EPHA1, RIMKLA, APRT, ABCC9, ATP13A1, P2RX6, MAPK14, UCKL1, MYH13, NLRP14, DDX54, PGK1, GUK1 | 1.2121 | 0.0615 | 62.2562 |
| GO:0031091~platelet alpha granule | CC_FAT | 120 | 6 | 0.0085 | 489 | 56 | 12782 | PF4, SPARC, EGF, PROS1, TGFB2, FN1 | 2.8006 | 0.0618 | 59.0404 |
| GO:0042802~identical protein binding | MF_FAT | 121 | 34 | 0.0482 | 508 | 640 | 12983 | LDHB, SLC9A7, HNF1A, MASP1, HMGCR, ERBB2, HPS4, PASK, RNASEH1, CDSN, TGFB2, CLDN15, APOE, OXCT1, ENO3, POLG2, CHUK, GPD1, MSH3, ACY3, SNRPN, TBX1, TPD52L1, WRN, ACTN3, FOXP2, RENBP, TSC2, NLGN4X, PDGFRA, LRRK2, RNF40, SMARCA4, ACVR1 | 1.3577 | 0.0621 | 62.5934 |
| GO:0016458~gene silencing | BP_FAT | 122 | 6 | 0.0085 | 520 | 56 | 13528 | MOV10, RBM3, CELF1, PIWIL4, TNRC6A, SMARCA4 | 2.7874 | 0.0629 | 68.2797 |
| GO:0030955~potassium ion binding | MF_FAT | 123 | 10 | 0.0142 | 508 | 128 | 12983 | KCNQ5, SLC9A7, KCNK15, SLC24A3, ATP4B, HCN4, KCNK13, KCNJ11, KCNG2, KCNK4 | 1.9966 | 0.0632 | 63.2465 |
| GO:0031420~alkali metal ion binding | MF_FAT | 124 | 15 | 0.0212 | 508 | 228 | 12983 | SLC9A9, SLC9A7, KCNK15, SLC5A4, SLC12A3, ATP4B, KCNK13, KCNJ11, KCNK4, KCNQ5, SLC24A3, SLC13A4, SLC38A1, HCN4, KCNG2 | 1.6814 | 0.0638 | 63.6041 |
| GO:0003746~translation elongation factor activity | MF_FAT | 125 | 4 | 0.0057 | 508 | 24 | 12983 | TSFM, ABTB1, EIF5A, EEF1D | 4.2595 | 0.0653 | 64.4910 |
| GO:0006769~nicotinamide metabolic process | BP_FAT | 126 | 5 | 0.0071 | 520 | 40 | 13528 | GPD1, LDHB, PGD, NADK, DCXR | 3.2519 | 0.0660 | 70.1118 |
| GO:0046496~nicotinamide nucleotide metabolic process | BP_FAT | 127 | 5 | 0.0071 | 520 | 40 | 13528 | GPD1, LDHB, PGD, NADK, DCXR | 3.2519 | 0.0660 | 70.1118 |
| GO:0050777~negative regulation of immune response | BP_FAT | 128 | 4 | 0.0057 | 520 | 25 | 13528 | GPX2, MASP1, IL27RA, TGFB2 | 4.1625 | 0.0691 | 71.8151 |
| GO:0050680~negative regulation of epithelial cell proliferation | BP_FAT | 129 | 4 | 0.0057 | 520 | 25 | 13528 | CDKN1C, TSC2, GAS1, TGFB2 | 4.1625 | 0.0691 | 71.8151 |
| GO:0006261~DNA-dependent DNA replication | BP_FAT | 130 | 6 | 0.0085 | 520 | 58 | 13528 | TFAM, POLE2, POLA1, RNASEH1, WRN, POLG2 | 2.6912 | 0.0710 | 72.7907 |
| GO:0006007~glucose catabolic process | BP_FAT | 131 | 6 | 0.0085 | 520 | 58 | 13528 | GAPDHS, LDHB, PGD, ENO3, PDHA1, PGK1 | 2.6912 | 0.0710 | 72.7907 |
| GO:0009820~alkaloid metabolic process | BP_FAT | 132 | 5 | 0.0071 | 520 | 41 | 13528 | GPD1, LDHB, PGD, NADK, DCXR | 3.1726 | 0.0711 | 72.8296 |
| GO:0043583~ear development | BP_FAT | 133 | 8 | 0.0113 | 520 | 95 | 13528 | ATP2B2, DFNA5, SHROOM2, SIX1, PDGFRA, TBX1, GAS1, GPR98 | 2.1908 | 0.0724 | 73.5154 |
| GO:0004672~protein kinase activity | MF_FAT | 134 | 32 | 0.0453 | 508 | 606 | 12983 | PRKAG3, FGFR1, FGFR4, ERBB2, PASK, MAPKAPK3, AURKC, PKMYT1, SRC, CAMKK2, TGFB2, MAP3K6, PACSIN1, SIK2, PIK3R4, CHUK, OBSCN, RET, TAOK2, ALPK2, PHKG1, MAP2K2, MYLK4, DUSP21, ALK, EPHA1, MNAT1, HIPK1, MAPK14, PDGFRA, LRRK2, ACVR1 | 1.3495 | 0.0749 | 69.7352 |
| GO:0009925~basal plasma membrane | CC_FAT | 135 | 4 | 0.0057 | 489 | 26 | 12782 | ERBB2IP, ERBB2, SLC16A8, FKBPL | 4.0214 | 0.0751 | 66.4609 |
| GO:0030878~thyroid gland development | BP_FAT | 136 | 3 | 0.0042 | 520 | 12 | 13528 | PAX8, SIX1, TBX1 | 6.5038 | 0.0752 | 74.8930 |
| GO:0002675~positive regulation of acute inflammatory response | BP_FAT | 137 | 3 | 0.0042 | 520 | 12 | 13528 | OSM, FCER1A, IL6 | 6.5038 | 0.0752 | 74.8930 |
| GO:0019362~pyridine nucleotide metabolic process | BP_FAT | 138 | 5 | 0.0071 | 520 | 42 | 13528 | GPD1, LDHB, PGD, NADK, DCXR | 3.0971 | 0.0763 | 75.4024 |
| GO:0007368~determination of left/right symmetry | BP_FAT | 139 | 5 | 0.0071 | 520 | 42 | 13528 | DISP1, TBX1, IFT52, IFT88, ACVR1 | 3.0971 | 0.0763 | 75.4024 |
| GO:0005267~potassium channel activity | MF_FAT | 140 | 10 | 0.0142 | 508 | 133 | 12983 | SHKBP1, KCNQ5, ABCC9, KCNK15, HCN4, KCNK13, KCNJ11, KCTD7, KCNG2, KCNK4 | 1.9216 | 0.0764 | 70.4400 |
| GO:0001882~nucleoside binding | MF_FAT | 141 | 75 | 0.1062 | 508 | 1612 | 12983 | ACOX1, PASK, AURKC, PKMYT1, KCNJ11, CNGB3, CAMKK2, MTHFD1, MAP3K6, KIF2C, ATP2B2, RHOB, POLG2, ADCY10, IPMK, SIK2, CHUK, RET, MYH1, PHKG1, MYLK4, MYH4, ALK, MYH8, RENBP, CBWD1, HIPK1, ATP9B, RARS, PDGFRA, LRRK2, SRXN1, SMARCA4, KIF26B, ACVR1, FGFR1, FGFR4, ALDH18A1, ERBB2, POLA1, MAPKAPK3, SHPK, SRC, MOV10, DDX49, MORC4, HSPA4, ENTPD3, UCK1, HCN4, PIK3R4, OBSCN, MSH3, TAOK2, ALPK2, MAP2K2, PIF1, ATP11B, NADK, YTHDC2, WRN, KIF3C, EPHA1, RIMKLA, APRT, ABCC9, ATP13A1, P2RX6, MAPK14, UCKL1, MYH13, NLRP14, DDX54, PGK1, GUK1 | 1.1891 | 0.0765 | 70.4889 |
| GO:0004368~glycerol-3-phosphate dehydrogenase activity | MF_FAT | 142 | 2 | 0.0028 | 508 | 2 | 12983 | GPD2, GPD1 | 25.5571 | 0.0766 | 70.5463 |
| GO:0016500~protein-hormone receptor activity | MF_FAT | 143 | 3 | 0.0042 | 508 | 12 | 12983 | LEPR, PAX8, LGR5 | 6.3893 | 0.0776 | 71.0181 |
| GO:0006468~protein amino acid phosphorylation | BP_FAT | 144 | 34 | 0.0482 | 520 | 667 | 13528 | FGFR1, FGFR4, ERBB2, PASK, DRD4, FPR1, MAPKAPK3, AURKC, PKMYT1, SRC, CAMKK2, TGFB2, MAP3K6, EGF, SIK2, PIK3R4, CHUK, FCER1A, OBSCN, RET, TAOK2, ALPK2, PHKG1, MAP2K2, MYLK4, ALK, EPHA1, OSM, HIPK1, MAPK14, PDGFRA, ERC1, LRRK2, ACVR1 | 1.3261 | 0.0780 | 76.2096 |
| GO:0030278~regulation of ossification | BP_FAT | 145 | 7 | 0.0099 | 520 | 78 | 13528 | IL6, OXT, JUND, SKI, TWIST2, TGFB2, ACVR1 | 2.3347 | 0.0786 | 76.4903 |
| GO:0007179~transforming growth factor beta receptor signaling pathway | BP_FAT | 146 | 6 | 0.0085 | 520 | 60 | 13528 | SMAD9, LTBP3, MSTN, TGFBRAP1, TGFB2, ACVR1 | 2.6015 | 0.0796 | 76.9340 |
| GO:0030902~hindbrain development | BP_FAT | 147 | 6 | 0.0085 | 520 | 60 | 13528 | ATP2B2, SMAD9, GAS1, KLHL1, SMARCA4, FOXP2 | 2.6015 | 0.0796 | 76.9340 |
| GO:0007264~small GTPase mediated signal transduction | BP_FAT | 148 | 18 | 0.0255 | 520 | 305 | 13528 | GTPBP2, ARHGDIG, RALGPS2, RAB8B, MAP2K2, MAPKAPK3, LOC220077, SRC, ARHGEF11, NRAS, RAB32, APOE, MAPK14, RHOB, LRRK2, ARL5C, RAPGEFL1, KNDC1 | 1.5353 | 0.0796 | 76.9411 |
| GO:0035295~tube development | BP_FAT | 149 | 14 | 0.0198 | 520 | 220 | 13528 | FGFR4, RET, FGF7, FOXP2, WNT4, GPC3, SIX1, TSC2, PDGFRA, IFT52, EGF, IPMK, IFT88, ACVR1 | 1.6555 | 0.0814 | 77.7208 |
| GO:0045667~regulation of osteoblast differentiation | BP_FAT | 150 | 5 | 0.0071 | 520 | 43 | 13528 | IL6, JUND, SKI, TWIST2, ACVR1 | 3.0250 | 0.0817 | 77.8240 |
| GO:0009799~determination of symmetry | BP_FAT | 151 | 5 | 0.0071 | 520 | 43 | 13528 | DISP1, TBX1, IFT52, IFT88, ACVR1 | 3.0250 | 0.0817 | 77.8240 |
| GO:0001838~embryonic epithelial tube formation | BP_FAT | 152 | 5 | 0.0071 | 520 | 43 | 13528 | WNT4, RET, TSC2, IFT52, IPMK | 3.0250 | 0.0817 | 77.8240 |
| GO:0009855~determination of bilateral symmetry | BP_FAT | 153 | 5 | 0.0071 | 520 | 43 | 13528 | DISP1, TBX1, IFT52, IFT88, ACVR1 | 3.0250 | 0.0817 | 77.8240 |
| GO:0005524~ATP binding | MF_FAT | 154 | 69 | 0.0977 | 508 | 1477 | 12983 | PASK, AURKC, PKMYT1, KCNJ11, CAMKK2, MTHFD1, MAP3K6, KIF2C, ATP2B2, POLG2, ADCY10, IPMK, SIK2, CHUK, RET, MYH1, PHKG1, MYLK4, MYH4, ALK, MYH8, RENBP, CBWD1, HIPK1, ATP9B, RARS, PDGFRA, LRRK2, SRXN1, SMARCA4, KIF26B, ACVR1, FGFR1, FGFR4, ALDH18A1, ERBB2, MAPKAPK3, SHPK, SRC, MOV10, DDX49, MORC4, HSPA4, ENTPD3, UCK1, PIK3R4, OBSCN, MSH3, TAOK2, ALPK2, MAP2K2, PIF1, ATP11B, NADK, YTHDC2, WRN, KIF3C, EPHA1, RIMKLA, ABCC9, ATP13A1, P2RX6, MAPK14, UCKL1, MYH13, NLRP14, DDX54, PGK1, GUK1 | 1.1939 | 0.0838 | 73.8694 |
| GO:0030141~secretory granule | CC_FAT | 155 | 12 | 0.0170 | 489 | 180 | 12782 | PAM, HCRT, OXT, HPS4, STXBP2, PF4, SPARC, EGF, GAL, PROS1, TGFB2, FN1 | 1.7426 | 0.0841 | 70.7513 |
| GO:0001883~purine nucleoside binding | MF_FAT | 156 | 74 | 0.1048 | 508 | 1601 | 12983 | ACOX1, PASK, AURKC, PKMYT1, KCNJ11, CNGB3, CAMKK2, MTHFD1, MAP3K6, KIF2C, ATP2B2, RHOB, POLG2, ADCY10, IPMK, SIK2, CHUK, RET, MYH1, PHKG1, MYLK4, MYH4, ALK, MYH8, RENBP, CBWD1, HIPK1, ATP9B, RARS, PDGFRA, LRRK2, SRXN1, SMARCA4, KIF26B, ACVR1, FGFR1, FGFR4, ALDH18A1, ERBB2, MAPKAPK3, SHPK, SRC, MOV10, DDX49, MORC4, HSPA4, ENTPD3, UCK1, HCN4, PIK3R4, OBSCN, MSH3, TAOK2, ALPK2, MAP2K2, PIF1, ATP11B, NADK, YTHDC2, WRN, KIF3C, EPHA1, RIMKLA, APRT, ABCC9, ATP13A1, P2RX6, MAPK14, UCKL1, MYH13, NLRP14, DDX54, PGK1, GUK1 | 1.1813 | 0.0859 | 74.8053 |
| GO:0014066~regulation of phosphoinositide 3-kinase cascade | BP_FAT | 157 | 3 | 0.0042 | 520 | 13 | 13528 | ERBB2, TSC2, TGFB2 | 6.0036 | 0.0867 | 79.8687 |
| GO:0035148~tube lumen formation | BP_FAT | 158 | 5 | 0.0071 | 520 | 44 | 13528 | WNT4, RET, TSC2, IFT52, IPMK | 2.9563 | 0.0872 | 80.0901 |
| GO:0015711~organic anion transport | BP_FAT | 159 | 5 | 0.0071 | 520 | 44 | 13528 | SLC16A1, SLC16A7, SLC16A8, SLC25A22, SLC1A1 | 2.9563 | 0.0872 | 80.0901 |
| GO:0042330~taxis | BP_FAT | 160 | 11 | 0.0156 | 520 | 160 | 13528 | IL6, MAPK14, TSC2, FPR1, DEFA1, PF4, LECT2, CMTM3, CMTM6, CCL7, TGFB2 | 1.7886 | 0.0884 | 80.5331 |
| GO:0006935~chemotaxis | BP_FAT | 161 | 11 | 0.0156 | 520 | 160 | 13528 | IL6, MAPK14, TSC2, FPR1, DEFA1, PF4, LECT2, CMTM3, CMTM6, CCL7, TGFB2 | 1.7886 | 0.0884 | 80.5331 |
| GO:0001540~beta-amyloid binding | MF_FAT | 162 | 3 | 0.0042 | 508 | 13 | 12983 | APOE, CST3, TGFB2 | 5.8978 | 0.0894 | 76.2141 |
| GO:0003779~actin binding | MF_FAT | 163 | 19 | 0.0269 | 508 | 326 | 12983 | INF2, IPP, MYL4, SHROOM2, MYBPC2, MYH1, CAPZA1, MYH4, ACTN3, MYH8, TNNI2, VCL, KLHL1, SMTN, XIRP1, MYH13, TMSB4Y, LMOD2, PLEC | 1.4895 | 0.0901 | 76.5191 |
| GO:0046164~alcohol catabolic process | BP_FAT | 164 | 7 | 0.0099 | 520 | 81 | 13528 | GAPDHS, GPD1, LDHB, PGD, ENO3, PDHA1, PGK1 | 2.2482 | 0.0905 | 81.3130 |
| GO:0046873~metal ion transmembrane transporter activity | MF_FAT | 165 | 19 | 0.0269 | 508 | 328 | 12983 | SLC9A7, KCNK15, SHROOM2, CACNG4, KCNK13, ITPR3, CNGB3, KCNJ11, KCTD7, KCNK4, SHKBP1, ATP2B2, KCNQ5, ABCC9, P2RX6, RYR3, SLC13A4, HCN4, KCNG2 | 1.4804 | 0.0927 | 77.5225 |
| GO:0050878~regulation of body fluid levels | BP_FAT | 166 | 10 | 0.0142 | 520 | 141 | 13528 | IL6, NFE2, OXT, HPS4, TFPI, C4BPB, PF4, GUCA2B, PROS1, TP73 | 1.8451 | 0.0929 | 82.1546 |
| GO:0002009~morphogenesis of an epithelium | BP_FAT | 167 | 8 | 0.0113 | 520 | 101 | 13528 | WNT4, RET, GPC3, SIX1, TSC2, IFT52, IPMK, CHUK | 2.0606 | 0.0932 | 82.2629 |
| GO:0060341~regulation of cellular localization | BP_FAT | 168 | 15 | 0.0212 | 520 | 248 | 13528 | FCER1A, HCRT, IL6, RAB8B, HNF1A, NFKBIE, OXT, DRD4, PKIG, STXBP2, TACC3, KCNJ11, TGFB2, OSM, EGF | 1.5735 | 0.0962 | 83.2711 |
| GO:0030018~Z disc | CC_FAT | 169 | 5 | 0.0071 | 489 | 46 | 12782 | JUP, OBSCN, SYNC, PDLIM3, MYOZ2 | 2.8412 | 0.0976 | 76.2260 |
| GO:0005795~Golgi stack | CC_FAT | 170 | 5 | 0.0071 | 489 | 46 | 12782 | GLG1, GALNT2, ARSE, B3GALT6, CLIP3 | 2.8412 | 0.0976 | 76.2260 |
| GO:0033135~regulation of peptidyl-serine phosphorylation | BP_FAT | 171 | 3 | 0.0042 | 520 | 14 | 13528 | OSM, IL6, DOCK7 | 5.5747 | 0.0986 | 84.0544 |
| GO:0018108~peptidyl-tyrosine phosphorylation | BP_FAT | 172 | 5 | 0.0071 | 520 | 46 | 13528 | OSM, ERBB2, PDGFRA, EGF, SRC | 2.8278 | 0.0989 | 84.1506 |

**Table S4. The result of DAVID GO enrichment for 287 DEGs from PCOS6.**

| **GO_term** | **GO_category** | **GO_rank** | **Num_of_symbols_in_list_in_GO** | | **Percentage_of_symbols_in_list** | **Num_of _symbols_in_bglist_in_GO** | **Num_of_symbols_in_list** | **Num_of_symbols_in_bglist** | **Symbols_in_list** | **Fold_enrichment** | **P value** | **FDR** |
| --- | --- | --- | --- | --- | --- | --- | --- | --- | --- | --- | --- | --- |
| GO:0005578~proteinaceous extracellular matrix | CC_FAT | 1 | 19 | 0.0667 | | 204 | 320 | 12782 | FLRT2, SPARCL1, CD248, FBN1, EFEMP1, MGP, NID1, MMP2, ECM1, COL5A2, BGN, FREM1, KAL1, TGFBI, COL6A1, COL1A1, LOX, ADAMTS5, FN1 | 3.7203 | 3.44 E-06 | 0.0046 |
| GO:0031012~extracellular matrix | CC_FAT | 2 | 19 | 0.0667 | | 204 | 345 | 12782 | FLRT2, SPARCL1, CD248, FBN1, EFEMP1, MGP, NID1, MMP2, ECM1, COL5A2, BGN, FREM1, KAL1, TGFBI, COL6A1, COL1A1, LOX, ADAMTS5, FN1 | 3.4507 | 9.77 E-06 | 0.0131 |
| GO:0009725~response to hormone stimulus | BP_FAT | 3 | 20 | 0.0702 | | 229 | 367 | 13528 | A2M, CCL2, LDLR, GNRH1, GGH, MGP, CRIPAK, ALDH1A2, KRT19, BTG2, PIAS3, PDGFRA, FABP3, GNB4, LOX, COL1A1, PIK3R3, RBM14, EIF2B3, CCNA2 | 3.2193 | 1.47 E-05 | 0.0249 |
| GO:0009719~response to endogenous stimulus | BP_FAT | 4 | 20 | 0.0702 | | 229 | 405 | 13528 | A2M, CCL2, LDLR, GNRH1, GGH, MGP, CRIPAK, ALDH1A2, KRT19, BTG2, PIAS3, PDGFRA, FABP3, GNB4, LOX, COL1A1, PIK3R3, RBM14, EIF2B3, CCNA2 | 2.9172 | 5.61 E-05 | 0.0952 |
| GO:0044421~extracellular region part | CC_FAT | 5 | 32 | 0.1123 | | 204 | 960 | 12782 | A2M, FGF7, CCL2, LDLR, CD248, MMP2, VCAM1, KAL1, TGFBI, COL6A1, GDF9, LOX, FN1, ICAM1, FLRT2, GNRH1, SPARCL1, FBN1, EFEMP1, GGH, MGP, IGF1, NID1, COL5A2, ECM1, TNFSF10, BGN, FREM1, COL1A1, IGFBP3, ADAMTS5, IGFBP5 | 2.0886 | 0.0001 | 0.1543 |
| GO:0001503~ossification | BP_FAT | 6 | 10 | 0.0351 | | 229 | 115 | 13528 | GABBR1, IGF1, MGP, COL1A1, GPNMB, COL5A2, IGFBP3, MMP2, CDH11, IGFBP5 | 5.1369 | 0.0001 | 0.2374 |
| GO:0060348~bone development | BP_FAT | 7 | 10 | 0.0351 | | 229 | 123 | 13528 | GABBR1, IGF1, MGP, COL1A1, GPNMB, COL5A2, IGFBP3, MMP2, CDH11, IGFBP5 | 4.8028 | 0.0002 | 0.3953 |
| GO:0005739~mitochondrion | CC_FAT | 8 | 33 | 0.1158 | | 204 | 1087 | 12782 | NDUFB6, MRPS15, NDUFB8, CA5B, PNPT1, KIAA0101, NDUFAB1, C14ORF2, KARS, MRPL20, FAM65B, UQCR10, CISD1, NUDT9, TGM2, ATP5O, XAF1, NDUFA4, MRPS23, MAOB, LYRM4, SIRT4, TP53, VDAC3, CPT1A, RAB32, SDHB, MTRF1, POLDIP2, ATP5C1, TOMM22, UQCRB, VPS25 | 1.9022 | 0.0005 | 0.6282 |
| GO:0010033~response to organic substance | BP_FAT | 9 | 26 | 0.0912 | | 229 | 721 | 13528 | A2M, CCL2, LDLR, C1S, GCH1, EDNRA, ALDH1A2, LOX, PIK3R3, CCNA2, EIF2B3, GNRH1, GGH, MGP, CRIPAK, SMAD2, CDK5, CD83, KRT19, BTG2, PIAS3, FABP3, PDGFRA, GNB4, COL1A1, RBM14 | 2.1303 | 0.0005 | 0.8334 |
| GO:0000779~condensed chromosome, centromeric region | CC_FAT | 10 | 7 | 0.0246 | | 204 | 66 | 12782 | MAD2L1, SGOL2, SGOL1, CENPV, NDC80, NUP37, CENPK | 6.6454 | 0.0006 | 0.8170 |
| GO:0001649~osteoblast differentiation | BP_FAT | 11 | 6 | 0.0211 | | 229 | 42 | 13528 | GABBR1, IGF1, COL1A1, GPNMB, IGFBP3, IGFBP5 | 8.4392 | 0.0007 | 1.1239 |
| GO:0022904~respiratory electron transport chain | BP_FAT | 12 | 7 | 0.0246 | | 229 | 64 | 13528 | NDUFA4, SDHB, UQCR10, NDUFB6, NDUFB8, NDUFAB1, UQCRB | 6.4612 | 0.0007 | 1.2008 |
| GO:0045333~cellular respiration | BP_FAT | 13 | 8 | 0.0281 | | 229 | 97 | 13528 | NDUFA4, SDHB, MDH1B, UQCR10, NDUFB6, NDUFB8, NDUFAB1, UQCRB | 4.8721 | 0.0013 | 2.1030 |
| GO:0006119~oxidative phosphorylation | BP_FAT | 14 | 8 | 0.0281 | | 229 | 98 | 13528 | NDUFA4, UQCR10, NDUFB6, NDUFB8, NDUFAB1, ATP5C1, ATP5O, UQCRB | 4.8224 | 0.0013 | 2.2316 |
| GO:0016942~insulin-like growth factor binding protein complex | CC_FAT | 15 | 3 | 0.0105 | | 204 | 4 | 12782 | IGF1, IGFBP3, IGFBP5 | 46.9926 | 0.0015 | 1.9547 |
| GO:0048545~response to steroid hormone stimulus | BP_FAT | 16 | 11 | 0.0386 | | 229 | 192 | 13528 | ALDH1A2, A2M, KRT19, CCL2, LDLR, GNRH1, PDGFRA, CRIPAK, COL1A1, LOX, CCNA2 | 3.3845 | 0.0016 | 2.6040 |
| GO:0031967~organelle envelope | CC_FAT | 17 | 21 | 0.0737 | | 204 | 620 | 12782 | NDUFA4, NDUFB6, NDUFB8, PNPT1, MAOB, NDUFAB1, NUP93, NXF1, VDAC3, CPT1A, EDNRA, SDHB, UQCR10, CISD1, MAD2L1, ATP5C1, ATP5O, TOMM22, NUP37, TMPO, UQCRB | 2.1222 | 0.0021 | 2.7194 |
| GO:0031975~envelope | CC_FAT | 18 | 21 | 0.0737 | | 204 | 622 | 12782 | NDUFA4, NDUFB6, NDUFB8, PNPT1, MAOB, NDUFAB1, NUP93, NXF1, VDAC3, CPT1A, EDNRA, SDHB, UQCR10, CISD1, MAD2L1, ATP5C1, ATP5O, TOMM22, NUP37, TMPO, UQCRB | 2.1154 | 0.0021 | 2.8220 |
| GO:0030155~regulation of cell adhesion | BP_FAT | 19 | 9 | 0.0316 | | 229 | 137 | 13528 | ICAM1, EMCN, TGFBI, TGM2, CDK6, NID1, CYTH3, COL1A1, SERPINI1 | 3.8808 | 0.0022 | 3.7267 |
| GO:0007059~chromosome segregation | BP_FAT | 20 | 7 | 0.0246 | | 229 | 81 | 13528 | RAD51C, MAD2L1, SGOL2, DLGAP5, SGOL1, NDC80, NUP37 | 5.1052 | 0.0024 | 4.0466 |
| GO:0042775~mitochondrial ATP synthesis coupled electron transport | BP_FAT | 21 | 6 | 0.0211 | | 229 | 56 | 13528 | NDUFA4, UQCR10, NDUFB6, NDUFB8, NDUFAB1, UQCRB | 6.3294 | 0.0025 | 4.1114 |
| GO:0042773~ATP synthesis coupled electron transport | BP_FAT | 22 | 6 | 0.0211 | | 229 | 56 | 13528 | NDUFA4, UQCR10, NDUFB6, NDUFB8, NDUFAB1, UQCRB | 6.3294 | 0.0025 | 4.1114 |
| GO:0044420~extracellular matrix part | CC_FAT | 23 | 8 | 0.0281 | | 204 | 117 | 12782 | FREM1, FBN1, COL6A1, NID1, COL1A1, LOX, COL5A2, FN1 | 4.2842 | 0.0026 | 3.4408 |
| GO:0005178~integrin binding | MF_FAT | 24 | 6 | 0.0211 | | 213 | 59 | 12983 | VCAM1, ICAM1, TGFBI, ACTN1, GPNMB, ADAMTS5 | 6.1986 | 0.0027 | 3.8375 |
| GO:0048145~regulation of fibroblast proliferation | BP_FAT | 25 | 5 | 0.0175 | | 229 | 35 | 13528 | PDGFRA, TP53, IGF1, CDK6, CCNA2 | 8.4392 | 0.0027 | 4.5338 |
| GO:0005201~extracellular matrix structural constituent | MF_FAT | 26 | 7 | 0.0246 | | 213 | 86 | 12983 | BGN, KAL1, FBN1, MGP, COL1A1, COL5A2, FN1 | 4.9613 | 0.0028 | 3.9703 |
| GO:0044429~mitochondrial part | CC_FAT | 27 | 20 | 0.0702 | | 204 | 595 | 12782 | NDUFA4, MRPS15, NDUFB6, NDUFB8, PNPT1, MAOB, SIRT4, NDUFAB1, VDAC3, CPT1A, KARS, MRPL20, SDHB, UQCR10, CISD1, POLDIP2, ATP5C1, ATP5O, TOMM22, UQCRB | 2.1061 | 0.0030 | 3.9031 |
| GO:0015980~energy derivation by oxidation of organic compounds | BP_FAT | 28 | 9 | 0.0316 | | 229 | 144 | 13528 | NDUFA4, SDHB, MDH1B, UQCR10, NDUFB6, PYGL, NDUFB8, NDUFAB1, UQCRB | 3.6921 | 0.0030 | 5.0438 |
| GO:0022402~cell cycle process | BP_FAT | 29 | 20 | 0.0702 | | 229 | 565 | 13528 | BCAT1, RAD51C, SGOL2, DLGAP5, SGOL1, SF1, TP53, CDK6, NDC80, PBK, CEP55, ANAPC11, TCF7L2, PSMB5, MAD2L1, KIF20B, CENPV, CAMK2D, NUP37, CCNA2 | 2.0911 | 0.0033 | 5.4606 |
| GO:0005743~mitochondrial inner membrane | CC_FAT | 30 | 13 | 0.0456 | | 204 | 306 | 12782 | NDUFA4, SDHB, UQCR10, NDUFB6, NDUFB8, MAOB, ATP5C1, NDUFAB1, TOMM22, ATP5O, VDAC3, CPT1A, UQCRB | 2.6619 | 0.0036 | 4.6998 |
| GO:0007155~cell adhesion | BP_FAT | 31 | 23 | 0.0807 | | 229 | 700 | 13528 | FLRT2, ICAM1, EMCN, SVEP1, CCL2, NRXN3, NLGN1, MGP, ACTN1, NID1, CDK5, PCDH18, VCAM1, TNFAIP6, HSPB11, FREM1, PCDHB16, KAL1, TGFBI, COL6A1, GPNMB, FN1, CDH11 | 1.9410 | 0.0037 | 6.0277 |
| GO:0022610~biological adhesion | BP_FAT | 32 | 23 | 0.0807 | | 229 | 701 | 13528 | FLRT2, ICAM1, EMCN, SVEP1, CCL2, NRXN3, NLGN1, MGP, ACTN1, NID1, CDK5, PCDH18, VCAM1, TNFAIP6, HSPB11, FREM1, PCDHB16, KAL1, TGFBI, COL6A1, GPNMB, FN1, CDH11 | 1.9382 | 0.0037 | 6.1278 |
| GO:0044455~mitochondrial membrane part | CC_FAT | 33 | 8 | 0.0281 | | 204 | 125 | 12782 | NDUFA4, NDUFB6, NDUFB8, NDUFAB1, ATP5C1, TOMM22, ATP5O, UQCRB | 4.0100 | 0.0038 | 4.9408 |
| GO:0000278~mitotic cell cycle | BP_FAT | 34 | 15 | 0.0526 | | 229 | 370 | 13528 | BCAT1, DLGAP5, SGOL1, CDK6, NDC80, PBK, CEP55, ANAPC11, PSMB5, MAD2L1, CENPV, KIF20B, CAMK2D, NUP37, CCNA2 | 2.3949 | 0.0041 | 6.7039 |
| GO:0000280~nuclear division | BP_FAT | 35 | 11 | 0.0386 | | 229 | 220 | 13528 | MAD2L1, DLGAP5, SGOL1, CENPV, KIF20B, NDC80, NUP37, ANAPC11, CEP55, PBK, CCNA2 | 2.9537 | 0.0042 | 6.8386 |
| GO:0007067~mitosis | BP_FAT | 36 | 11 | 0.0386 | | 229 | 220 | 13528 | MAD2L1, DLGAP5, SGOL1, CENPV, KIF20B, NDC80, NUP37, ANAPC11, CEP55, PBK, CCNA2 | 2.9537 | 0.0042 | 6.8386 |
| GO:0022403~cell cycle phase | BP_FAT | 37 | 16 | 0.0561 | | 229 | 414 | 13528 | BCAT1, RAD51C, SGOL2, DLGAP5, SGOL1, CDK6, NDC80, PBK, CEP55, ANAPC11, MAD2L1, CENPV, KIF20B, CAMK2D, NUP37, CCNA2 | 2.2831 | 0.0044 | 7.1937 |
| GO:0000087~M phase of mitotic cell cycle | BP_FAT | 38 | 11 | 0.0386 | | 229 | 224 | 13528 | MAD2L1, DLGAP5, SGOL1, CENPV, KIF20B, NDC80, NUP37, ANAPC11, CEP55, PBK, CCNA2 | 2.9010 | 0.0047 | 7.7231 |
| GO:0042063~gliogenesis | BP_FAT | 39 | 6 | 0.0211 | | 229 | 65 | 13528 | PDGFRA, IGF1, CDK6, C1S, EIF2B3, CDK5 | 5.4530 | 0.0047 | 7.7297 |
| GO:0051270~regulation of cell motion | BP_FAT | 40 | 10 | 0.0351 | | 229 | 193 | 13528 | ICAM1, ENPP2, PDGFRA, IGF1, ACTN1, ABHD2, SEMA3A, IGFBP3, CDK5, IGFBP5 | 3.0608 | 0.0055 | 8.8811 |
| GO:0048285~organelle fission | BP_FAT | 41 | 11 | 0.0386 | | 229 | 229 | 13528 | MAD2L1, DLGAP5, SGOL1, CENPV, KIF20B, NDC80, NUP37, ANAPC11, CEP55, PBK, CCNA2 | 2.8376 | 0.0055 | 8.9538 |
| GO:0007049~cell cycle | BP_FAT | 42 | 24 | 0.0842 | | 229 | 776 | 13528 | BCAT1, RAD51C, SGOL2, NASP, LIN9, DLGAP5, SGOL1, SF1, TP53, CDK6, NDC80, PBK, CEP55, ANAPC11, CDK5, TCF7L2, PSMB5, TSPYL2, MAD2L1, KIF20B, CENPV, CAMK2D, NUP37, CCNA2 | 1.8270 | 0.0060 | 9.7785 |
| GO:0019866~organelle inner membrane | CC_FAT | 43 | 13 | 0.0456 | | 204 | 329 | 12782 | NDUFA4, SDHB, UQCR10, NDUFB6, NDUFB8, MAOB, ATP5C1, NDUFAB1, TOMM22, ATP5O, VDAC3, CPT1A, UQCRB | 2.4758 | 0.0064 | 8.2082 |
| GO:0043062~extracellular structure organization | BP_FAT | 44 | 9 | 0.0316 | | 229 | 163 | 13528 | NRXN3, PCDHB16, TGFBI, NLGN1, PDGFRA, NID1, COL1A1, LOX, COL5A2 | 3.2618 | 0.0064 | 10.3307 |
| GO:0070469~respiratory chain | CC_FAT | 45 | 6 | 0.0211 | | 204 | 75 | 12782 | NDUFA4, UQCR10, NDUFB6, NDUFB8, NDUFAB1, UQCRB | 5.0125 | 0.0068 | 8.6694 |
| GO:0005740~mitochondrial envelope | CC_FAT | 46 | 15 | 0.0526 | | 204 | 419 | 12782 | NDUFA4, NDUFB6, NDUFB8, PNPT1, MAOB, NDUFAB1, VDAC3, CPT1A, SDHB, UQCR10, CISD1, ATP5C1, ATP5O, TOMM22, UQCRB | 2.2431 | 0.0071 | 9.0346 |
| GO:0048660~regulation of smooth muscle cell proliferation | BP_FAT | 47 | 5 | 0.0175 | | 229 | 46 | 13528 | SF1, TGM2, IGF1, IGFBP3, IGFBP5 | 6.4211 | 0.0074 | 11.7955 |
| GO:0045132~meiotic chromosome segregation | BP_FAT | 48 | 3 | 0.0105 | | 229 | 8 | 13528 | RAD51C, SGOL2, SGOL1 | 22.1528 | 0.0074 | 11.8492 |
| GO:0001501~skeletal system development | BP_FAT | 49 | 13 | 0.0456 | | 229 | 319 | 13528 | HOXB3, FBN1, GABBR1, PDGFRA, IGF1, MGP, COL1A1, GPNMB, COL5A2, IGFBP3, MMP2, CDH11, IGFBP5 | 2.4074 | 0.0079 | 12.5680 |
| GO:0005509~calcium ion binding | MF_FAT | 50 | 26 | 0.0912 | | 213 | 919 | 12983 | CLTA, LDLR, CACHD1, S100A8, CD248, NDUFAB1, C1S, MMP2, GCH1, GALNT10, PCDHB16, TGM2, SVEP1, TRPC6, NRXN3, SPARCL1, FBN1, EFEMP1, CBL, ACTN1, MGP, NID1, PCDH18, FREM1, MYLK, CDH11 | 1.7245 | 0.0082 | 11.1537 |
| GO:0048146~positive regulation of fibroblast proliferation | BP_FAT | 51 | 4 | 0.0140 | | 229 | 26 | 13528 | PDGFRA, IGF1, CDK6, CCNA2 | 9.0883 | 0.0092 | 14.5552 |
| GO:0018149~peptide cross-linking | BP_FAT | 52 | 4 | 0.0140 | | 229 | 26 | 13528 | BGN, TGM2, CSTA, FN1 | 9.0883 | 0.0092 | 14.5552 |
| GO:0060325~face morphogenesis | BP_FAT | 53 | 3 | 0.0105 | | 229 | 9 | 13528 | PDGFRA, COL1A1, MMP2 | 19.6914 | 0.0094 | 14.8304 |
| GO:0000279~M phase | BP_FAT | 54 | 13 | 0.0456 | | 229 | 329 | 13528 | RAD51C, DLGAP5, SGOL2, SGOL1, NDC80, ANAPC11, PBK, CEP55, MAD2L1, KIF20B, CENPV, NUP37, CCNA2 | 2.3342 | 0.0100 | 15.7087 |
| GO:0031974~membrane-enclosed lumen | CC_FAT | 55 | 43 | 0.1509 | | 204 | 1856 | 12782 | ING5, A2M, MRPS15, LIN9, PNPT1, RBM5, NDUFAB1, MED24, NFKB1, INTS3, ANAPC11, TCF7L2, KARS, MRPL20, TSPYL2, RPP30, MBNL3, GEMIN6, CCNA2, TSEN15, FN1, ELP4, POLR3G, ACTB, ZBTB20, SIRT4, SF1, TP53, CCNL1, ACTN1, IGF1, SMAD2, NXF1, DACH1, PIAS3, SFPQ, POLDIP2, DDX50, KIF20B, ATP5C1, CIRBP, RBM14, VPS25 | 1.4516 | 0.0101 | 12.7247 |
| GO:0031966~mitochondrial membrane | CC_FAT | 56 | 14 | 0.0491 | | 204 | 394 | 12782 | NDUFA4, SDHB, UQCR10, CISD1, NDUFB6, NDUFB8, MAOB, ATP5C1, NDUFAB1, TOMM22, ATP5O, VDAC3, CPT1A, UQCRB | 2.2264 | 0.0102 | 12.8737 |
| GO:0032269~negative regulation of cellular protein metabolic process | BP_FAT | 57 | 9 | 0.0316 | | 229 | 180 | 13528 | PSMB5, A2M, MAD2L1, NFKB1, ANAPC11, EIF2B3, IGFBP3, CDK5, IGFBP5 | 2.9537 | 0.0113 | 17.5681 |
| GO:0051301~cell division | BP_FAT | 58 | 12 | 0.0421 | | 229 | 295 | 13528 | MAD2L1, SGOL2, SGOL1, CENPV, KIF20B, CDK6, NDC80, NUP37, ANAPC11, CEP55, CDK5, CCNA2 | 2.4030 | 0.0115 | 17.8703 |
| GO:0043233~organelle lumen | CC_FAT | 59 | 42 | 0.1474 | | 204 | 1820 | 12782 | ING5, A2M, MRPS15, LIN9, RBM5, NDUFAB1, MED24, NFKB1, INTS3, ANAPC11, TCF7L2, KARS, MRPL20, TSPYL2, RPP30, MBNL3, GEMIN6, CCNA2, TSEN15, FN1, ELP4, POLR3G, ACTB, ZBTB20, SIRT4, SF1, TP53, CCNL1, ACTN1, IGF1, SMAD2, NXF1, DACH1, PIAS3, SFPQ, POLDIP2, DDX50, KIF20B, ATP5C1, CIRBP, RBM14, VPS25 | 1.4459 | 0.0119 | 14.8324 |
| GO:0022900~electron transport chain | BP_FAT | 60 | 7 | 0.0246 | | 229 | 114 | 13528 | NDUFA4, SDHB, UQCR10, NDUFB6, NDUFB8, NDUFAB1, UQCRB | 3.6274 | 0.0127 | 19.4500 |
| GO:0005615~extracellular space | CC_FAT | 61 | 20 | 0.0702 | | 204 | 685 | 12782 | ICAM1, A2M, FGF7, CCL2, LDLR, GNRH1, FBN1, GGH, MGP, IGF1, MMP2, VCAM1, TNFSF10, KAL1, TGFBI, GDF9, LOX, IGFBP3, FN1, IGFBP5 | 1.8294 | 0.0128 | 15.8486 |
| GO:0048407~platelet-derived growth factor binding | MF_FAT | 62 | 3 | 0.0105 | | 213 | 11 | 12983 | PDGFRA, COL6A1, COL1A1 | 16.6236 | 0.0132 | 17.4896 |
| GO:0000777~condensed chromosome kinetochore | CC_FAT | 63 | 5 | 0.0175 | | 204 | 58 | 12782 | MAD2L1, CENPV, NDC80, NUP37, CENPK | 5.4015 | 0.0134 | 16.5542 |
| GO:0051248~negative regulation of protein metabolic process | BP_FAT | 64 | 9 | 0.0316 | | 229 | 187 | 13528 | PSMB5, A2M, MAD2L1, NFKB1, ANAPC11, EIF2B3, IGFBP3, CDK5, IGFBP5 | 2.8431 | 0.0140 | 21.2680 |
| GO:0060323~head morphogenesis | BP_FAT | 65 | 3 | 0.0105 | | 229 | 11 | 13528 | PDGFRA, COL1A1, MMP2 | 16.1112 | 0.0141 | 21.3714 |
| GO:0060324~face development | BP_FAT | 66 | 3 | 0.0105 | | 229 | 11 | 13528 | PDGFRA, COL1A1, MMP2 | 16.1112 | 0.0141 | 21.3714 |
| GO:0000775~chromosome, centromeric region | CC_FAT | 67 | 7 | 0.0246 | | 204 | 124 | 12782 | MAD2L1, SGOL2, SGOL1, CENPV, NDC80, NUP37, CENPK | 3.5371 | 0.0142 | 17.3767 |
| GO:0032268~regulation of cellular protein metabolic process | BP_FAT | 68 | 16 | 0.0561 | | 229 | 474 | 13528 | A2M, IGF1, NFKB1, ANAPC11, CDK5, PSMB5, EDNRA, MAD2L1, DIO2, PIAS3, MTRF1, FBXO4, EIF2B3, IGFBP3, EIF4E2, IGFBP5 | 1.9941 | 0.0147 | 22.2577 |
| GO:0014910~regulation of smooth muscle cell migration | BP_FAT | 69 | 3 | 0.0105 | | 229 | 12 | 13528 | IGF1, IGFBP3, IGFBP5 | 14.7686 | 0.0167 | 24.8533 |
| GO:0000793~condensed chromosome | CC_FAT | 70 | 7 | 0.0246 | | 204 | 129 | 12782 | MAD2L1, SGOL2, SGOL1, CENPV, NDC80, NUP37, CENPK | 3.4000 | 0.0169 | 20.4109 |
| GO:0005581~collagen | CC_FAT | 71 | 4 | 0.0140 | | 204 | 35 | 12782 | COL6A1, COL1A1, LOX, COL5A2 | 7.1608 | 0.0178 | 21.3422 |
| GO:0005746~mitochondrial respiratory chain | CC_FAT | 72 | 5 | 0.0175 | | 204 | 64 | 12782 | NDUFA4, NDUFB6, NDUFB8, NDUFAB1, UQCRB | 4.8951 | 0.0187 | 22.3222 |
| GO:0008285~negative regulation of cell proliferation | BP_FAT | 73 | 13 | 0.0456 | | 229 | 361 | 13528 | ING5, ALDH1A2, BTG2, GNRH1, RBM5, SF1, FABP3, TP53, CDK6, SMAD2, GPNMB, IGFBP3, IGFBP5 | 2.1273 | 0.0195 | 28.4543 |
| GO:0042383~sarcolemma | CC_FAT | 74 | 5 | 0.0175 | | 204 | 67 | 12782 | EDNRA, KRT19, BGN, CAMK2D, COL6A1 | 4.6759 | 0.0218 | 25.4929 |
| GO:0060322~head development | BP_FAT | 75 | 3 | 0.0105 | | 229 | 14 | 13528 | PDGFRA, COL1A1, MMP2 | 12.6588 | 0.0225 | 32.0567 |
| GO:0048662~negative regulation of smooth muscle cell proliferation | BP_FAT | 76 | 3 | 0.0105 | | 229 | 14 | 13528 | SF1, IGFBP3, IGFBP5 | 12.6588 | 0.0225 | 32.0567 |
| GO:0010171~body morphogenesis | BP_FAT | 77 | 3 | 0.0105 | | 229 | 14 | 13528 | PDGFRA, COL1A1, MMP2 | 12.6588 | 0.0225 | 32.0567 |
| GO:0005576~extracellular region | CC_FAT | 78 | 44 | 0.1544 | | 204 | 2010 | 12782 | EMCN, A2M, FGF7, CCL2, LDLR, ENPP2, CD248, PAMR1, GABBR1, C1S, MMP2, VCAM1, KAL1, TGFBI, COL6A1, GDF9, SEMA3A, LOX, FN1, ICAM1, FLRT2, SVEP1, GNRH1, SPARCL1, FBN1, EFEMP1, GGH, MGP, IGF1, ACTN1, NID1, SERPINI1, COL5A2, ECM1, LCN2, TNFSF10, BGN, C15ORF61, FREM1, COL1A1, WDR1, IGFBP3, ADAMTS5, IGFBP5 | 1.3716 | 0.0229 | 26.6873 |
| GO:0006916~anti-apoptosis | BP_FAT | 79 | 9 | 0.0316 | | 229 | 206 | 13528 | CCL2, GNRH1, TGM2, IGF1, NFKB1, TNFAIP3, IKBKB, CASP2, TCF7L2 | 2.5809 | 0.0235 | 33.2174 |
| GO:0030334~regulation of cell migration | BP_FAT | 80 | 8 | 0.0281 | | 229 | 169 | 13528 | ICAM1, ENPP2, PDGFRA, IGF1, ABHD2, IGFBP3, CDK5, IGFBP5 | 2.7964 | 0.0244 | 34.2681 |
| GO:0042127~regulation of cell proliferation | BP_FAT | 81 | 22 | 0.0772 | | 229 | 787 | 13528 | ING5, FGF7, CCL2, GNRH1, RBM5, SF1, TP53, IGF1, CDK6, SMAD2, VCAM1, EDNRA, ALDH1A2, BTG2, PDGFRA, FABP3, CAMK2D, TGM2, GPNMB, IGFBP3, CCNA2, IGFBP5 | 1.6514 | 0.0250 | 34.9172 |
| GO:0051726~regulation of cell cycle | BP_FAT | 82 | 12 | 0.0421 | | 229 | 331 | 13528 | MAD2L1, TSPYL2, DLGAP5, CAMK2D, KIF20B, TP53, IGF1, CDK6, INTS3, GADD45B, CDK5, CCNA2 | 2.1417 | 0.0250 | 34.9347 |
| GO:0030324~lung development | BP_FAT | 83 | 6 | 0.0211 | | 229 | 99 | 13528 | ALDH1A2, FGF7, PDGFRA, MGP, SMAD2, LOX | 3.5803 | 0.0259 | 35.9858 |
| GO:0031093~platelet alpha granule lumen | CC_FAT | 84 | 4 | 0.0140 | | 204 | 41 | 12782 | A2M, IGF1, ACTN1, FN1 | 6.1129 | 0.0270 | 30.6962 |
| GO:0019838~growth factor binding | MF_FAT | 85 | 6 | 0.0211 | | 213 | 105 | 12983 | A2M, PDGFRA, COL6A1, COL1A1, IGFBP3, IGFBP5 | 3.4830 | 0.0287 | 34.3280 |
| GO:0005747~mitochondrial respiratory chain complex I | CC_FAT | 86 | 4 | 0.0140 | | 204 | 42 | 12782 | NDUFA4, NDUFB6, NDUFB8, NDUFAB1 | 5.9673 | 0.0288 | 32.3434 |
| GO:0030964~NADH dehydrogenase complex | CC_FAT | 87 | 4 | 0.0140 | | 204 | 42 | 12782 | NDUFA4, NDUFB6, NDUFB8, NDUFAB1 | 5.9673 | 0.0288 | 32.3434 |
| GO:0045271~respiratory chain complex I | CC_FAT | 88 | 4 | 0.0140 | | 204 | 42 | 12782 | NDUFA4, NDUFB6, NDUFB8, NDUFAB1 | 5.9673 | 0.0288 | 32.3434 |
| GO:0030323~respiratory tube development | BP_FAT | 89 | 6 | 0.0211 | | 229 | 102 | 13528 | ALDH1A2, FGF7, PDGFRA, MGP, SMAD2, LOX | 3.4750 | 0.0290 | 39.3417 |
| GO:0030198~extracellular matrix organization | BP_FAT | 90 | 6 | 0.0211 | | 229 | 104 | 13528 | TGFBI, PDGFRA, NID1, COL1A1, LOX, COL5A2 | 3.4081 | 0.0312 | 41.6129 |
| GO:0030892~mitotic cohesin complex | CC_FAT | 91 | 2 | 0.0070 | | 204 | 2 | 12782 | SGOL2, SGOL1 | 62.6569 | 0.0315 | 34.8392 |
| GO:0030246~carbohydrate binding | MF_FAT | 92 | 12 | 0.0421 | | 213 | 354 | 12983 | TNFAIP6, EMCN, FGF7, BGN, GALNT10, CCL2, ENPP2, PYGL, FREM1, CD248, GPNMB, FN1 | 2.0662 | 0.0316 | 37.0573 |
| GO:0043627~response to estrogen stimulus | BP_FAT | 93 | 6 | 0.0211 | | 229 | 105 | 13528 | ALDH1A2, KRT19, LDLR, PDGFRA, CRIPAK, CCNA2 | 3.3757 | 0.0323 | 42.7559 |
| GO:0060205~cytoplasmic membrane-bounded vesicle lumen | CC_FAT | 94 | 4 | 0.0140 | | 204 | 44 | 12782 | A2M, IGF1, ACTN1, FN1 | 5.6961 | 0.0325 | 35.6861 |
| GO:0050136~NADH dehydrogenase (quinone) activity | MF_FAT | 95 | 4 | 0.0140 | | 213 | 43 | 12983 | NDUFA4, NDUFB6, NDUFB8, NDUFAB1 | 5.6701 | 0.0328 | 38.2037 |
| GO:0008137~NADH dehydrogenase (ubiquinone) activity | MF_FAT | 96 | 4 | 0.0140 | | 213 | 43 | 12983 | NDUFA4, NDUFB6, NDUFB8, NDUFAB1 | 5.6701 | 0.0328 | 38.2037 |
| GO:0003954~NADH dehydrogenase activity | MF_FAT | 97 | 4 | 0.0140 | | 213 | 43 | 12983 | NDUFA4, NDUFB6, NDUFB8, NDUFAB1 | 5.6701 | 0.0328 | 38.2037 |
| GO:0070013~intracellular organelle lumen | CC_FAT | 98 | 39 | 0.1368 | | 204 | 1779 | 12782 | ING5, MRPS15, LIN9, RBM5, NDUFAB1, MED24, NFKB1, INTS3, ANAPC11, TCF7L2, KARS, MRPL20, TSPYL2, RPP30, MBNL3, GEMIN6, CCNA2, TSEN15, ELP4, POLR3G, ACTB, ZBTB20, SIRT4, SF1, TP53, CCNL1, ACTN1, SMAD2, NXF1, DACH1, PIAS3, SFPQ, POLDIP2, DDX50, ATP5C1, KIF20B, CIRBP, RBM14, VPS25 | 1.3736 | 0.0330 | 36.2094 |
| GO:0060346~bone trabecula formation | BP_FAT | 99 | 2 | 0.0070 | | 229 | 2 | 13528 | COL1A1, MMP2 | 59.0742 | 0.0334 | 43.8297 |
| GO:0006120~mitochondrial electron transport, NADH to ubiquinone | BP_FAT | 100 | 4 | 0.0140 | | 229 | 42 | 13528 | NDUFA4, NDUFB6, NDUFB8, NDUFAB1 | 5.6261 | 0.0335 | 43.9051 |
| GO:0000776~kinetochore | CC_FAT | 101 | 5 | 0.0175 | | 204 | 77 | 12782 | MAD2L1, CENPV, NDC80, NUP37, CENPK | 4.0686 | 0.0340 | 37.0773 |
| GO:0043122~regulation of I-kappaB kinase/NF-kappaB cascade | BP_FAT | 102 | 6 | 0.0211 | | 229 | 107 | 13528 | TNFSF10, REL, TGM2, TNFAIP3, IKBKB, ECM1 | 3.3126 | 0.0347 | 45.0522 |
| GO:0051276~chromosome organization | BP_FAT | 103 | 15 | 0.0526 | | 229 | 485 | 13528 | ING5, RAD51C, SGOL2, NASP, DLGAP5, ARID4B, TP53, NDC80, MAD2L1, TSPYL2, PHF1, CENPV, SETD7, FBXO4, RBM14 | 1.8270 | 0.0354 | 45.7888 |
| GO:0060541~respiratory system development | BP_FAT | 104 | 6 | 0.0211 | | 229 | 108 | 13528 | ALDH1A2, FGF7, PDGFRA, MGP, SMAD2, LOX | 3.2819 | 0.0359 | 46.2036 |
| GO:0031983~vesicle lumen | CC_FAT | 105 | 4 | 0.0140 | | 204 | 46 | 12782 | A2M, IGF1, ACTN1, FN1 | 5.4484 | 0.0364 | 39.0724 |
| GO:0008033~tRNA processing | BP_FAT | 106 | 5 | 0.0175 | | 229 | 76 | 13528 | SARS, RPP30, TSEN15, PUS7, TRMT61B | 3.8865 | 0.0393 | 49.3731 |
| GO:0006091~generation of precursor metabolites and energy | BP_FAT | 107 | 11 | 0.0386 | | 229 | 313 | 13528 | NDUFA4, SDHB, MDH1B, UQCR10, NDUFB6, PYGL, NDUFB8, NDUFAB1, ATP5C1, ATP5O, UQCRB | 2.0761 | 0.0395 | 49.5004 |
| GO:0030247~polysaccharide binding | MF_FAT | 108 | 7 | 0.0246 | | 213 | 154 | 12983 | TNFAIP6, FGF7, BGN, CCL2, ENPP2, GPNMB, FN1 | 2.7706 | 0.0406 | 45.0127 |
| GO:0001871~pattern binding | MF_FAT | 109 | 7 | 0.0246 | | 213 | 154 | 12983 | TNFAIP6, FGF7, BGN, CCL2, ENPP2, GPNMB, FN1 | 2.7706 | 0.0406 | 45.0127 |
| GO:0005654~nucleoplasm | CC_FAT | 110 | 22 | 0.0772 | | 204 | 882 | 12782 | ING5, ELP4, ACTB, POLR3G, LIN9, TP53, CCNL1, MED24, SMAD2, NFKB1, NXF1, DACH1, INTS3, ANAPC11, TCF7L2, PIAS3, KIF20B, CIRBP, GEMIN6, RBM14, CCNA2, VPS25 | 1.5629 | 0.0408 | 42.7556 |
| GO:0032403~protein complex binding | MF_FAT | 111 | 8 | 0.0281 | | 213 | 196 | 12983 | VCAM1, ICAM1, TGFBI, IGF1, ACTN1, ATP5O, GPNMB, ADAMTS5 | 2.4879 | 0.0419 | 46.0597 |
| GO:0007346~regulation of mitotic cell cycle | BP_FAT | 112 | 7 | 0.0246 | | 229 | 152 | 13528 | MAD2L1, DLGAP5, CAMK2D, KIF20B, TP53, IGF1, CCNA2 | 2.7205 | 0.0438 | 53.2663 |
| GO:0040012~regulation of locomotion | BP_FAT | 113 | 8 | 0.0281 | | 229 | 192 | 13528 | ICAM1, ENPP2, PDGFRA, IGF1, ABHD2, IGFBP3, CDK5, IGFBP5 | 2.4614 | 0.0441 | 53.4527 |
| GO:0015175~neutral amino acid transmembrane transporter activity | MF_FAT | 114 | 3 | 0.0105 | | 213 | 21 | 12983 | SLC6A6, SLC7A8, SLC3A2 | 8.7076 | 0.0455 | 48.8740 |
| GO:0016655~oxidoreductase activity, acting on NADH or NADPH, quinone or similar compound as acceptor | MF_FAT | 115 | 4 | 0.0140 | | 213 | 49 | 12983 | NDUFA4, NDUFB6, NDUFB8, NDUFAB1 | 4.9758 | 0.0457 | 49.0436 |
| GO:0043434~response to peptide hormone stimulus | BP_FAT | 116 | 7 | 0.0246 | | 229 | 154 | 13528 | BTG2, GGH, FABP3, COL1A1, PIK3R3, EIF2B3, CCNA2 | 2.6852 | 0.0462 | 55.1946 |
| GO:0045786~negative regulation of cell cycle | BP_FAT | 117 | 5 | 0.0175 | | 229 | 81 | 13528 | MAD2L1, TSPYL2, TP53, CDK6, CDK5 | 3.6466 | 0.0478 | 56.4710 |
| GO:0015804~neutral amino acid transport | BP_FAT | 118 | 3 | 0.0105 | | 229 | 21 | 13528 | SLC6A6, SLC7A8, SLC3A2 | 8.4392 | 0.0481 | 56.6979 |
| GO:0006399~tRNA metabolic process | BP_FAT | 119 | 6 | 0.0211 | | 229 | 118 | 13528 | SARS, RPP30, TSEN15, PUS7, TRMT61B, KARS | 3.0038 | 0.0493 | 57.6201 |
| GO:0051177~meiotic sister chromatid cohesion | BP_FAT | 120 | 2 | 0.0070 | | 229 | 3 | 13528 | RAD51C, SGOL2 | 39.3828 | 0.0497 | 57.9035 |
| GO:0016310~phosphorylation | BP_FAT | 121 | 21 | 0.0737 | | 229 | 800 | 13528 | NDUFA4, CCL2, NDUFB6, NDUFB8, NDUFAB1, CDK6, SMAD2, PBK, ULK4, CDK5, RPS6KA6, UQCR10, PDGFRA, CAMK2D, ATP5C1, ATP5O, GADD45B, IKBKB, IGFBP3, MYLK, UQCRB | 1.5507 | 0.0503 | 58.3074 |
| GO:0031981~nuclear lumen | CC_FAT | 122 | 32 | 0.1123 | | 204 | 1450 | 12782 | ING5, LIN9, RBM5, MED24, NFKB1, ANAPC11, INTS3, TCF7L2, TSPYL2, RPP30, MBNL3, GEMIN6, CCNA2, TSEN15, ACTB, ELP4, POLR3G, ZBTB20, SF1, TP53, CCNL1, ACTN1, SMAD2, NXF1, DACH1, PIAS3, SFPQ, DDX50, KIF20B, CIRBP, RBM14, VPS25 | 1.3828 | 0.0521 | 51.1015 |
| GO:0006412~translation | BP_FAT | 123 | 11 | 0.0386 | | 229 | 331 | 13528 | QRSL1, MRPS15, DIO2, RPL34, SARS, MTRF1, RPL22L1, EIF2B3, EIF4E2, KARS, MRPL20 | 1.9632 | 0.0545 | 61.3782 |
| GO:0005741~mitochondrial outer membrane | CC_FAT | 124 | 5 | 0.0175 | | 204 | 90 | 12782 | CISD1, MAOB, TOMM22, VDAC3, CPT1A | 3.4809 | 0.0550 | 53.0654 |
| GO:0009154~purine ribonucleotide catabolic process | BP_FAT | 125 | 3 | 0.0105 | | 229 | 23 | 13528 | NUDT9, ATP5O, GCH1 | 7.7053 | 0.0567 | 62.8817 |
| GO:0044451~nucleoplasm part | CC_FAT | 126 | 15 | 0.0526 | | 204 | 555 | 12782 | ING5, ACTB, POLR3G, ELP4, TP53, CCNL1, MED24, SMAD2, DACH1, INTS3, NXF1, TCF7L2, PIAS3, GEMIN6, RBM14 | 1.6934 | 0.0591 | 55.7346 |
| GO:0031091~platelet alpha granule | CC_FAT | 127 | 4 | 0.0140 | | 204 | 56 | 12782 | A2M, IGF1, ACTN1, FN1 | 4.4755 | 0.0593 | 55.8371 |
| GO:0010001~glial cell differentiation | BP_FAT | 128 | 4 | 0.0140 | | 229 | 53 | 13528 | IGF1, C1S, EIF2B3, CDK5 | 4.4584 | 0.0599 | 64.9041 |
| GO:0005343~organic acid:sodium symporter activity | MF_FAT | 129 | 3 | 0.0105 | | 213 | 25 | 12983 | SLC6A6, SLC1A1, SLC10A1 | 7.3144 | 0.0623 | 60.4234 |
| GO:0007160~cell-matrix adhesion | BP_FAT | 130 | 5 | 0.0175 | | 229 | 89 | 13528 | FREM1, ACTN1, NID1, CDK5, FN1 | 3.3188 | 0.0634 | 67.0954 |
| GO:0046930~pore complex | CC_FAT | 131 | 5 | 0.0175 | | 204 | 95 | 12782 | MAD2L1, NUP93, NUP37, NXF1, VDAC3 | 3.2977 | 0.0645 | 59.0271 |
| GO:0014912~negative regulation of smooth muscle cell migration | BP_FAT | 132 | 2 | 0.0070 | | 229 | 4 | 13528 | IGFBP3, IGFBP5 | 29.5371 | 0.0657 | 68.4517 |
| GO:0009261~ribonucleotide catabolic process | BP_FAT | 133 | 3 | 0.0105 | | 229 | 25 | 13528 | NUDT9, ATP5O, GCH1 | 7.0889 | 0.0658 | 68.5078 |
| GO:0031090~organelle membrane | CC_FAT | 134 | 25 | 0.0877 | | 204 | 1096 | 12782 | CLTA, STX8, NDUFB6, LDLR, NDUFB8, NDUFAB1, RABEPK, EDNRA, UQCR10, CISD1, PIGM, CAMK2D, ATP5O, SEC61A1, NDUFA4, MAOB, VDAC3, CPT1A, SDHB, ATP5C1, TOMM22, SPCS2, TMPO, UQCRB, VPS25 | 1.4292 | 0.0666 | 60.2212 |
| GO:0004437~inositol or phosphatidylinositol phosphatase activity | MF_FAT | 135 | 3 | 0.0105 | | 213 | 26 | 12983 | IMPAD1, MTMR1, INPP4B | 7.0330 | 0.0668 | 63.0660 |
| GO:0009612~response to mechanical stimulus | BP_FAT | 136 | 4 | 0.0140 | | 229 | 56 | 13528 | CCL2, BTG2, MGP, COL1A1 | 4.2196 | 0.0683 | 69.8966 |
| GO:0007088~regulation of mitosis | BP_FAT | 137 | 4 | 0.0140 | | 229 | 56 | 13528 | MAD2L1, DLGAP5, KIF20B, IGF1 | 4.2196 | 0.0683 | 69.8966 |
| GO:0051783~regulation of nuclear division | BP_FAT | 138 | 4 | 0.0140 | | 229 | 56 | 13528 | MAD2L1, DLGAP5, KIF20B, IGF1 | 4.2196 | 0.0683 | 69.8966 |
| GO:0050840~extracellular matrix binding | MF_FAT | 139 | 3 | 0.0105 | | 213 | 27 | 12983 | BGN, TGFBI, NID1 | 6.7726 | 0.0713 | 65.5991 |
| GO:0015171~amino acid transmembrane transporter activity | MF_FAT | 140 | 4 | 0.0140 | | 213 | 59 | 12983 | SLC6A6, SLC7A8, SLC3A2, SLC1A1 | 4.1324 | 0.0718 | 65.8170 |
| GO:0030175~filopodium | CC_FAT | 141 | 3 | 0.0105 | | 204 | 28 | 12782 | FAM65B, VCAM1, CDK5 | 6.7132 | 0.0725 | 63.4375 |
| GO:0043066~negative regulation of apoptosis | BP_FAT | 142 | 11 | 0.0386 | | 229 | 354 | 13528 | CCL2, BTG2, GNRH1, TGM2, TP53, IGF1, NFKB1, TNFAIP3, IKBKB, CASP2, TCF7L2 | 1.8356 | 0.0773 | 74.4593 |
| GO:0044433~cytoplasmic vesicle part | CC_FAT | 143 | 7 | 0.0246 | | 204 | 187 | 12782 | A2M, CLTA, LDLR, CAMK2D, IGF1, ACTN1, FN1 | 2.3454 | 0.0779 | 66.2074 |
| GO:0035295~tube development | BP_FAT | 144 | 8 | 0.0281 | | 229 | 220 | 13528 | EDNRA, ALDH1A2, FGF7, PDGFRA, IGF1, MGP, SMAD2, LOX | 2.1482 | 0.0790 | 75.2506 |
| GO:0006396~RNA processing | BP_FAT | 145 | 15 | 0.0526 | | 229 | 547 | 13528 | SARS, PNPT1, RBM5, SF1, SMAD2, INTS3, PUS7, TRMT61B, SFPQ, RPP30, DHX35, GEMIN6, LSM1, RBM14, TSEN15 | 1.6200 | 0.0790 | 75.2516 |
| GO:0005539~glycosaminoglycan binding | MF_FAT | 146 | 6 | 0.0211 | | 213 | 140 | 12983 | TNFAIP6, FGF7, BGN, CCL2, GPNMB, FN1 | 2.6123 | 0.0794 | 69.6669 |
| GO:0045785~positive regulation of cell adhesion | BP_FAT | 147 | 4 | 0.0140 | | 229 | 60 | 13528 | TGM2, CDK6, NID1, CYTH3 | 3.9383 | 0.0804 | 75.8742 |
| GO:0034621~cellular macromolecular complex subunit organization | BP_FAT | 148 | 11 | 0.0386 | | 229 | 357 | 13528 | TSPYL2, NASP, MTRF1, RBM5, CENPV, SF1, TOMM22, ARPC4, SMAD2, GEMIN6, CDK5 | 1.8202 | 0.0806 | 75.9860 |
| GO:0022614~membrane to membrane docking | BP_FAT | 149 | 2 | 0.0070 | | 229 | 5 | 13528 | VCAM1, ICAM1 | 23.6297 | 0.0815 | 76.3573 |
| GO:0001957~intramembranous ossification | BP_FAT | 150 | 2 | 0.0070 | | 229 | 5 | 13528 | COL1A1, MMP2 | 23.6297 | 0.0815 | 76.3573 |
| GO:0043069~negative regulation of programmed cell death | BP_FAT | 151 | 11 | 0.0386 | | 229 | 359 | 13528 | CCL2, BTG2, GNRH1, TGM2, TP53, IGF1, NFKB1, TNFAIP3, IKBKB, CASP2, TCF7L2 | 1.8101 | 0.0824 | 76.7497 |
| GO:0031589~cell-substrate adhesion | BP_FAT | 152 | 5 | 0.0175 | | 229 | 98 | 13528 | FREM1, ACTN1, NID1, CDK5, FN1 | 3.0140 | 0.0837 | 77.3173 |
| GO:0031968~organelle outer membrane | CC_FAT | 153 | 5 | 0.0175 | | 204 | 104 | 12782 | CISD1, MAOB, TOMM22, VDAC3, CPT1A | 3.0123 | 0.0837 | 68.9587 |
| GO:0060548~negative regulation of cell death | BP_FAT | 154 | 11 | 0.0386 | | 229 | 360 | 13528 | CCL2, BTG2, GNRH1, TGM2, TP53, IGF1, NFKB1, TNFAIP3, IKBKB, CASP2, TCF7L2 | 1.8050 | 0.0845 | 77.6519 |
| GO:0030199~collagen fibril organization | BP_FAT | 155 | 3 | 0.0105 | | 229 | 29 | 13528 | COL1A1, LOX, COL5A2 | 6.1111 | 0.0853 | 77.9780 |
| GO:0006275~regulation of DNA replication | BP_FAT | 156 | 4 | 0.0140 | | 229 | 62 | 13528 | TSPYL2, PDGFRA, TP53, IGF1 | 3.8112 | 0.0868 | 78.5549 |
| GO:0031396~regulation of protein ubiquitination | BP_FAT | 157 | 5 | 0.0175 | | 229 | 100 | 13528 | PSMB5, MAD2L1, FBXO4, ANAPC11, CDK5 | 2.9537 | 0.0886 | 79.2897 |
| GO:0044427~chromosomal part | CC_FAT | 158 | 11 | 0.0386 | | 204 | 386 | 12782 | ATRX, MAD2L1, SGOL2, ARID4B, SGOL1, CENPV, TP53, NDC80, NUP37, TMPO, CENPK | 1.7856 | 0.0888 | 71.1855 |
| GO:0051271~negative regulation of cell motion | BP_FAT | 159 | 4 | 0.0140 | | 229 | 63 | 13528 | ACTN1, ABHD2, IGFBP3, IGFBP5 | 3.7507 | 0.0900 | 79.8168 |
| GO:0045261~proton-transporting ATP synthase complex, catalytic core F(1) | CC_FAT | 160 | 2 | 0.0070 | | 204 | 6 | 12782 | ATP5C1, ATP5O | 20.8856 | 0.0916 | 72.3388 |
| GO:0043933~macromolecular complex subunit organization | BP_FAT | 161 | 18 | 0.0632 | | 229 | 710 | 13528 | NASP, ATL3, RBM5, SF1, TP53, MED24, MGP, ARPC4, SMAD2, CDK5, GCH1, TSPYL2, MTRF1, CENPV, TGM2, TOMM22, GEMIN6, SLC1A1 | 1.4977 | 0.0918 | 80.4836 |
| GO:0019867~outer membrane | CC_FAT | 162 | 5 | 0.0175 | | 204 | 108 | 12782 | CISD1, MAOB, TOMM22, VDAC3, CPT1A | 2.9008 | 0.0931 | 72.9361 |
| GO:0009628~response to abiotic stimulus | BP_FAT | 163 | 11 | 0.0386 | | 229 | 368 | 13528 | CCL2, DIO2, BTG2, SLC2A1, TP53, CIRBP, MGP, INTS3, COL1A1, EIF2B3, CDK5 | 1.7658 | 0.0937 | 81.1474 |
| GO:0031994~insulin-like growth factor I binding | MF_FAT | 164 | 2 | 0.0070 | | 213 | 6 | 12983 | IGFBP3, IGFBP5 | 20.3177 | 0.0941 | 75.9344 |
| GO:0022406~membrane docking | BP_FAT | 165 | 3 | 0.0105 | | 229 | 31 | 13528 | VCAM1, ICAM1, RABEPK | 5.7169 | 0.0956 | 81.8334 |
| GO:0051187~cofactor catabolic process | BP_FAT | 166 | 3 | 0.0105 | | 229 | 31 | 13528 | BLVRA, SDHB, MDH1B | 5.7169 | 0.0956 | 81.8334 |
| GO:0005198~structural molecule activity | MF_FAT | 167 | 16 | 0.0561 | | 213 | 634 | 12983 | ACTB, CLTA, MRPS15, FBN1, MGP, RPL22L1, COL5A2, MRPL20, KRT19, BGN, KRT80, RPL34, KAL1, COL1A1, CSTA, FN1 | 1.5382 | 0.0959 | 76.6272 |
| GO:0034470~ncRNA processing | BP_FAT | 168 | 7 | 0.0246 | | 229 | 187 | 13528 | SARS, RPP30, SMAD2, INTS3, TSEN15, PUS7, TRMT61B | 2.2113 | 0.0967 | 82.1800 |
| GO:0006122~mitochondrial electron transport, ubiquinol to cytochrome c | BP_FAT | 169 | 2 | 0.0070 | | 229 | 6 | 13528 | UQCR10, UQCRB | 19.6914 | 0.0970 | 82.2823 |
| GO:0043086~negative regulation of catalytic activity | BP_FAT | 170 | 9 | 0.0316 | | 229 | 277 | 13528 | PSMB5, MAD2L1, GABBR1, TP53, NFKB1, CRIPAK, ANAPC11, CSTA, GADD45B | 1.9194 | 0.0972 | 82.3478 |
| GO:0006796~phosphate metabolic process | BP_FAT | 171 | 23 | 0.0807 | | 229 | 973 | 13528 | NDUFA4, CCL2, NDUFB6, ENPP2, NDUFB8, NDUFAB1, CDK6, SMAD2, PBK, ULK4, CDK5, RPS6KA6, UQCR10, MTMR1, PDGFRA, CAMK2D, ATP5C1, ATP5O, GADD45B, IKBKB, IGFBP3, MYLK, UQCRB | 1.3964 | 0.0972 | 82.3577 |
| GO:0006793~phosphorus metabolic process | BP_FAT | 172 | 23 | 0.0807 | | 229 | 973 | 13528 | NDUFA4, CCL2, NDUFB6, ENPP2, NDUFB8, NDUFAB1, CDK6, SMAD2, PBK, ULK4, CDK5, RPS6KA6, UQCR10, MTMR1, PDGFRA, CAMK2D, ATP5C1, ATP5O, GADD45B, IKBKB, IGFBP3, MYLK, UQCRB | 1.3964 | 0.0972 | 82.3577 |
| GO:0006350~transcription | BP_FAT | 173 | 44 | 0.1544 | | 229 | 2101 | 13528 | ING5, HLF, GTF3A, TSHZ3, ARID4B, MED24, NFKB1, SP110, TCF7L2, ZNF512, MAX, GABPB1, TSPYL2, NR1D1, REL, ZNF709, ELOF1, ZNF124, POLR3G, ELP4, NFKBIZ, ZBTB20, RXRB, SF1, CCNL1, TP53, ZFP1, TLE4, SMAD2, DACH1, ZBTB45, HOXB3, BTG2, PHF1, PIAS3, SFPQ, IRF1, HIVEP2, SETD7, DENND4A, RFX3, PRDM1, RBM14, VPS25 | 1.2372 | 0.0977 | 82.5165 |

**Table S5. The result of DAVID pathway enrichment for 869 DEGs from Muscle2.**

| **Path_term** | **Num_of_symbols_in_list_in_pathway** | **Percentage_of_symbols_in_list** | **Num_of _symbols_in_bglist_in_pathway** | **Num_of_symbols_in_list** | **Num_of_symbols_in_bglist** | **Symbols_in_list** | **Fold_enrichment** | **P value** | **FDR** |
| --- | --- | --- | --- | --- | --- | --- | --- | --- | --- |
| hsa04010:MAPK signaling pathway | 21 | 0.0297 | 198 | 267 | 5085 | FGFR1, FGFR4, FGF7, TAOK2, MAP2K2, FGF17, MAPKAPK3, PPP3R1, CACNG4, FGF22, PPM1B, TGFB2, MAP3K6, NRAS, DUSP3, MAPK14, JUND, PDGFRA, EGF, DUSP8, CHUK | 2.0199 | 0.0032 | 3.7561 |
| hsa05218:Melanoma | 9 | 0.0127 | 198 | 71 | 5085 | FGFR1, NRAS, E2F3, FGF7, MAP2K2, FGF17, PDGFRA, FGF22, EGF | 3.2554 | 0.0058 | 6.6599 |
| hsa04810:Regulation of actin cytoskeleton | 17 | 0.0241 | 198 | 215 | 5085 | FGFR1, FGFR4, FGF7, MYL5, MAP2K2, FGF17, MYL10, FGF22, ACTN3, VCL, NRAS, CHRM3, ITGA7, PDGFRA, TMSB4Y, EGF, FN1 | 2.0307 | 0.0087 | 9.8491 |
| hsa05200:Pathways in cancer | 22 | 0.0312 | 198 | 328 | 5085 | FGFR1, E2F3, RET, IL6, FGF7, MSH3, MAP2K2, ERBB2, FGF17, FGF22, TGFB2, JUP, NRAS, LAMB3, WNT4, PLCG1, ETS1, PAX8, PDGFRA, EGF, CHUK, FN1 | 1.7226 | 0.0149 | 16.4110 |
| hsa04520:Adherens junction | 8 | 0.0113 | 198 | 77 | 5085 | PTPN6, FGFR1, ERBB2, SSX2IP, ACTN3, SNAI2, SRC, VCL | 2.6682 | 0.0287 | 29.3174 |
| hsa05214:Glioma | 7 | 0.0099 | 198 | 63 | 5085 | NRAS, E2F3, PLCG1, MAP2K2, PDGFRA, CALML6, EGF | 2.8535 | 0.0341 | 33.8620 |
| hsa05215:Prostate cancer | 8 | 0.0113 | 198 | 89 | 5085 | FGFR1, NRAS, E2F3, MAP2K2, ERBB2, PDGFRA, EGF, CHUK | 2.3085 | 0.0560 | 49.6981 |
| hsa05223:Non-small cell lung cancer | 6 | 0.0085 | 198 | 54 | 5085 | NRAS, E2F3, PLCG1, MAP2K2, ERBB2, EGF | 2.8535 | 0.0566 | 50.1096 |
| hsa00562:Inositol phosphate metabolism | 6 | 0.0085 | 198 | 54 | 5085 | ALDH6A1, CDIPT, PLCG1, PLCB2, IPMK, INPP5A | 2.8535 | 0.0566 | 50.1096 |
| hsa04370:VEGF signaling pathway | 7 | 0.0099 | 198 | 75 | 5085 | NRAS, PLCG1, MAP2K2, MAPK14, PPP3R1, MAPKAPK3, SRC | 2.3970 | 0.0693 | 57.5555 |
| hsa04662:B cell receptor signaling pathway | 7 | 0.0099 | 198 | 75 | 5085 | PTPN6, NRAS, NFKBIE, MAP2K2, FCGR2C, PPP3R1, CHUK | 2.3970 | 0.0693 | 57.5555 |
| hsa04530:Tight junction | 10 | 0.0142 | 198 | 134 | 5085 | NRAS, MYL5, MYH1, MYH4, MYH13, MYL10, ACTN3, MYH8, SRC, CLDN15 | 1.9166 | 0.0746 | 60.3164 |
| hsa05219:Bladder cancer | 5 | 0.0071 | 198 | 42 | 5085 | NRAS, E2F3, MAP2K2, ERBB2, EGF | 3.0574 | 0.0779 | 62.0037 |
| hsa04020:Calcium signaling pathway | 12 | 0.0170 | 198 | 176 | 5085 | ATP2B2, P2RX6, PLCG1, CHRM3, PHKG1, ERBB2, RYR3, PDGFRA, PPP3R1, CALML6, ITPR3, PLCB2 | 1.7510 | 0.0784 | 62.2339 |
| hsa04670:Leukocyte transendothelial migration | 9 | 0.0127 | 198 | 118 | 5085 | ICAM1, MYL5, PLCG1, MAPK14, NCF4, MYL10, ACTN3, VCL, CLDN15 | 1.9588 | 0.0860 | 65.8026 |
| hsa04510:Focal adhesion | 13 | 0.0184 | 198 | 201 | 5085 | LAMB3, MYL5, COMP, ERBB2, ITGA7, PDGFRA, MYL10, ACTN3, ZYX, EGF, SRC, VCL, FN1 | 1.6610 | 0.0887 | 66.9720 |
| hsa00280:Valine, leucine and isoleucine degradation | 5 | 0.0071 | 198 | 44 | 5085 | ALDH6A1, ALDH1B1, OXCT1, MCEE, BCKDHB | 2.9184 | 0.0891 | 67.1635 |

**Table S6. The result of DAVID pathway enrichment for 287 DEGs from PCOS6.**

| **Path_term** | **Num_of_symbols_in_list_in_pathway** | **Percentage_of_symbols_in_list** | **Num_of _symbols_in_bglist_in_pathway** | **Num_of_symbols_in_list** | **Num_of_symbols_in_bglist** | **Symbols_in_list** | **Fold_enrichment** | **P value** | **FDR** |
| --- | --- | --- | --- | --- | --- | --- | --- | --- | --- |
| hsa05016:Huntington's disease | 13 | 0.0456 | 102 | 180 | 5085 | NDUFA4, SDHB, UQCR10, CLTA, NDUFB6, NDUFB8, TGM2, ATP5C1, NDUFAB1, TP53, ATP5O, VDAC3, UQCRB | 3.6005 | 0.0002 | 0.2327 |
| hsa05222:Small cell lung cancer | 9 | 0.0316 | 102 | 84 | 5085 | MAX, PIAS3, RXRB, TP53, CDK6, NFKB1, PIK3R3, IKBKB, FN1 | 5.3414 | 0.0002 | 0.2629 |
| hsa05200:Pathways in cancer | 17 | 0.0596 | 102 | 328 | 5085 | FGF7, RXRB, CBL, TP53, IGF1, CDK6, NFKB1, SMAD2, MMP2, TCF7L2, MAX, PIAS3, SLC2A1, PDGFRA, IKBKB, PIK3R3, FN1 | 2.5838 | 0.0006 | 0.7372 |
| hsa05012:Parkinson's disease | 10 | 0.0351 | 102 | 128 | 5085 | NDUFA4, SDHB, UQCR10, NDUFB6, NDUFB8, NDUFAB1, ATP5C1, ATP5O, VDAC3, UQCRB | 3.8948 | 0.0009 | 1.0312 |
| hsa00190:Oxidative phosphorylation | 9 | 0.0316 | 102 | 130 | 5085 | NDUFA4, SDHB, UQCR10, NDUFB6, NDUFB8, NDUFAB1, ATP5C1, ATP5O, UQCRB | 3.4514 | 0.0040 | 4.5354 |
| hsa05010:Alzheimer's disease | 10 | 0.0351 | 102 | 163 | 5085 | NDUFA4, SDHB, UQCR10, NDUFB6, NDUFB8, NDUFAB1, ATP5C1, ATP5O, CDK5, UQCRB | 3.0585 | 0.0048 | 5.3423 |
| hsa04510:Focal adhesion | 11 | 0.0386 | 102 | 201 | 5085 | ACTB, DOCK1, PDGFRA, COL6A1, IGF1, ACTN1, COL1A1, PIK3R3, COL5A2, MYLK, FN1 | 2.7283 | 0.0062 | 6.8759 |
| hsa05214:Glioma | 6 | 0.0211 | 102 | 63 | 5085 | CAMK2D, PDGFRA, TP53, IGF1, CDK6, PIK3R3 | 4.7479 | 0.0079 | 8.6868 |
| hsa05215:Prostate cancer | 7 | 0.0246 | 102 | 89 | 5085 | PDGFRA, TP53, IGF1, NFKB1, PIK3R3, IKBKB, TCF7L2 | 3.9210 | 0.0081 | 8.9244 |
| hsa05218:Melanoma | 6 | 0.0211 | 102 | 71 | 5085 | FGF7, PDGFRA, TP53, IGF1, CDK6, PIK3R3 | 4.2129 | 0.0129 | 13.8467 |
| hsa05212:Pancreatic cancer | 6 | 0.0211 | 102 | 72 | 5085 | TP53, CDK6, NFKB1, SMAD2, PIK3R3, IKBKB | 4.1544 | 0.0137 | 14.5946 |
| hsa05220:Chronic myeloid leukemia | 6 | 0.0211 | 102 | 75 | 5085 | CBL, TP53, CDK6, NFKB1, PIK3R3, IKBKB | 3.9882 | 0.0161 | 16.9726 |
| hsa04114:Oocyte meiosis | 7 | 0.0246 | 102 | 110 | 5085 | PPP2R1B, RPS6KA6, MAD2L1, SGOL1, CAMK2D, IGF1, ANAPC11 | 3.1725 | 0.0215 | 22.0340 |
| hsa04914:Progesterone-mediated oocyte maturation | 6 | 0.0211 | 102 | 86 | 5085 | RPS6KA6, MAD2L1, IGF1, ANAPC11, PIK3R3, CCNA2 | 3.4781 | 0.0274 | 27.2779 |
| hsa04110:Cell cycle | 7 | 0.0246 | 102 | 125 | 5085 | MAD2L1, TP53, CDK6, SMAD2, ANAPC11, GADD45B, CCNA2 | 2.7918 | 0.0372 | 35.2156 |
| hsa04920:Adipocytokine signaling pathway | 5 | 0.0175 | 102 | 67 | 5085 | RXRB, SLC2A1, NFKB1, IKBKB, CPT1A | 3.7204 | 0.0434 | 39.7840 |
| hsa04115:p53 signaling pathway | 5 | 0.0175 | 102 | 68 | 5085 | TP53, IGF1, CDK6, GADD45B, IGFBP3 | 3.6657 | 0.0454 | 41.2311 |
| hsa04810:Regulation of actin cytoskeleton | 9 | 0.0316 | 102 | 215 | 5085 | ACTB, FGF7, DOCK1, PDGFRA, ACTN1, ARPC4, PIK3R3, MYLK, FN1 | 2.0869 | 0.0627 | 52.3546 |
| hsa04150:mTOR signaling pathway | 4 | 0.0140 | 102 | 52 | 5085 | RPS6KA6, IGF1, PIK3R3, EIF4E2 | 3.8348 | 0.0836 | 63.1906 |
| hsa04670:Leukocyte transendothelial migration | 6 | 0.0211 | 102 | 118 | 5085 | VCAM1, ACTB, ICAM1, ACTN1, PIK3R3, MMP2 | 2.5349 | 0.0846 | 63.6255 |
| hsa05210:Colorectal cancer | 5 | 0.0175 | 102 | 84 | 5085 | PDGFRA, TP53, SMAD2, PIK3R3, TCF7L2 | 2.9674 | 0.0849 | 63.7870 |
| hsa05223:Non-small cell lung cancer | 4 | 0.0140 | 102 | 54 | 5085 | RXRB, TP53, CDK6, PIK3R3 | 3.6928 | 0.0913 | 66.5613 |
| hsa04210:Apoptosis | 5 | 0.0175 | 102 | 87 | 5085 | TNFSF10, TP53, NFKB1, PIK3R3, IKBKB | 2.8651 | 0.0938 | 67.5928 |
| hsa04623:Cytosolic DNA-sensing pathway | 4 | 0.0140 | 102 | 55 | 5085 | DDX58, POLR3G, NFKB1, IKBKB | 3.6257 | 0.0952 | 68.1816 |
| hsa04722:Neurotrophin signaling pathway | 6 | 0.0211 | 102 | 124 | 5085 | RPS6KA6, CAMK2D, TP53, NFKB1, PIK3R3, IKBKB | 2.4122 | 0.0994 | 69.8227 |
